# Supplementary material for: NRAC controls CD36-mediated fatty acid uptake in adipocytes and lipid clearance in vivo
Source: EMBO J. 2025 Aug 1;44(18):5037–65. doi: 10.1038/s44318-025-00520-2 (PMC12436663; doi:10.1038/s44318-025-00520-2)
Supplement: Supplementary file 1 — Table EV1 [file 44318_2025_520_MOESM1_ESM.docx]

**Table EV1:** List of qPCR primers.

| **Gene Name** | **Forward** | **Reverse** |
| --- | --- | --- |
| A530016L24Rik (Nrac) | GGTAGCCCCTTCGGAAAGAC | CGGACCTGGTGGATTTCTGT |
| IL6 | GTCGGAGGCTTAATTACACATGT | CAAGTGCATCATCGTTGTTCA |
| TNFa | CCCACGTCGTAGCAAACCA | GTCTTTGAGATCCATGCCGTTG |
| *Ucp1* | CTGCCAGGACAGTACCCAAG | TCAGCTGTTCAAAGCACACA |
| *Tbp* | ACCCTTCACCAATGACTCCTATG | TGACTGCAGCAAATCGCTTGG |
