# Supplementary figures and images for: NRAC controls CD36-mediated fatty acid uptake in adipocytes and lipid clearance in vivo (part 1 of 2)
Source: EMBO J. 2025 Aug 1;44(18):5037–65. doi: 10.1038/s44318-025-00520-2 (PMC12436663; doi:10.1038/s44318-025-00520-2)

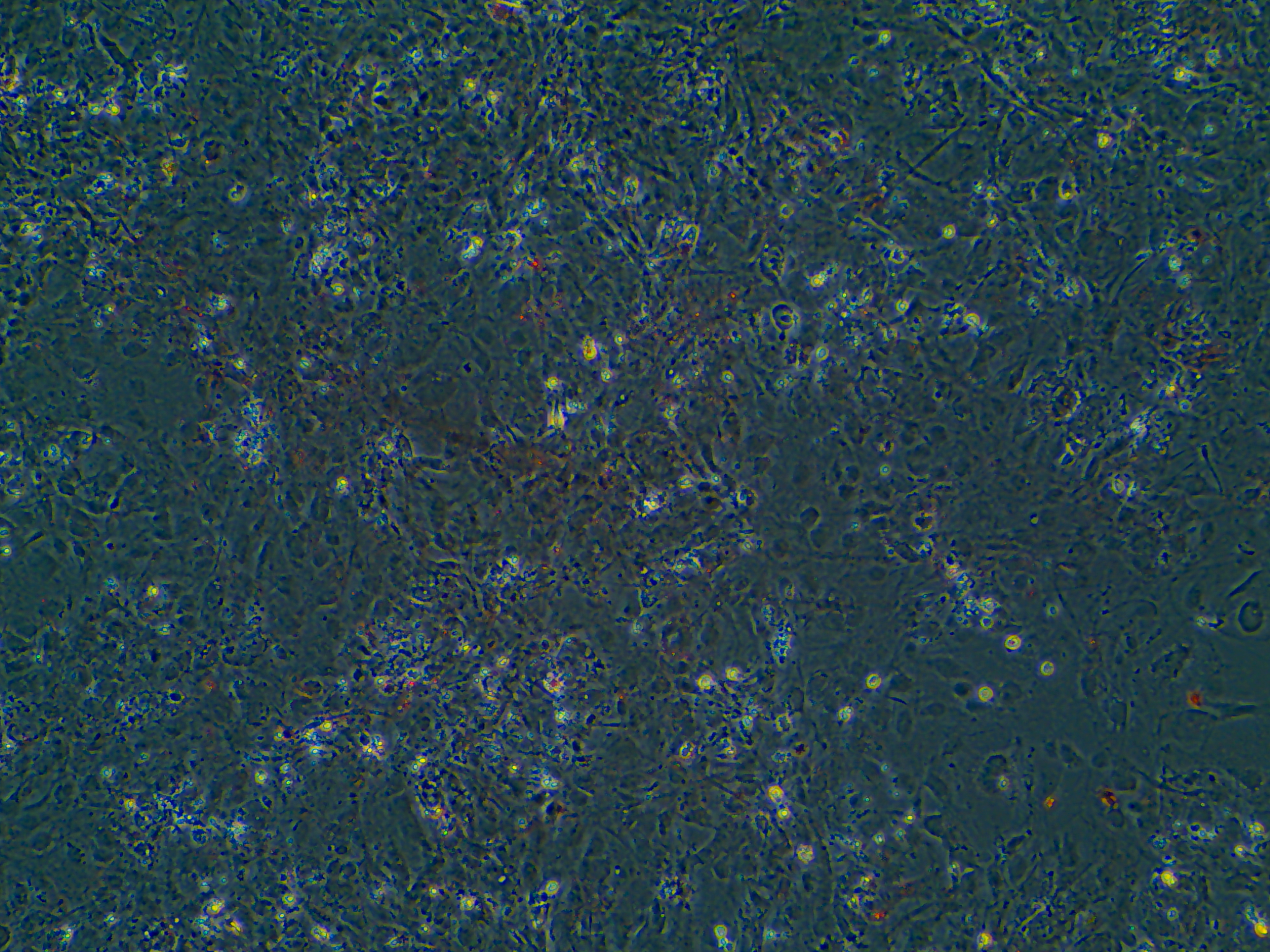

Supplement: Supplementary file 3 — Source data Fig. 1 [file 44318_2025_520_MOESM3_ESM.zip › Figure 1/1R/SC ko 10x Day 0.tif]

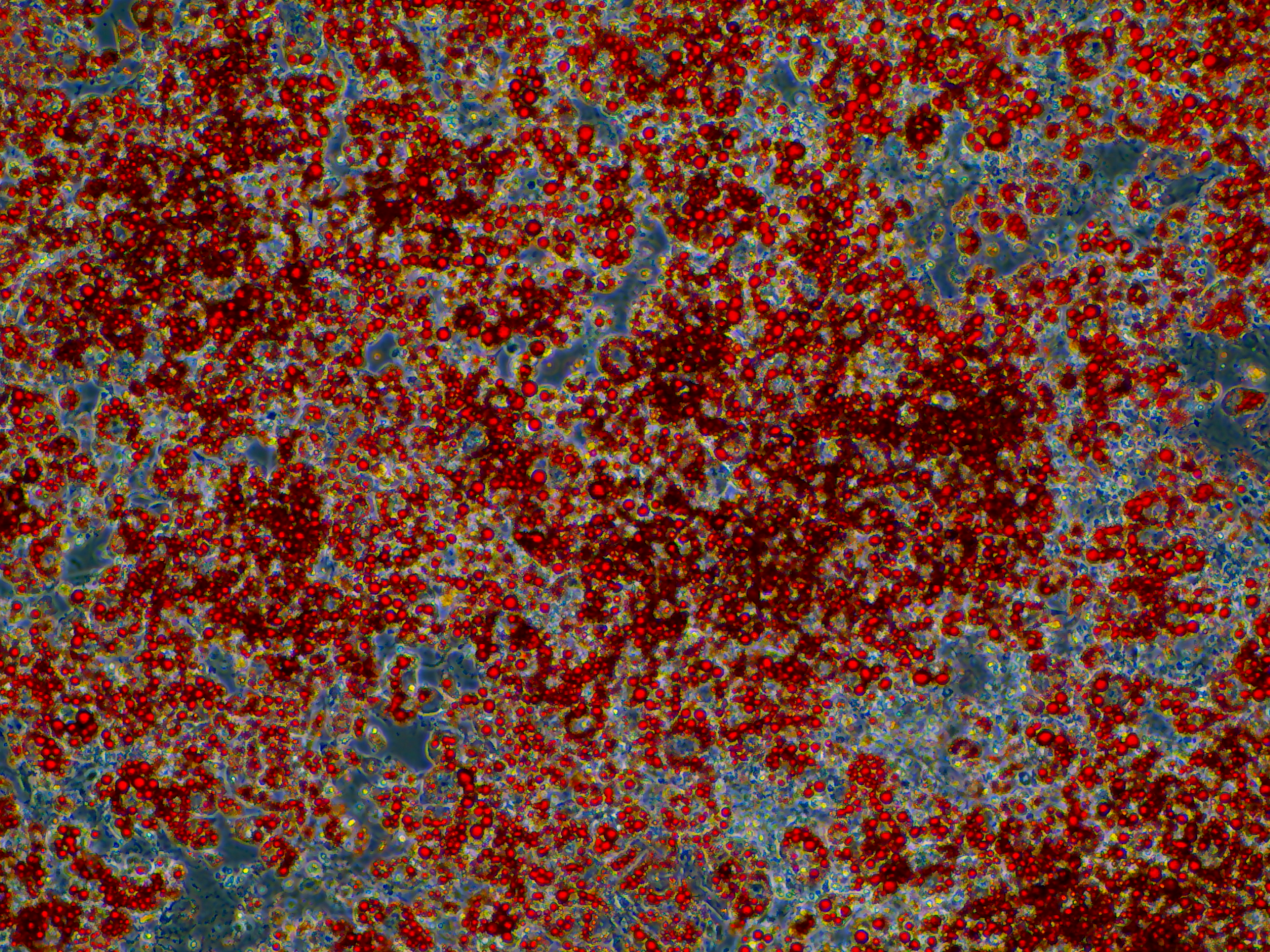

Supplement: Supplementary file 3 — Source data Fig. 1 [file 44318_2025_520_MOESM3_ESM.zip › Figure 1/1R/SC wt 10x Day 8.tif]

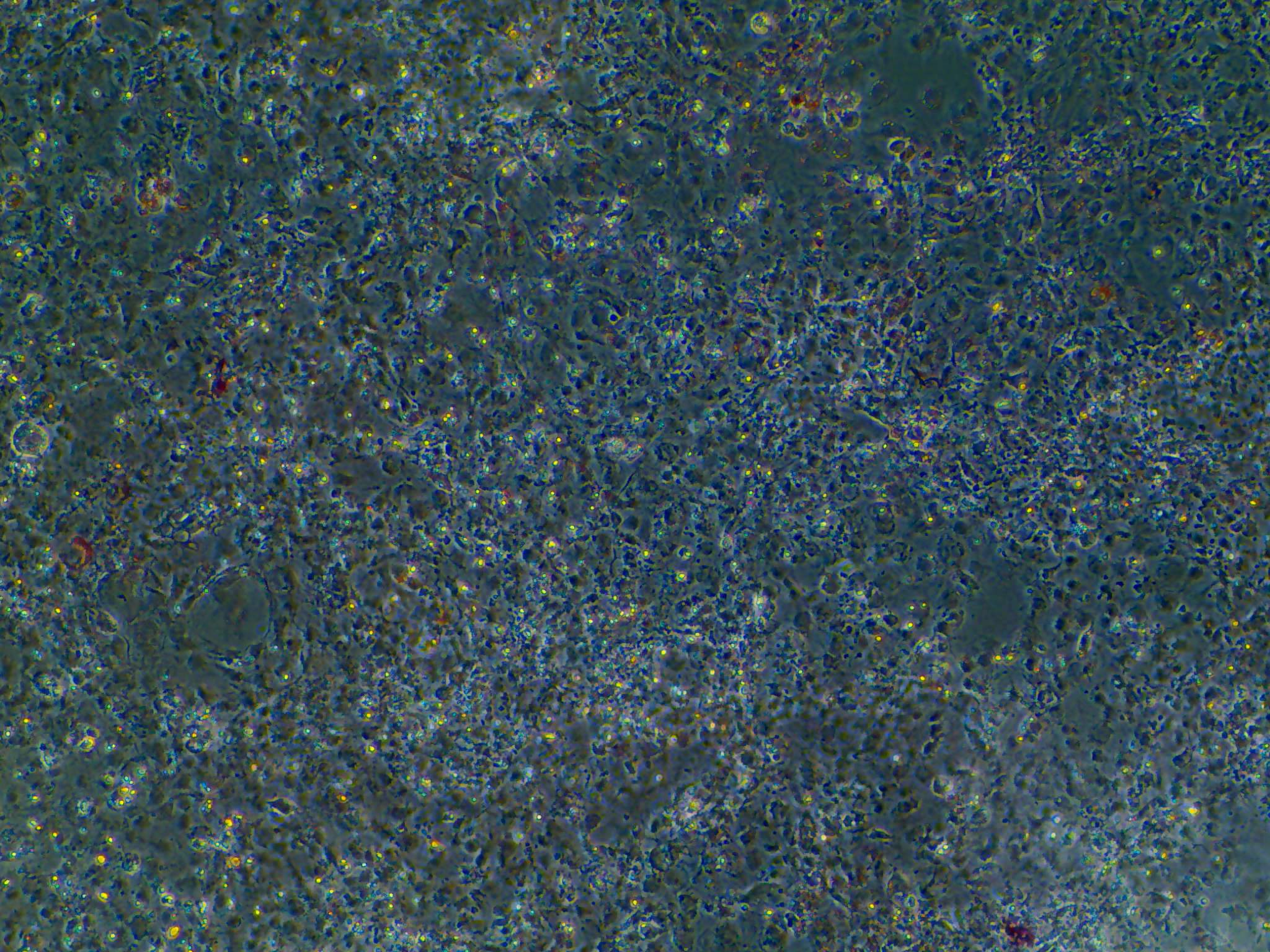

Supplement: Supplementary file 3 — Source data Fig. 1 [file 44318_2025_520_MOESM3_ESM.zip › Figure 1/1R/SC wt 10x Day 0.tif]

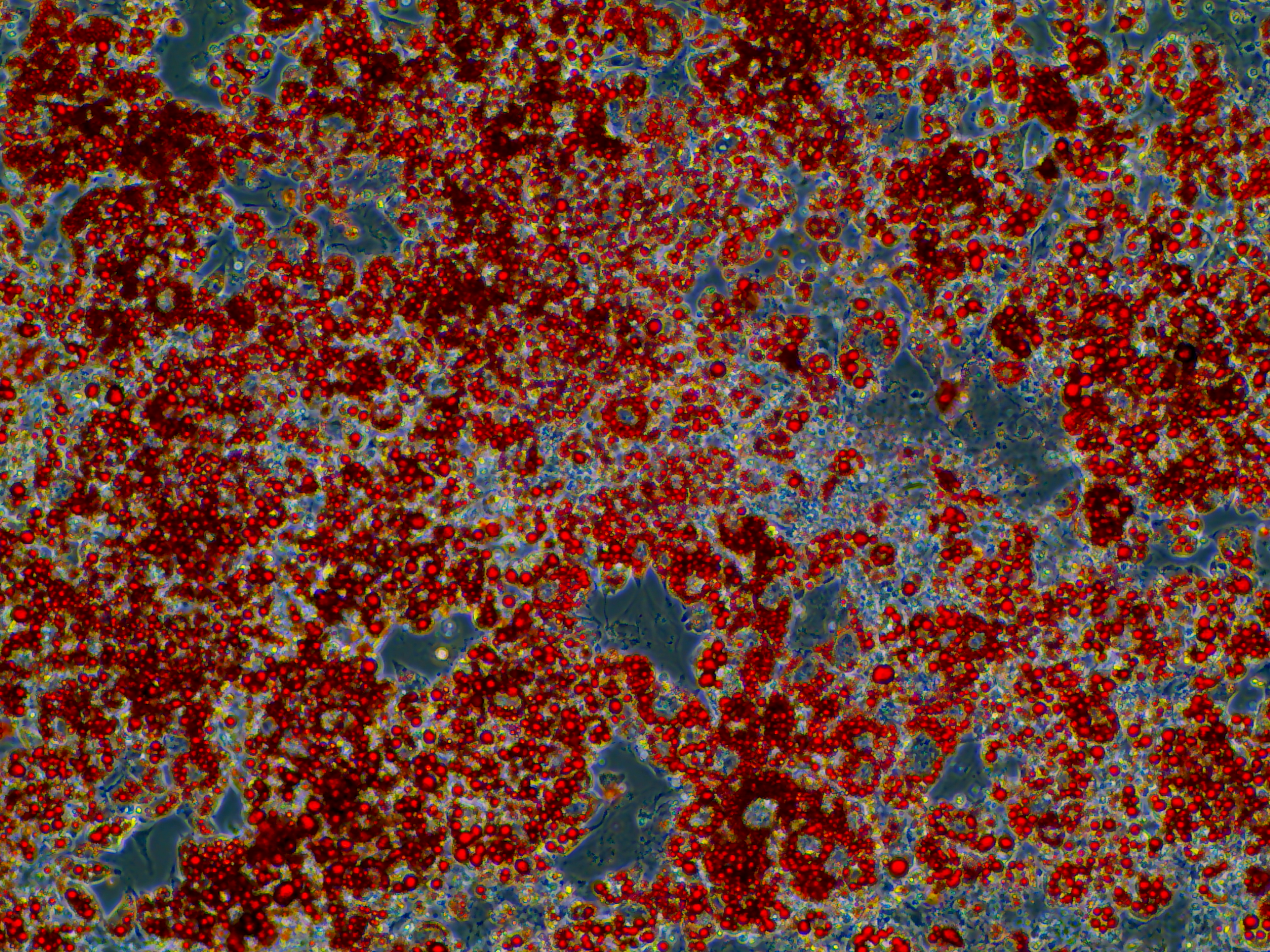

Supplement: Supplementary file 3 — Source data Fig. 1 [file 44318_2025_520_MOESM3_ESM.zip › Figure 1/1R/SC ko 10x Day 8.tif]

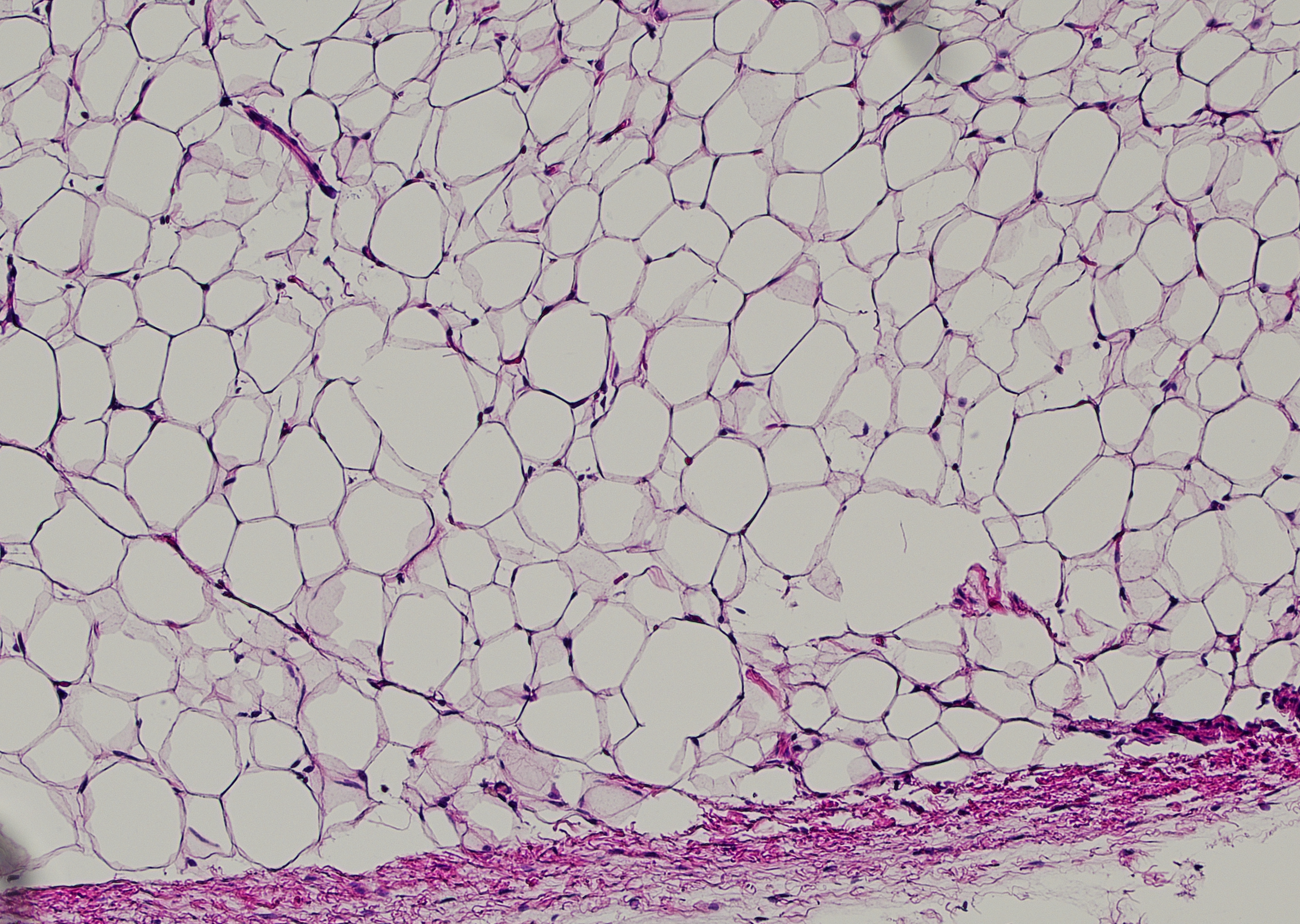

Supplement: Supplementary file 3 — Source data Fig. 1 [file 44318_2025_520_MOESM3_ESM.zip › Figure 1/1G/SCF KO 10X.tif]

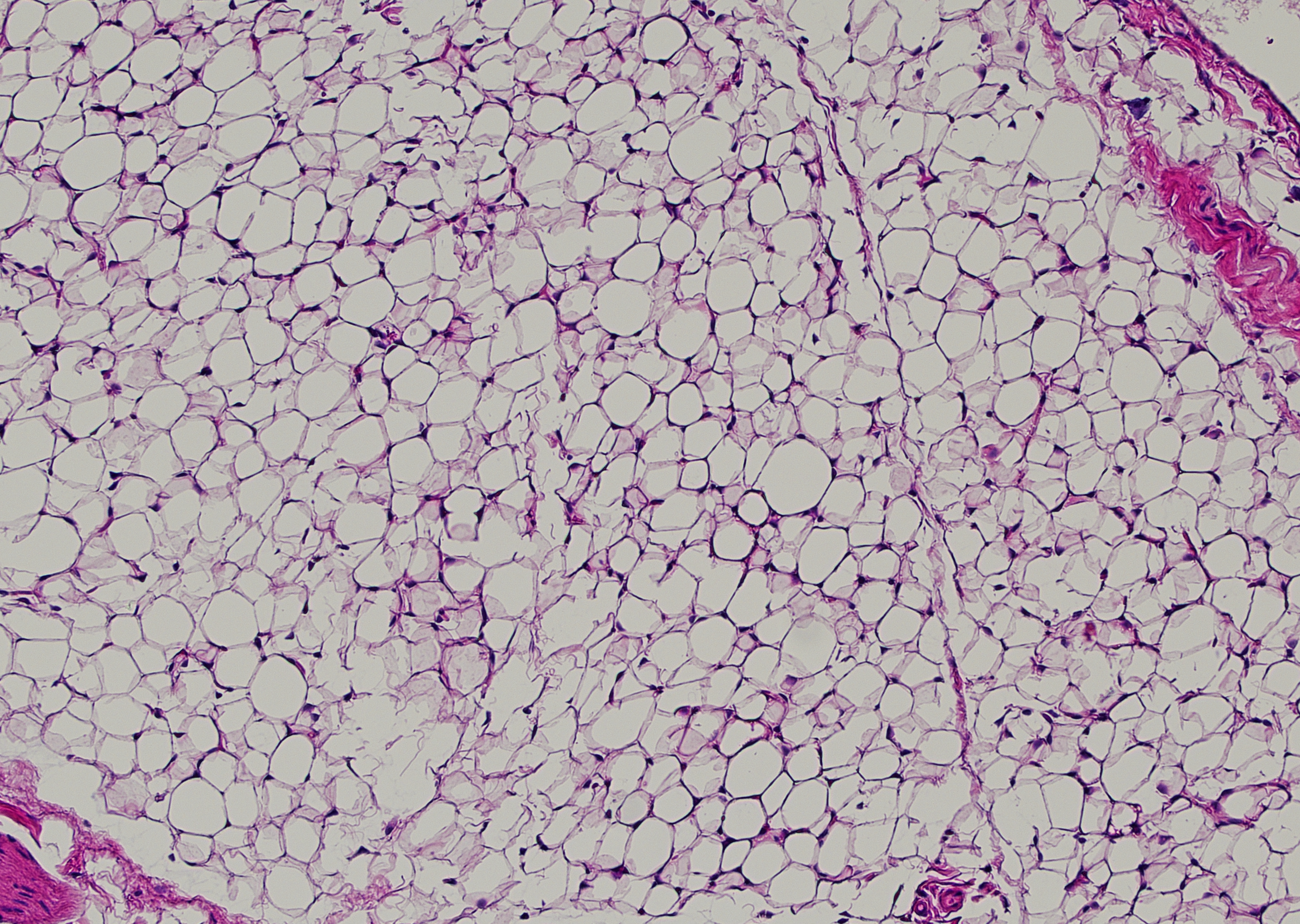

Supplement: Supplementary file 3 — Source data Fig. 1 [file 44318_2025_520_MOESM3_ESM.zip › Figure 1/1G/SCF WT 10X.tif]

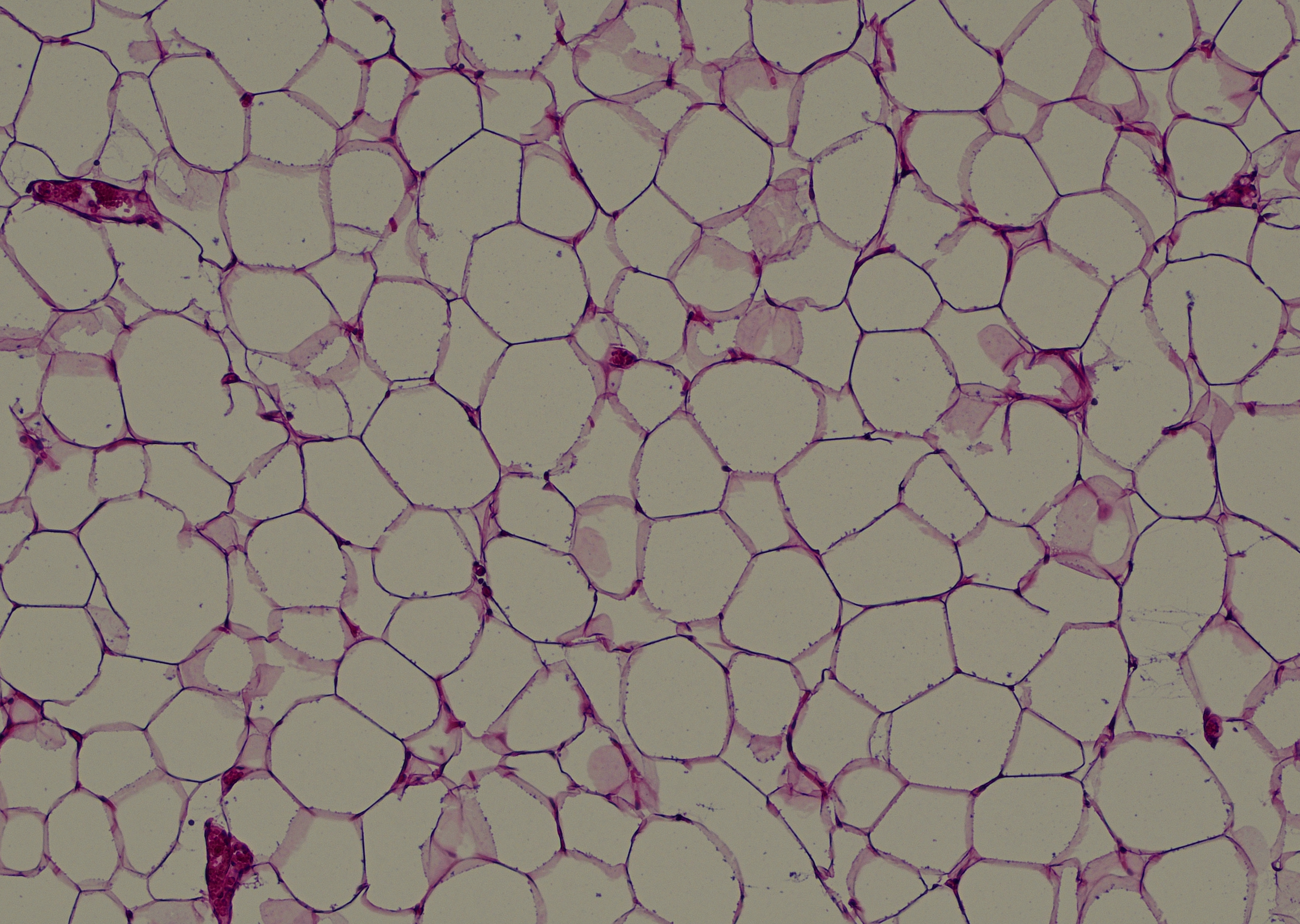

Supplement: Supplementary file 3 — Source data Fig. 1 [file 44318_2025_520_MOESM3_ESM.zip › Figure 1/1G/PGF KO 10x.tif]

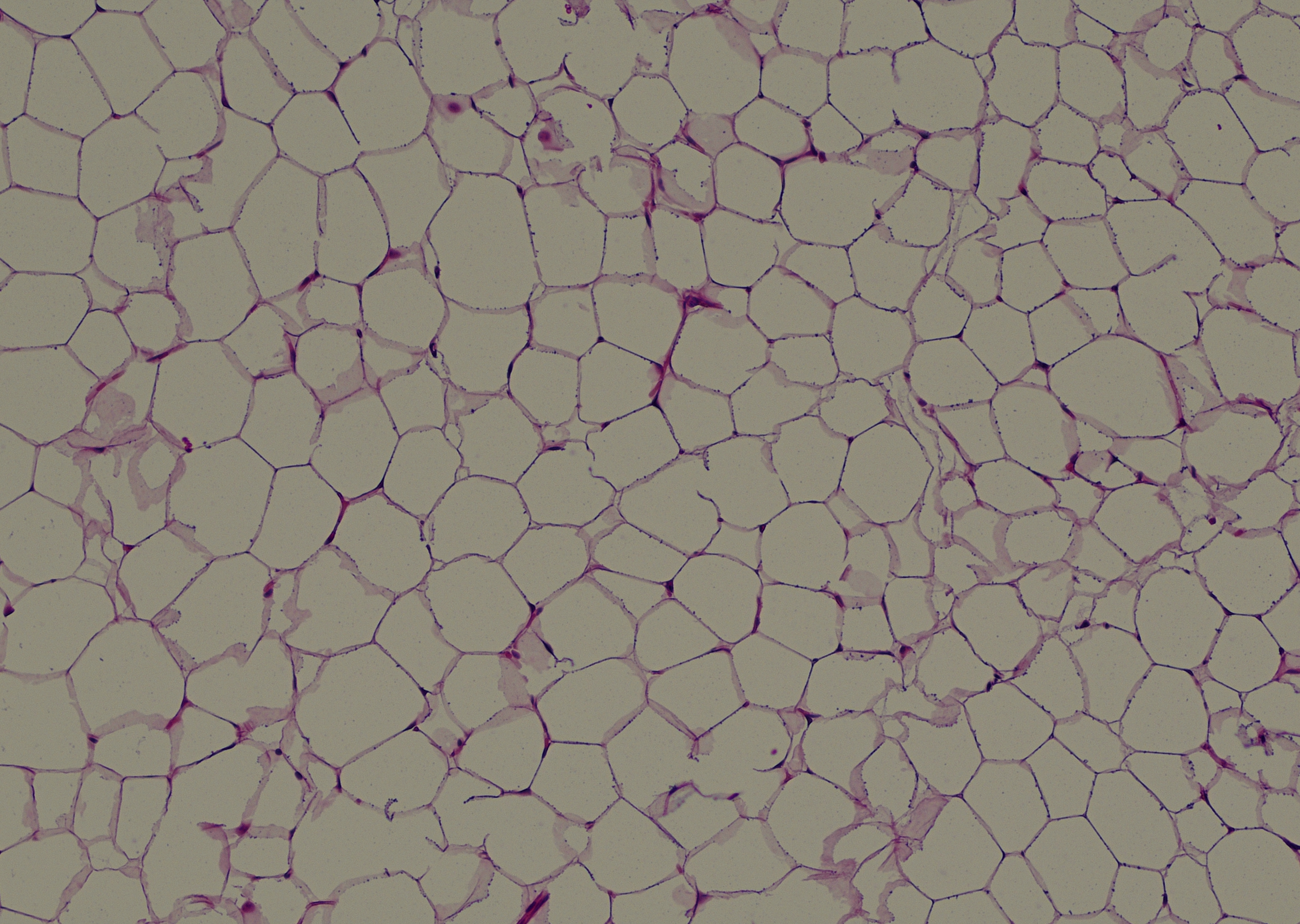

Supplement: Supplementary file 3 — Source data Fig. 1 [file 44318_2025_520_MOESM3_ESM.zip › Figure 1/1G/PGF WT 10x.tif]

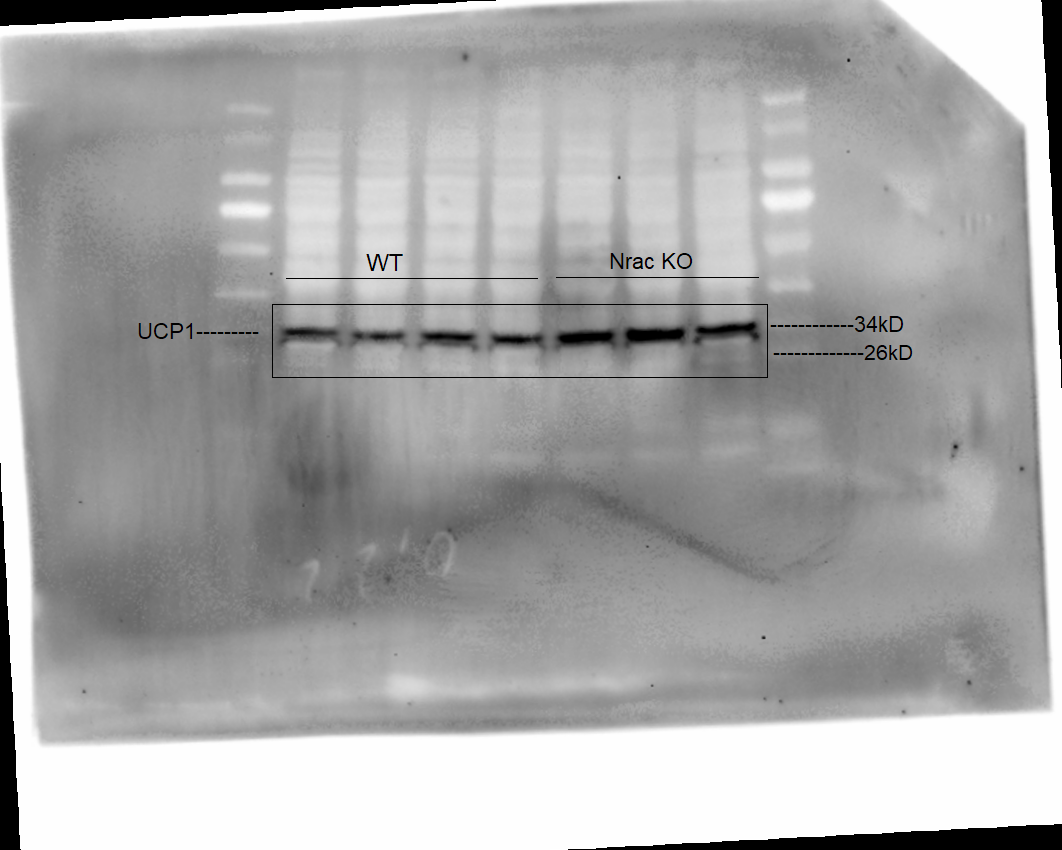

Supplement: Supplementary file 4 — Source data Fig. 2 [file 44318_2025_520_MOESM4_ESM.zip › Figure 2/2D/UCP1.tif]

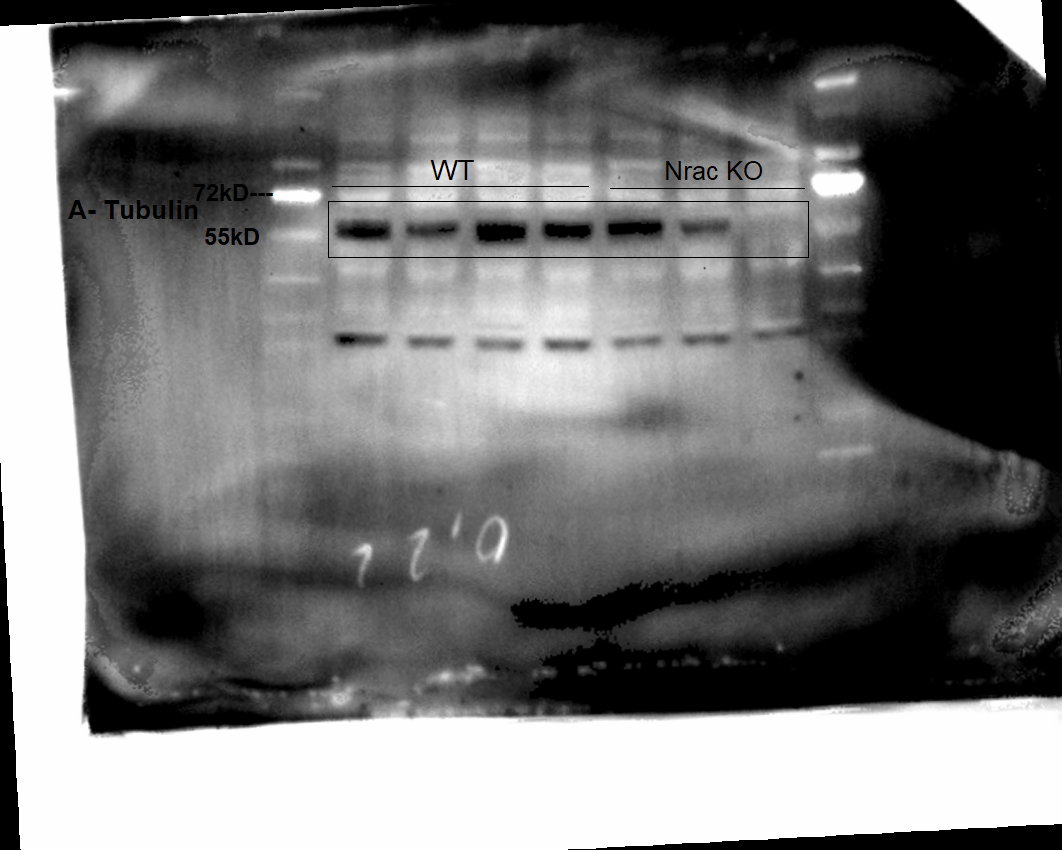

Supplement: Supplementary file 4 — Source data Fig. 2 [file 44318_2025_520_MOESM4_ESM.zip › Figure 2/2D/A- Tubulin.tif]

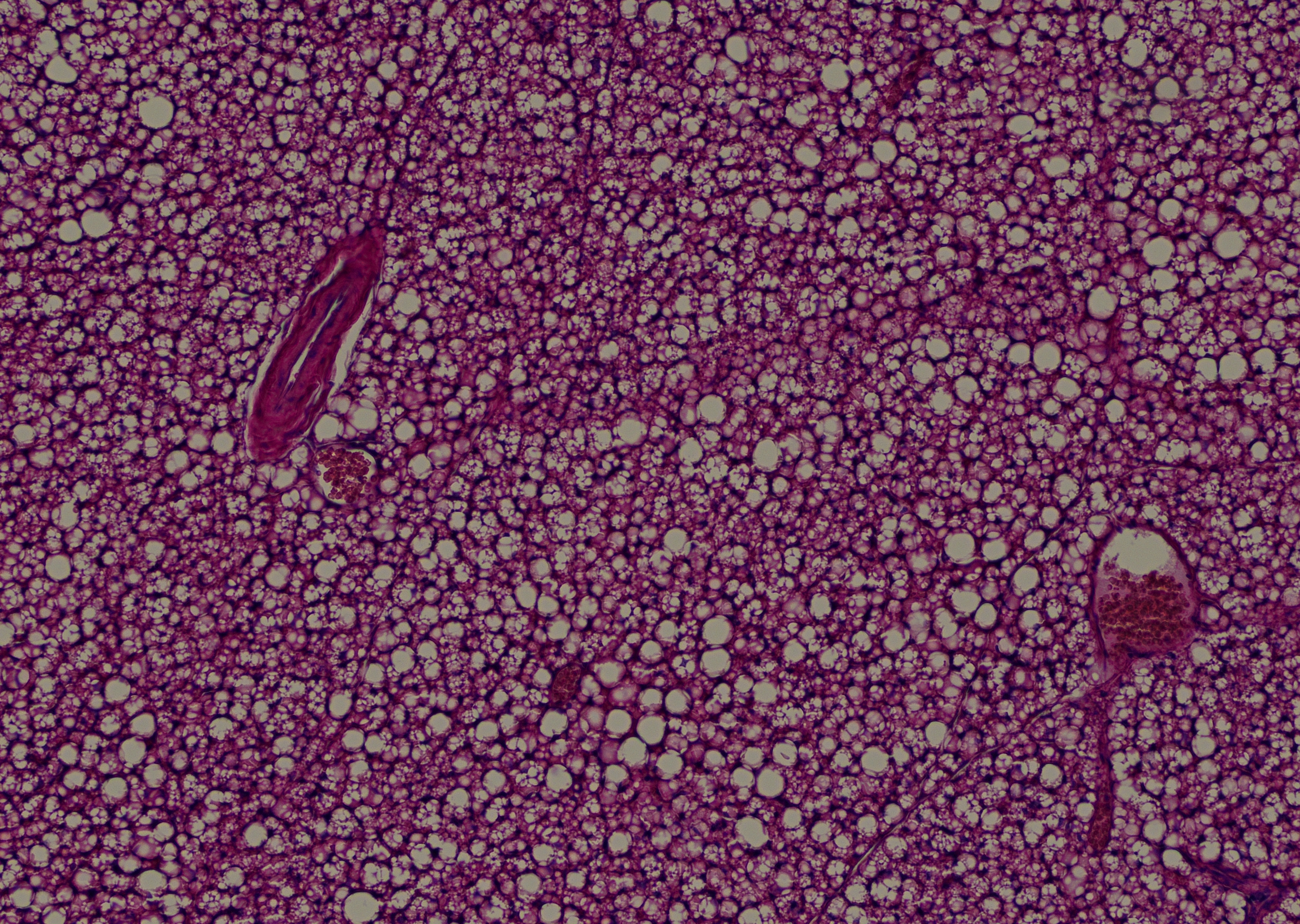

Supplement: Supplementary file 4 — Source data Fig. 2 [file 44318_2025_520_MOESM4_ESM.zip › Figure 2/2E/BAT KO 10x.tif]

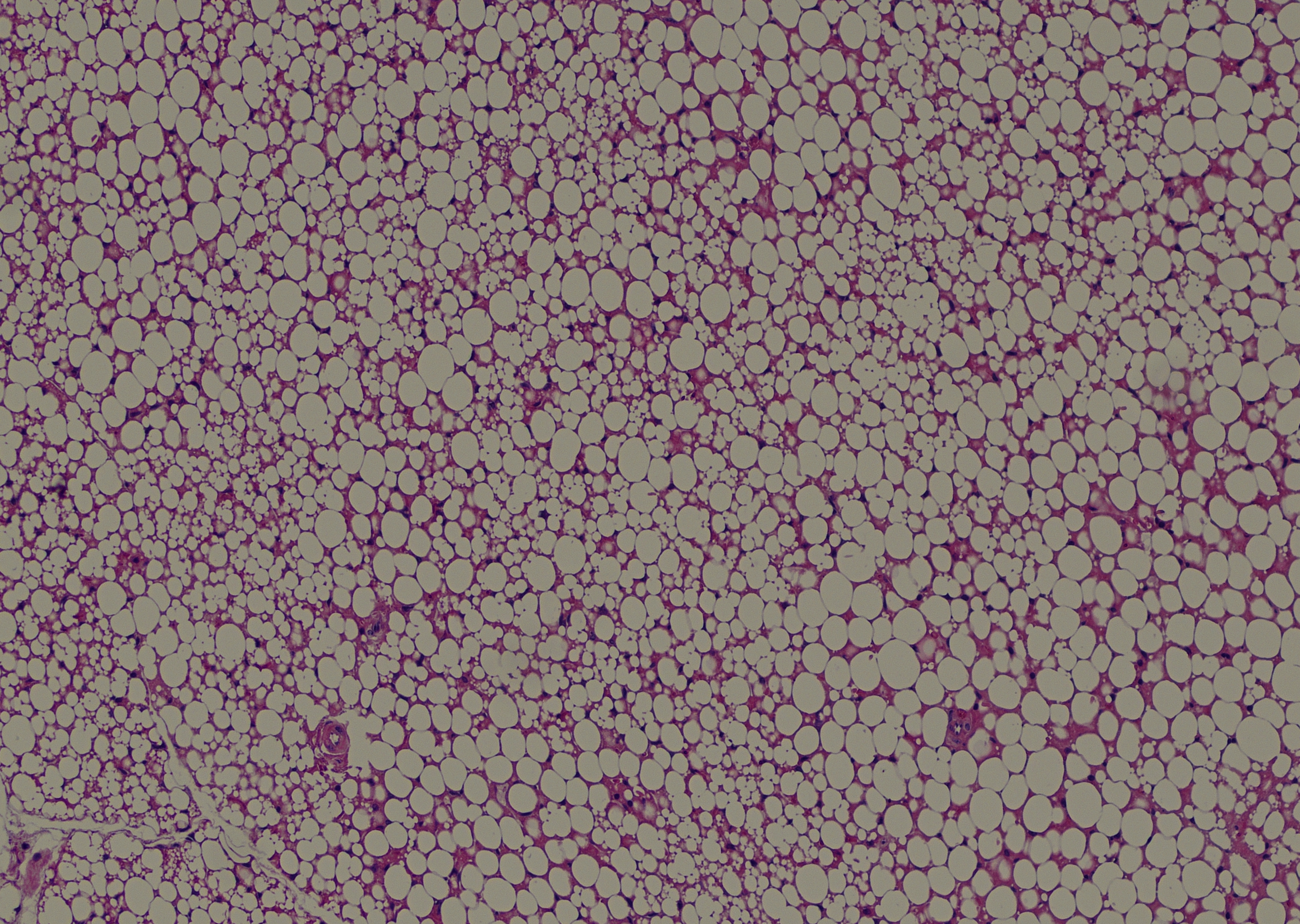

Supplement: Supplementary file 4 — Source data Fig. 2 [file 44318_2025_520_MOESM4_ESM.zip › Figure 2/2E/BAT WT 10x.tif]

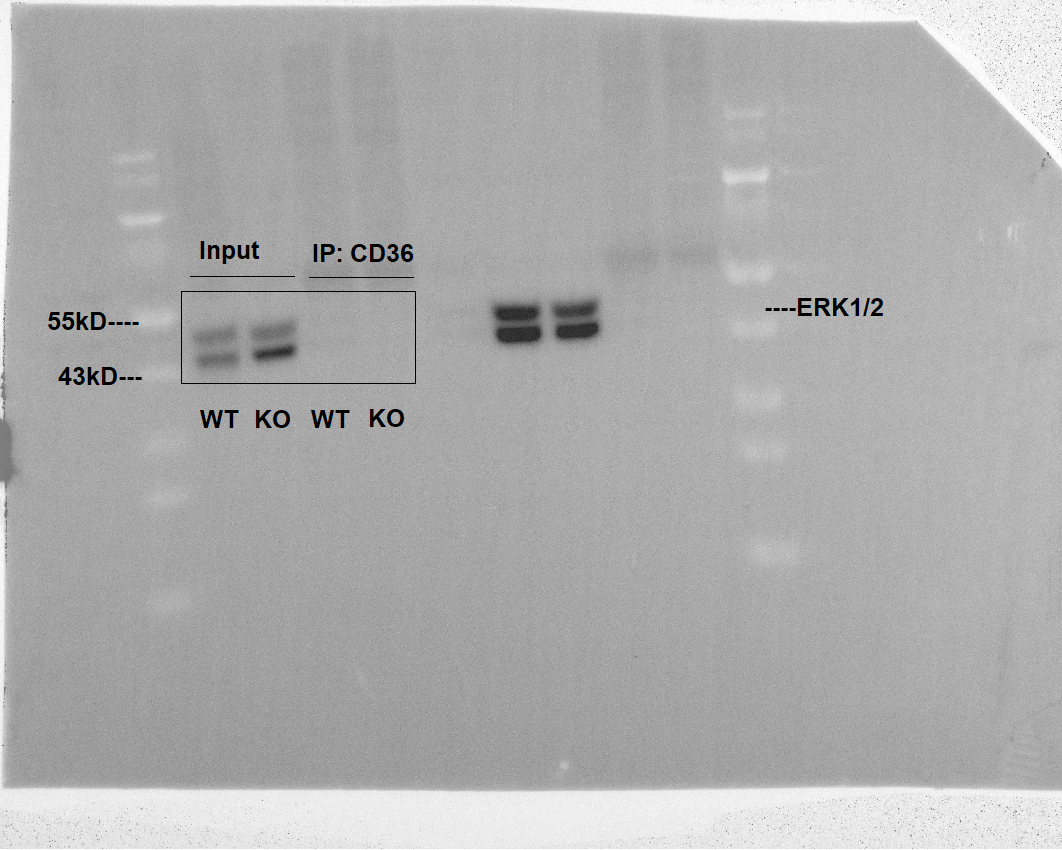

Supplement: Supplementary file 5 — Source data Fig. 3 [file 44318_2025_520_MOESM5_ESM.zip › Figure 3/3E/ERK1-2.tif]

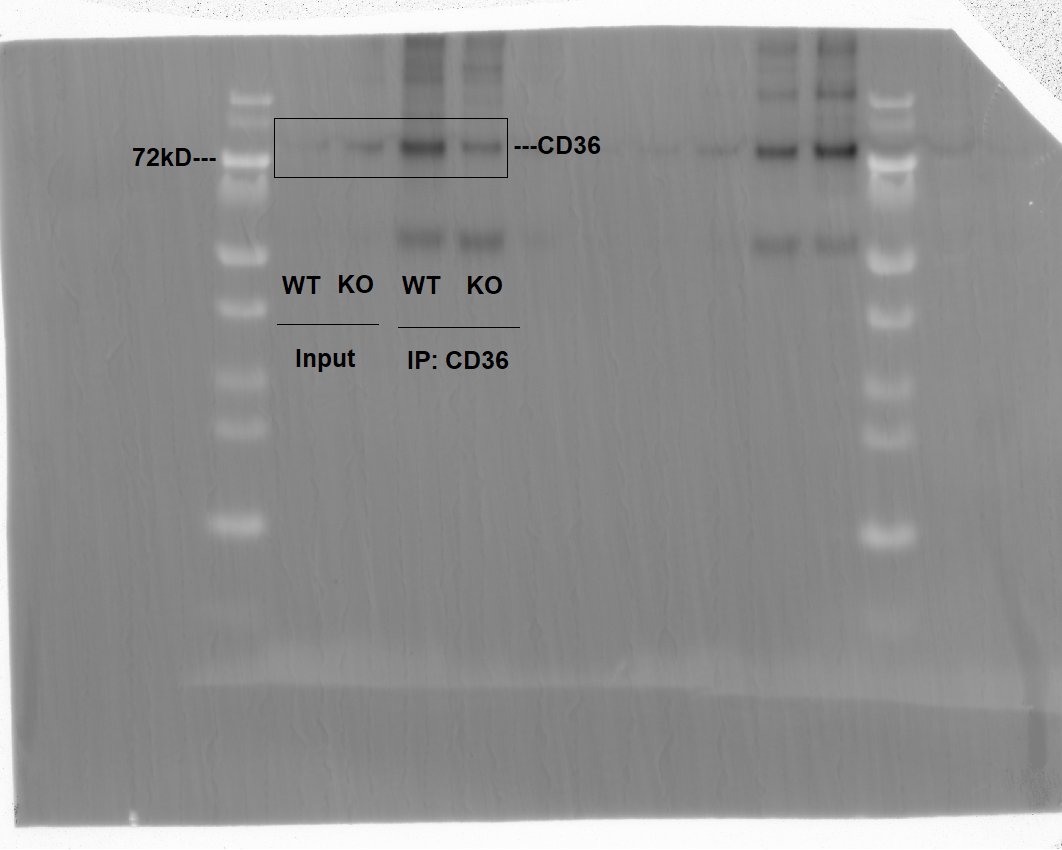

Supplement: Supplementary file 5 — Source data Fig. 3 [file 44318_2025_520_MOESM5_ESM.zip › Figure 3/3E/CD36.tif]

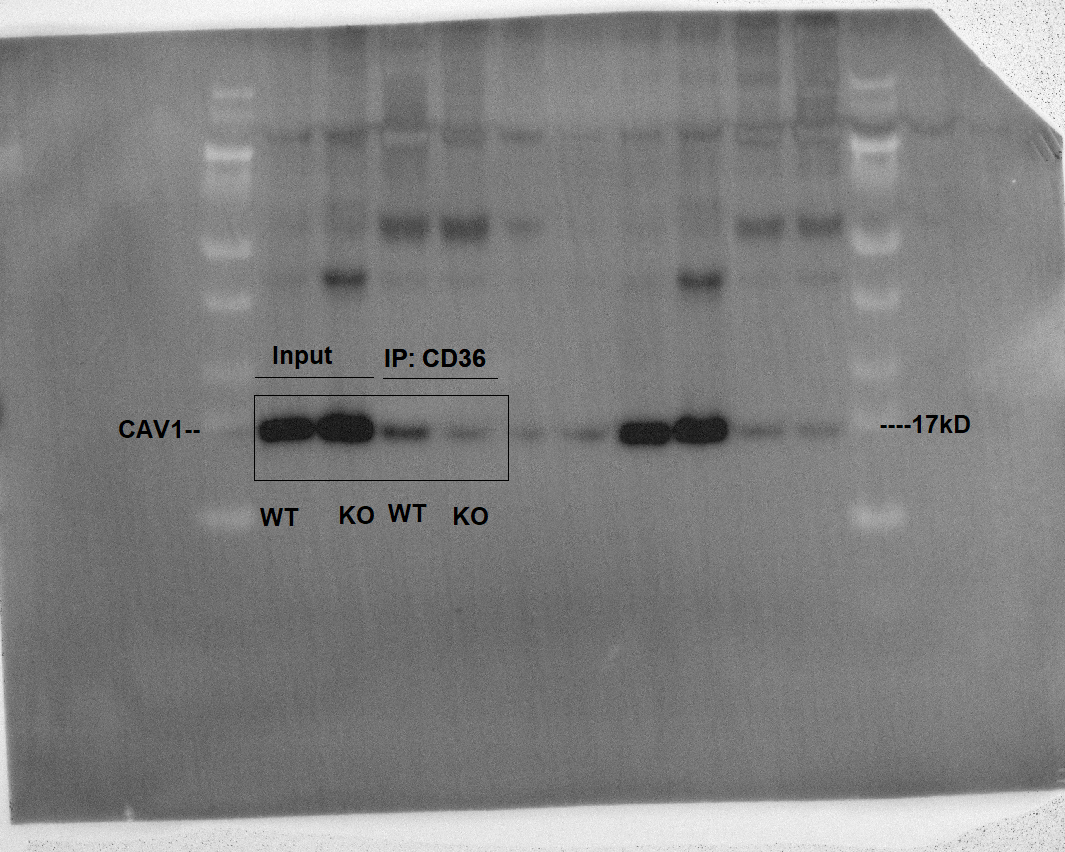

Supplement: Supplementary file 5 — Source data Fig. 3 [file 44318_2025_520_MOESM5_ESM.zip › Figure 3/3E/CAV1.tif]

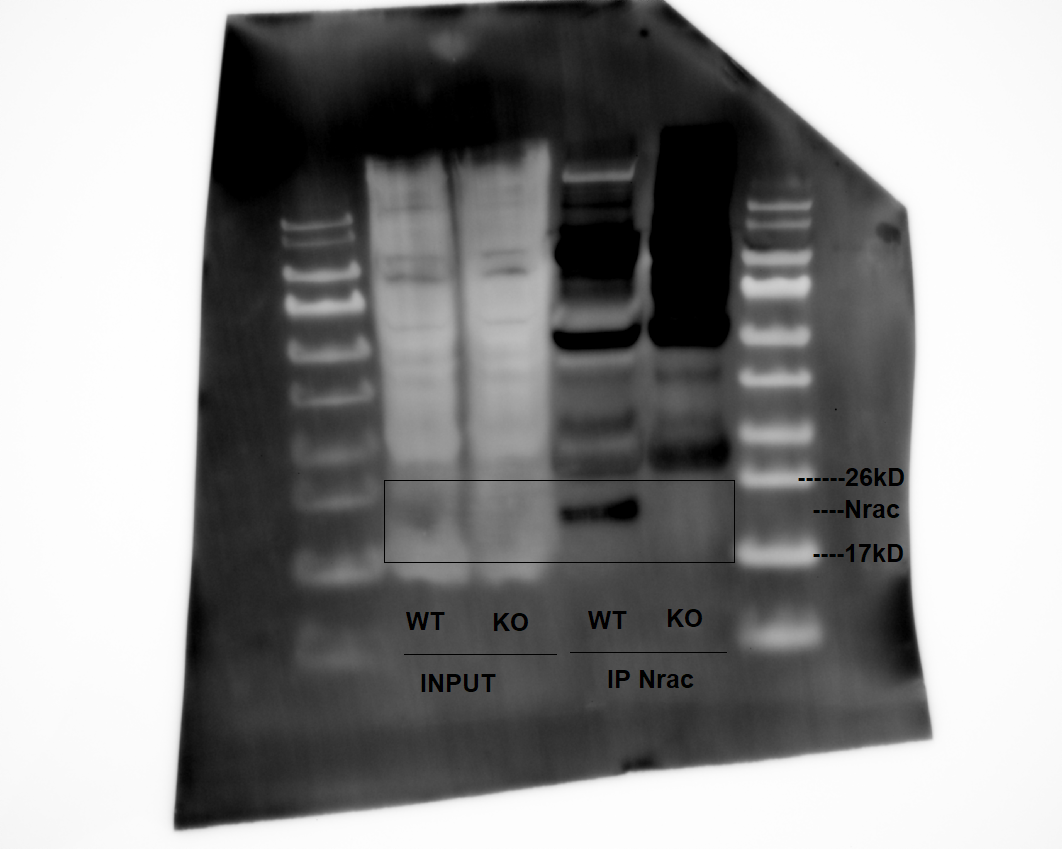

Supplement: Supplementary file 5 — Source data Fig. 3 [file 44318_2025_520_MOESM5_ESM.zip › Figure 3/3B/Nrac.tif]

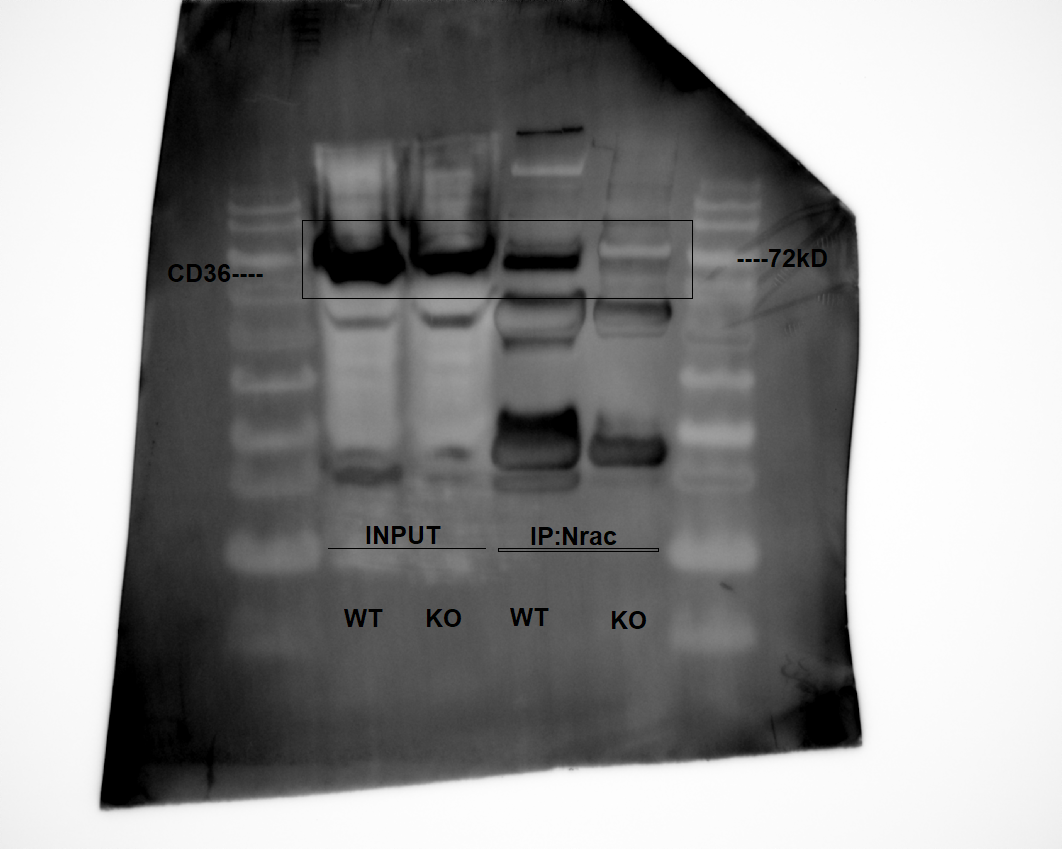

Supplement: Supplementary file 5 — Source data Fig. 3 [file 44318_2025_520_MOESM5_ESM.zip › Figure 3/3B/CD36.tif]

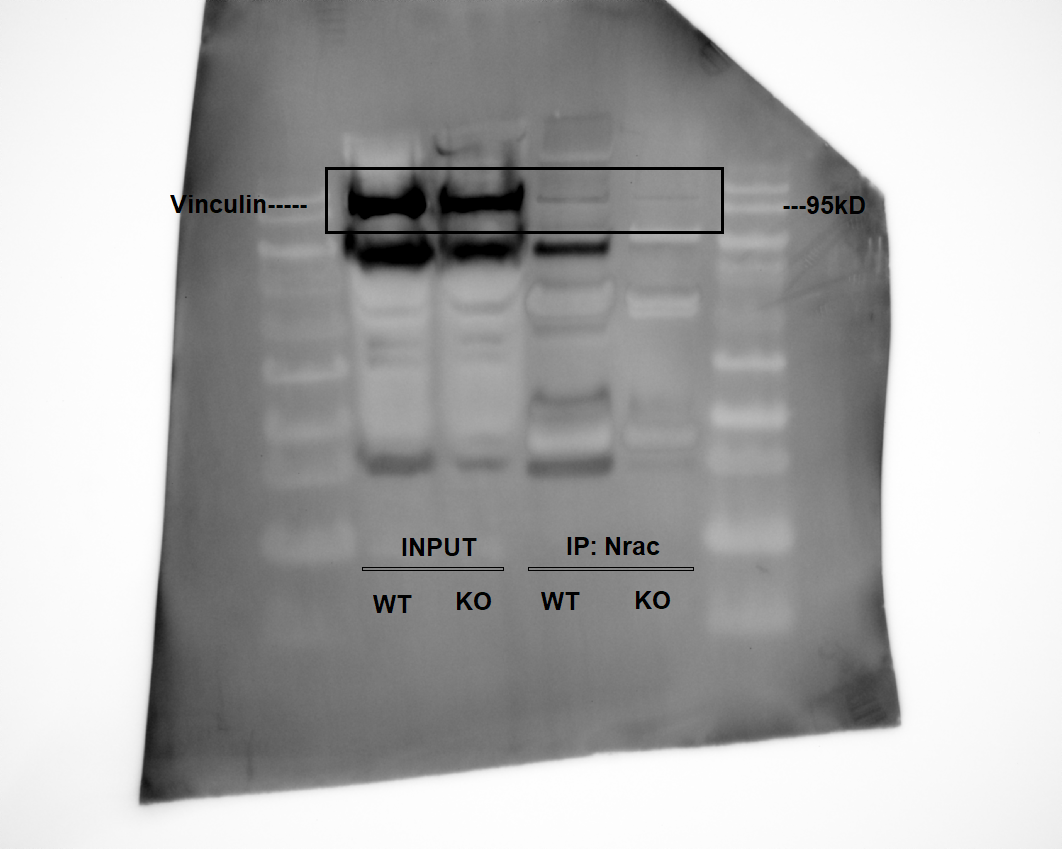

Supplement: Supplementary file 5 — Source data Fig. 3 [file 44318_2025_520_MOESM5_ESM.zip › Figure 3/3B/Vinculin.tif]

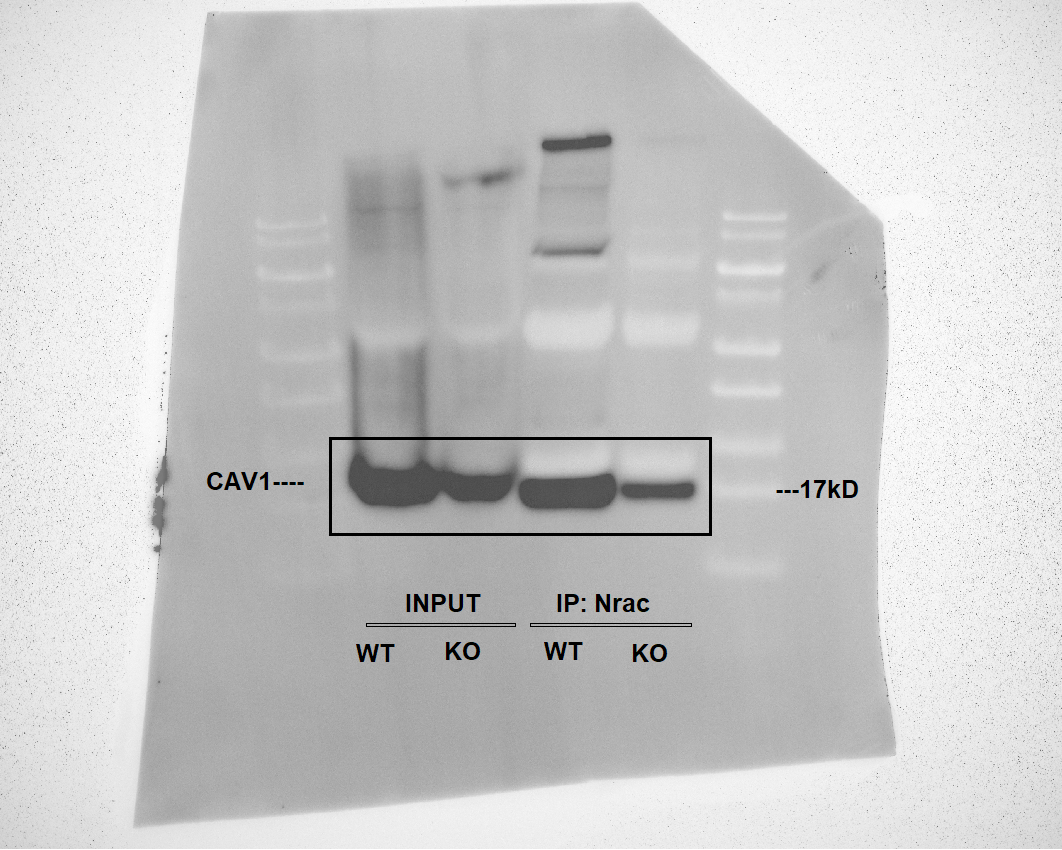

Supplement: Supplementary file 5 — Source data Fig. 3 [file 44318_2025_520_MOESM5_ESM.zip › Figure 3/3B/CAV1.tif]

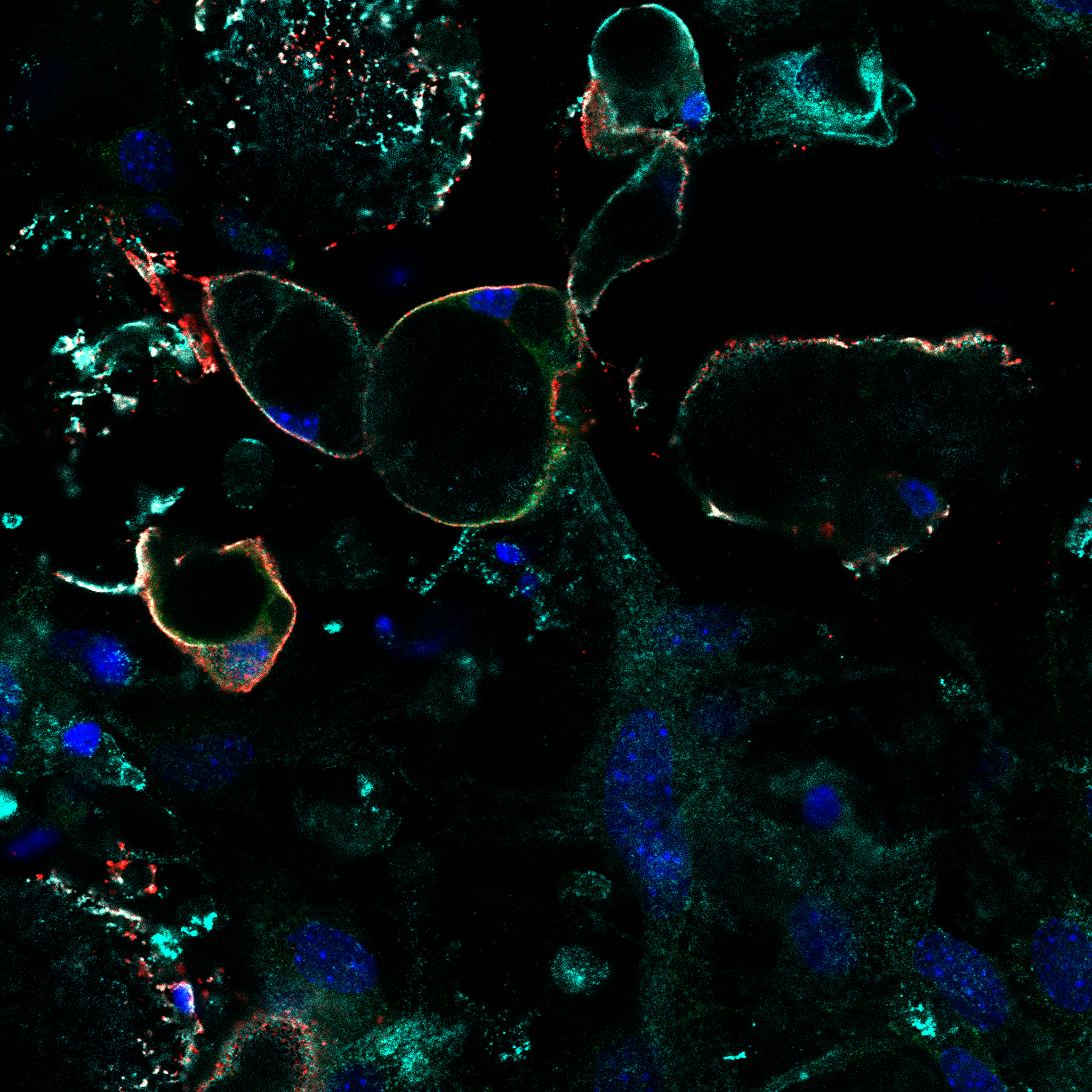

Supplement: Supplementary file 5 — Source data Fig. 3 [file 44318_2025_520_MOESM5_ESM.zip › Figure 3/3C/WT-vehicle.tif]

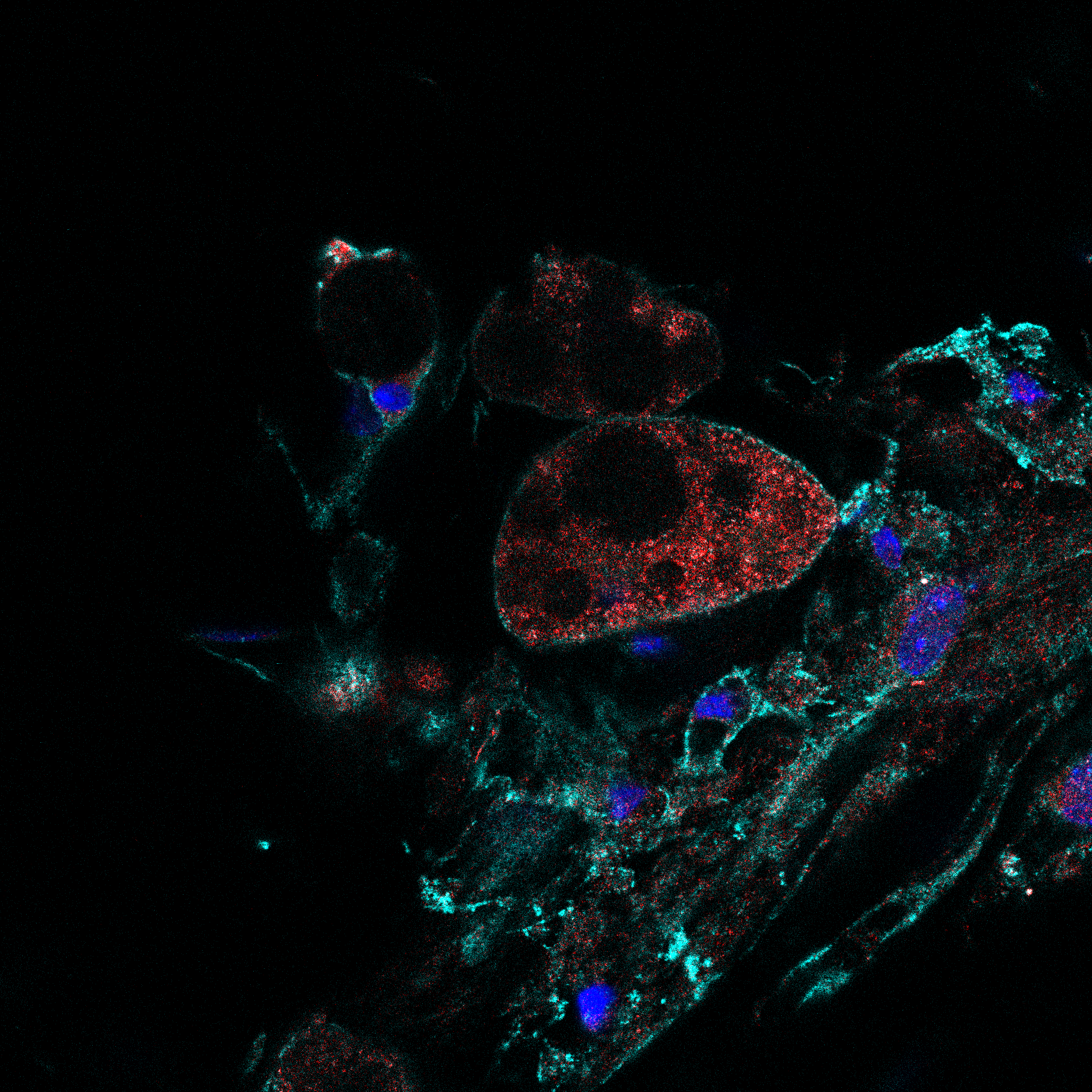

Supplement: Supplementary file 5 — Source data Fig. 3 [file 44318_2025_520_MOESM5_ESM.zip › Figure 3/3C/KO- vehicle.tif]

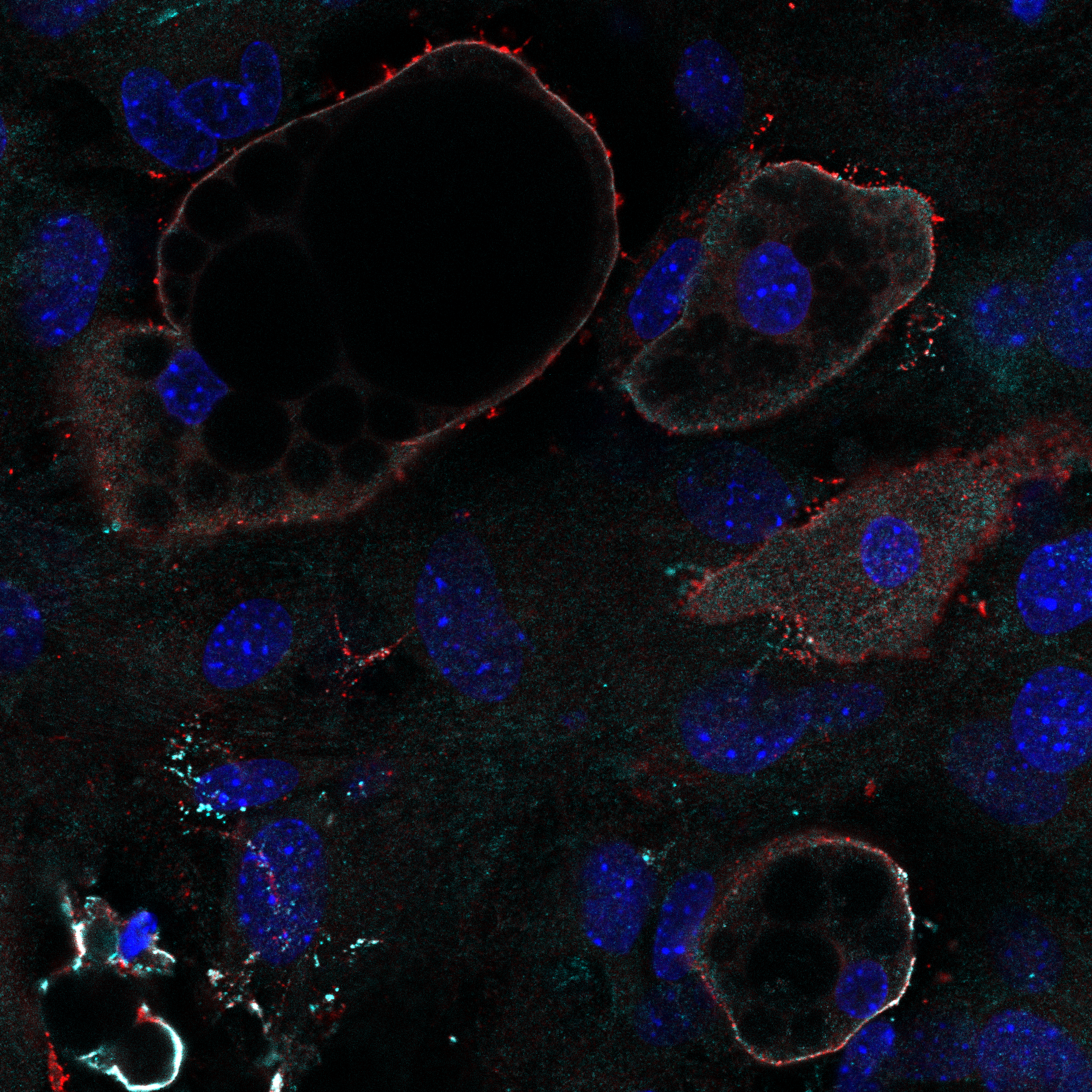

Supplement: Supplementary file 5 — Source data Fig. 3 [file 44318_2025_520_MOESM5_ESM.zip › Figure 3/3C/KO-CPZ.tif]

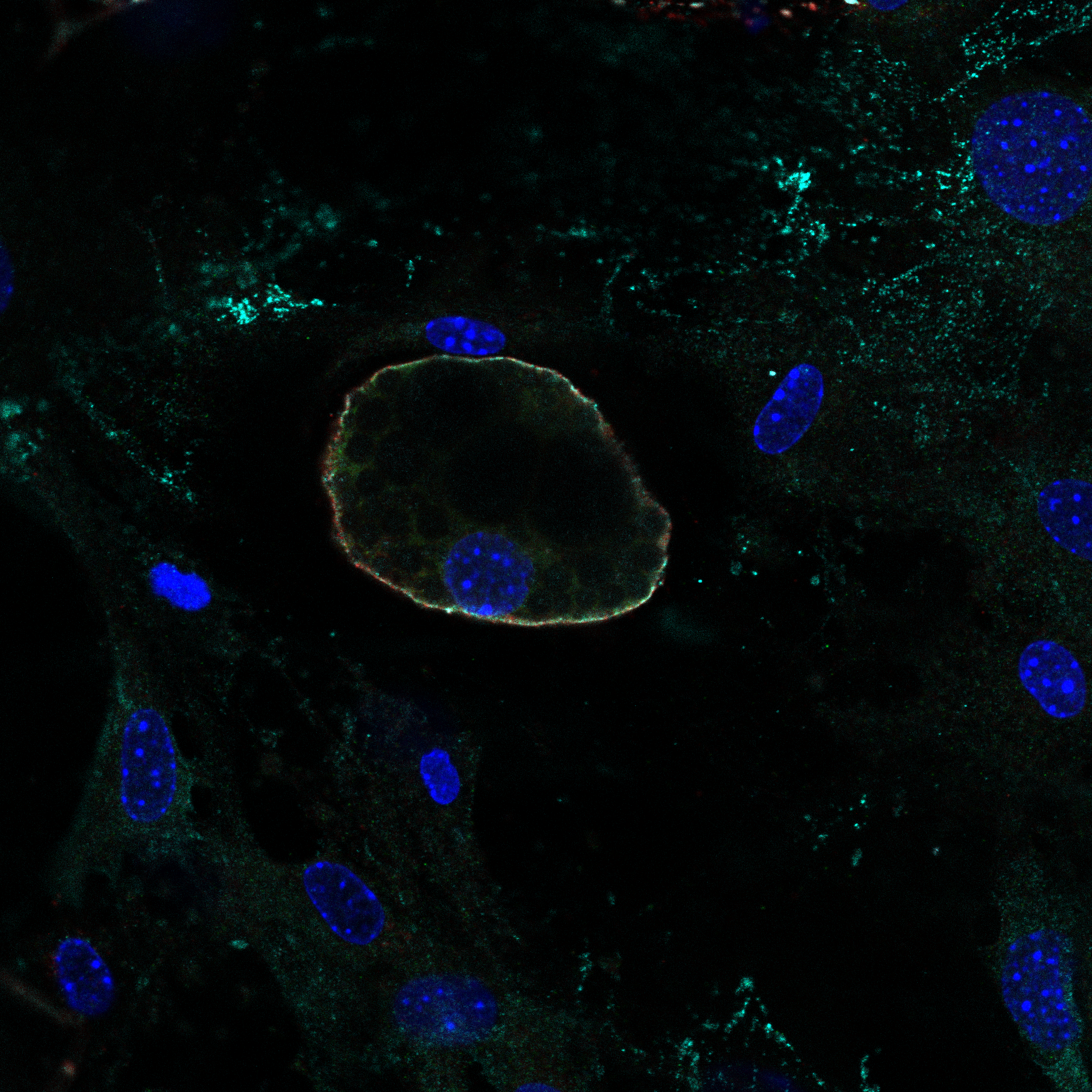

Supplement: Supplementary file 5 — Source data Fig. 3 [file 44318_2025_520_MOESM5_ESM.zip › Figure 3/3C/WT-CPZ.tif]

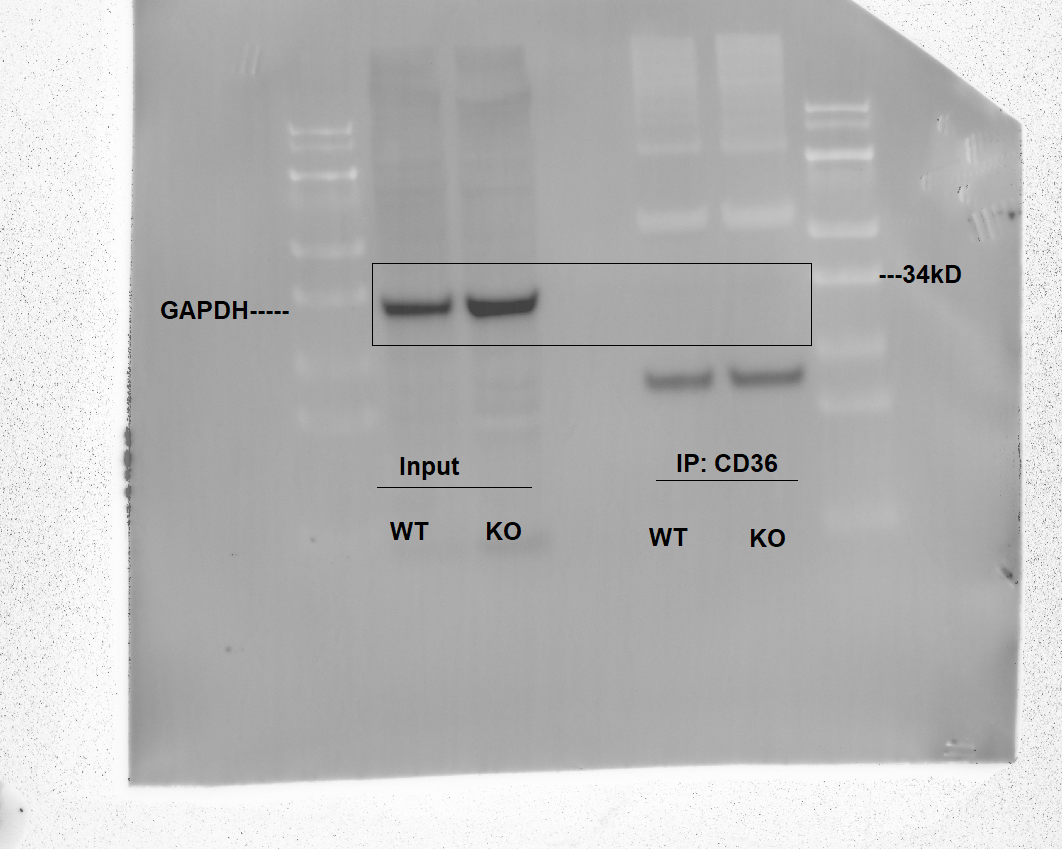

Supplement: Supplementary file 5 — Source data Fig. 3 [file 44318_2025_520_MOESM5_ESM.zip › Figure 3/3D/GAPDH.tif]

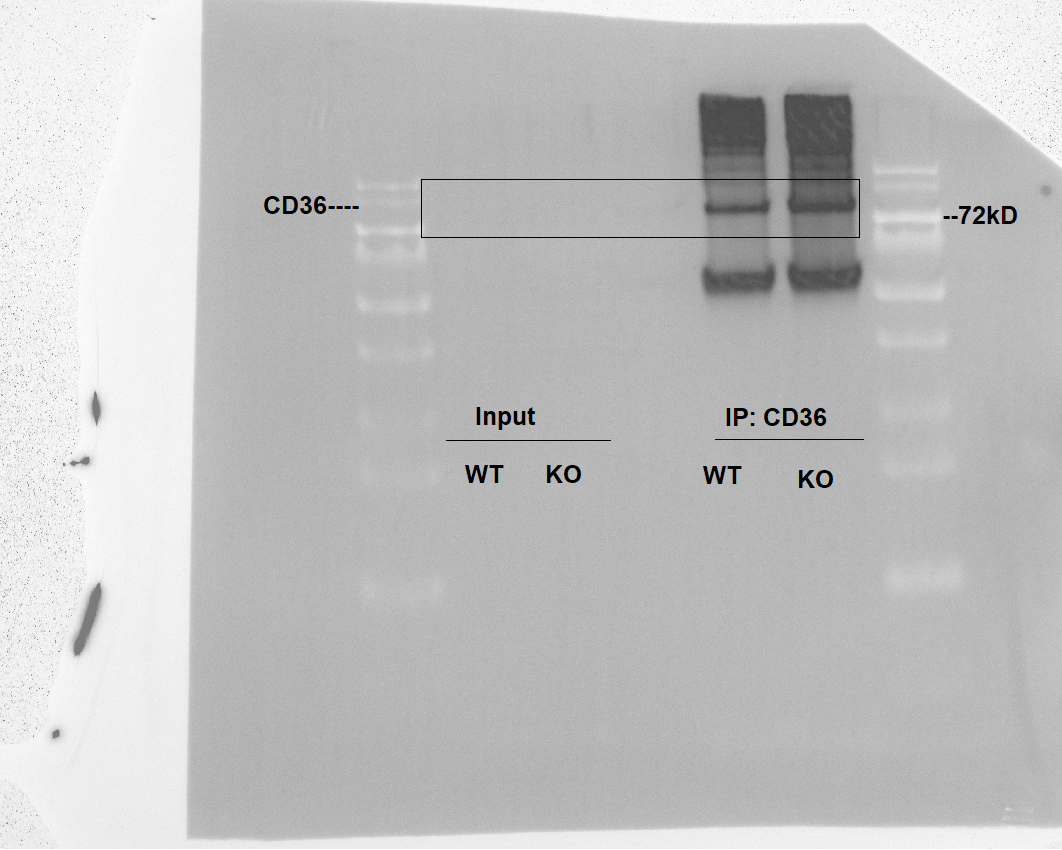

Supplement: Supplementary file 5 — Source data Fig. 3 [file 44318_2025_520_MOESM5_ESM.zip › Figure 3/3D/CD36.tif]

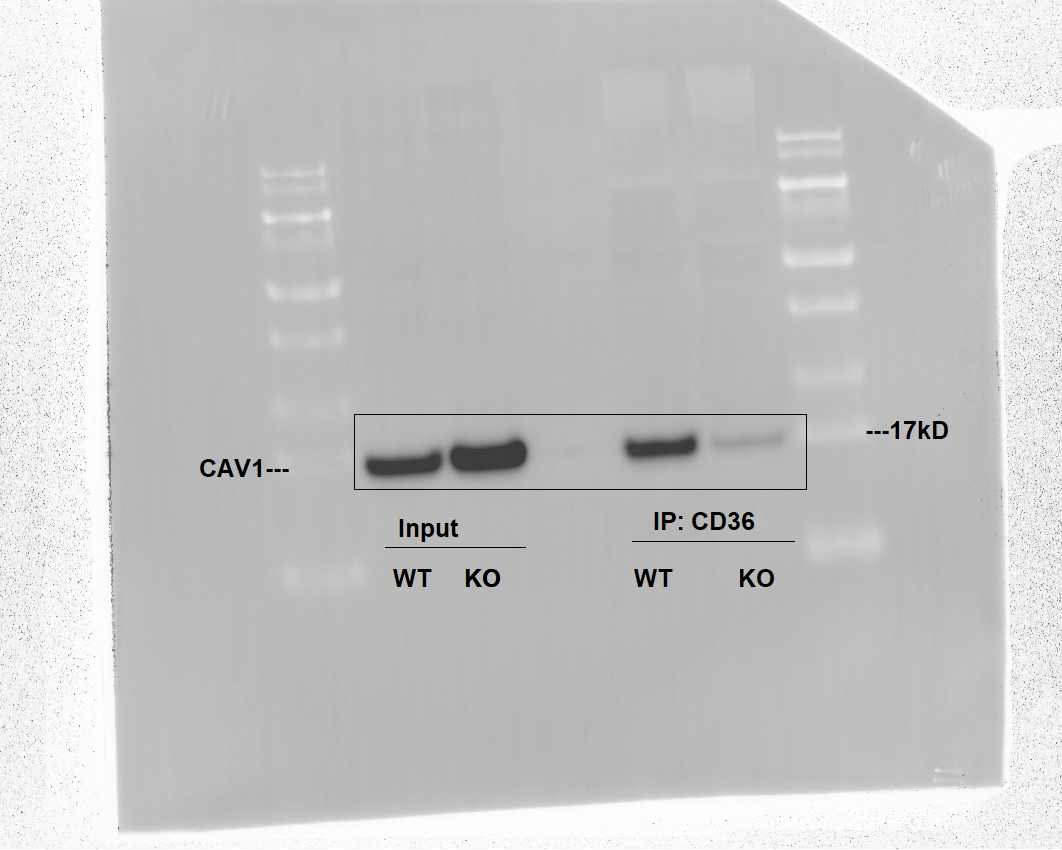

Supplement: Supplementary file 5 — Source data Fig. 3 [file 44318_2025_520_MOESM5_ESM.zip › Figure 3/3D/CAV1.tif]

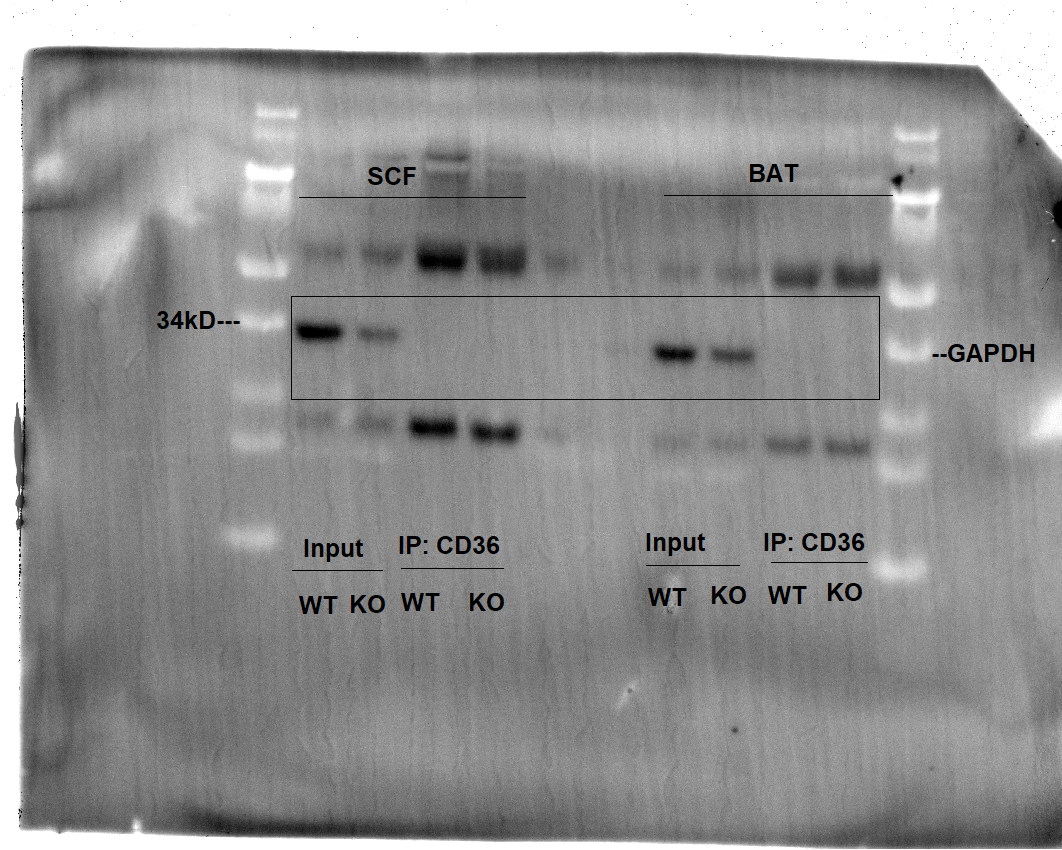

Supplement: Supplementary file 5 — Source data Fig. 3 [file 44318_2025_520_MOESM5_ESM.zip › Figure 3/3F and 3G/GAPDH.tif]

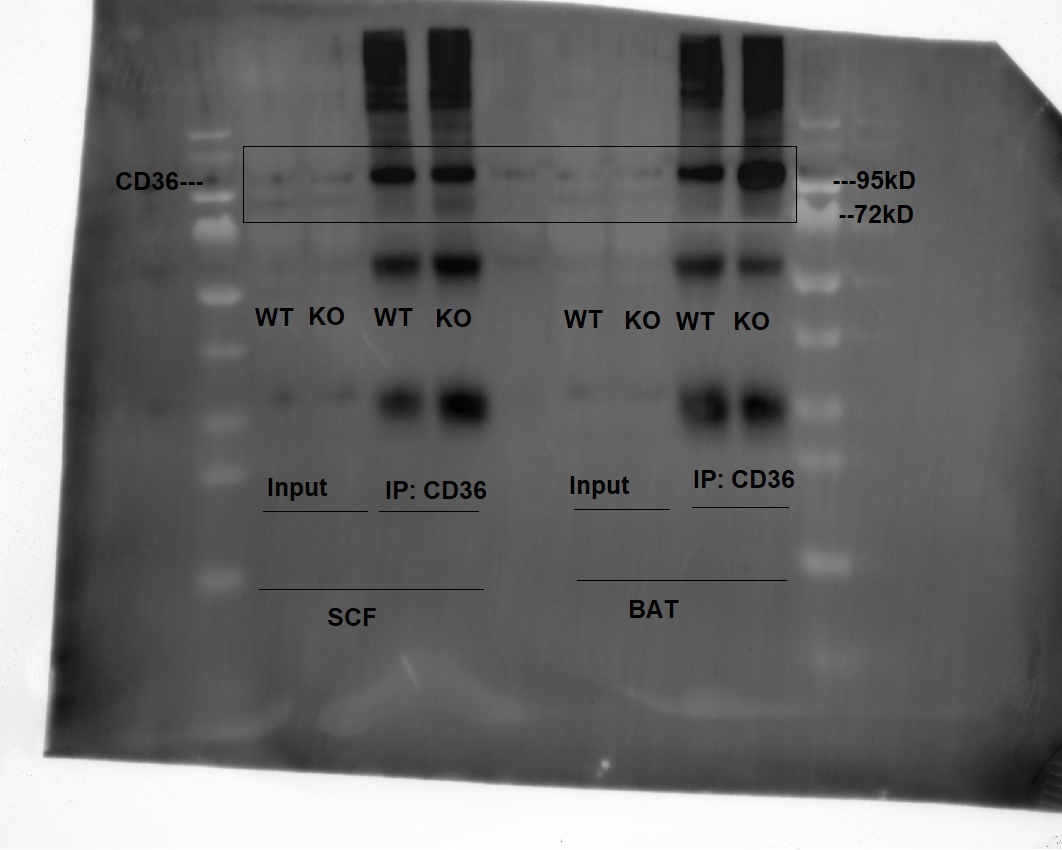

Supplement: Supplementary file 5 — Source data Fig. 3 [file 44318_2025_520_MOESM5_ESM.zip › Figure 3/3F and 3G/CD36.tif]

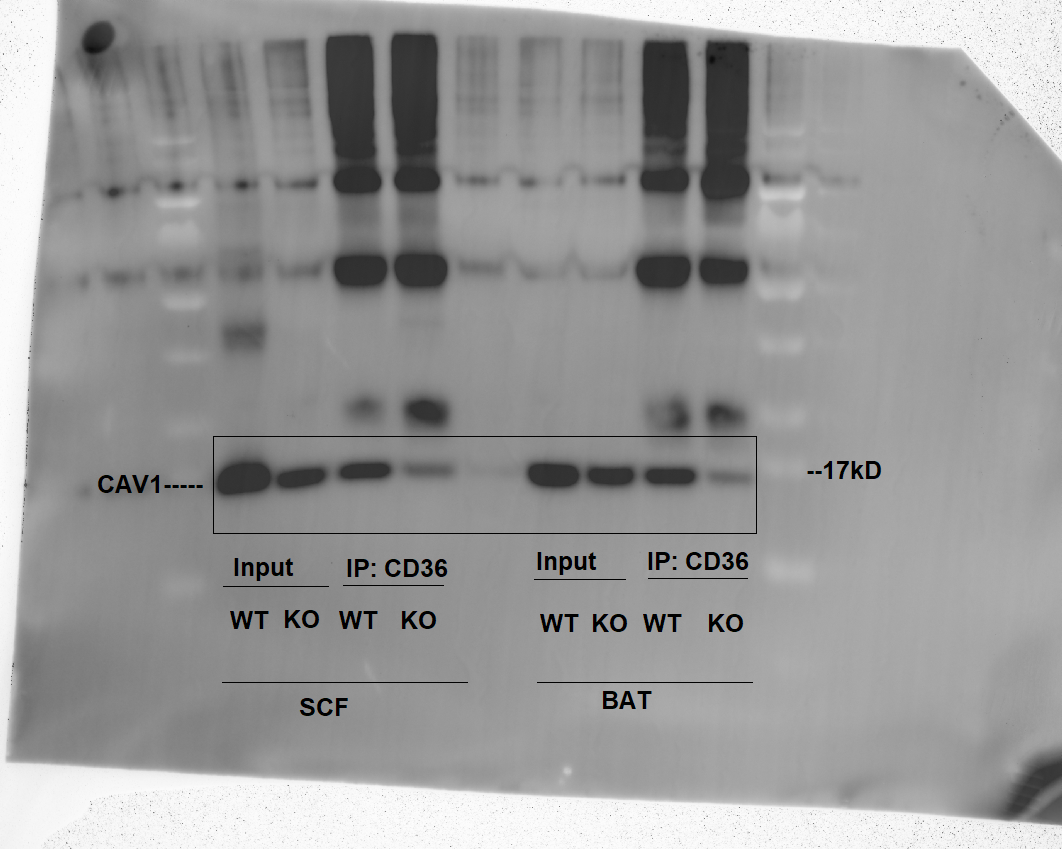

Supplement: Supplementary file 5 — Source data Fig. 3 [file 44318_2025_520_MOESM5_ESM.zip › Figure 3/3F and 3G/CAV1.tif]

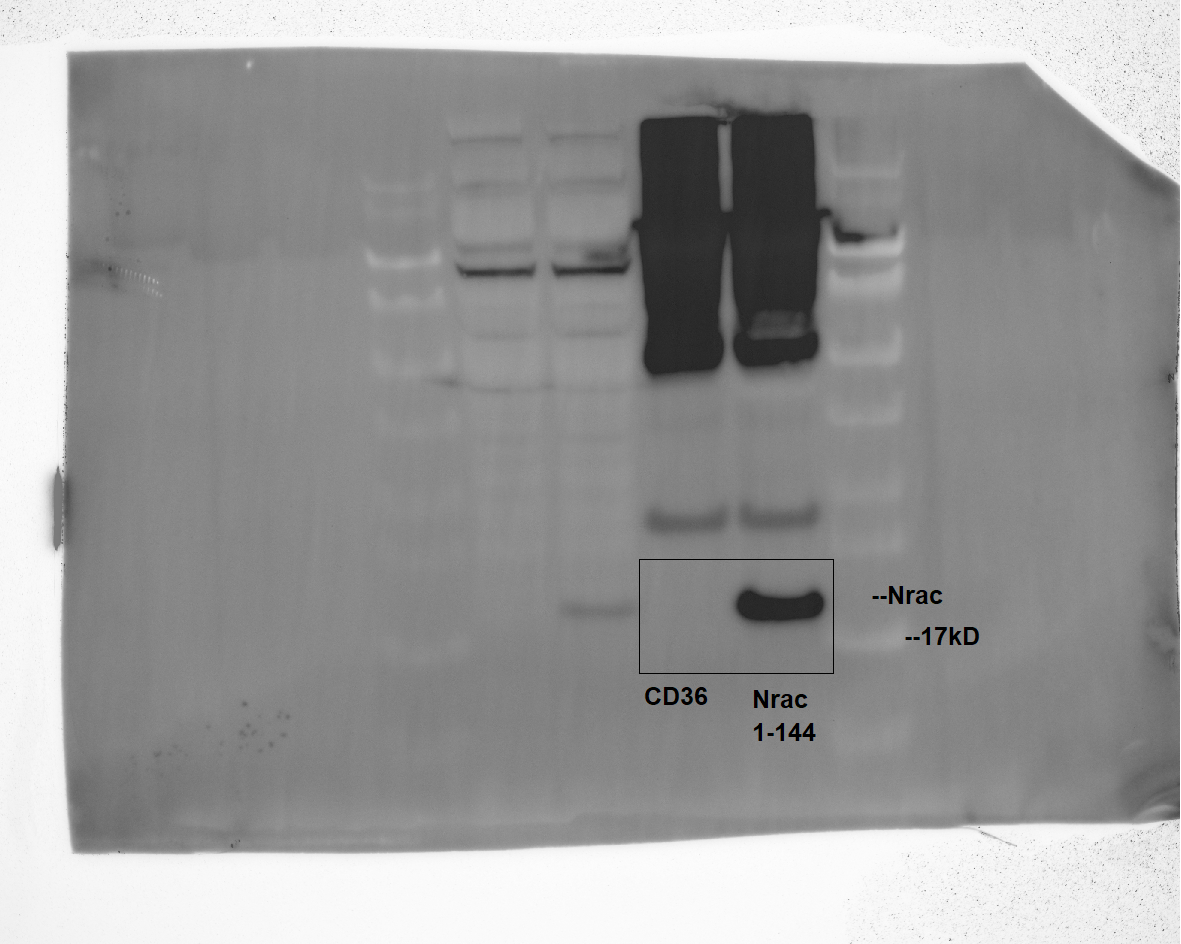

Supplement: Supplementary file 6 — Source data Fig. 4 [file 44318_2025_520_MOESM6_ESM.zip › Figure 4/4B/Nrac_1-144.tif]

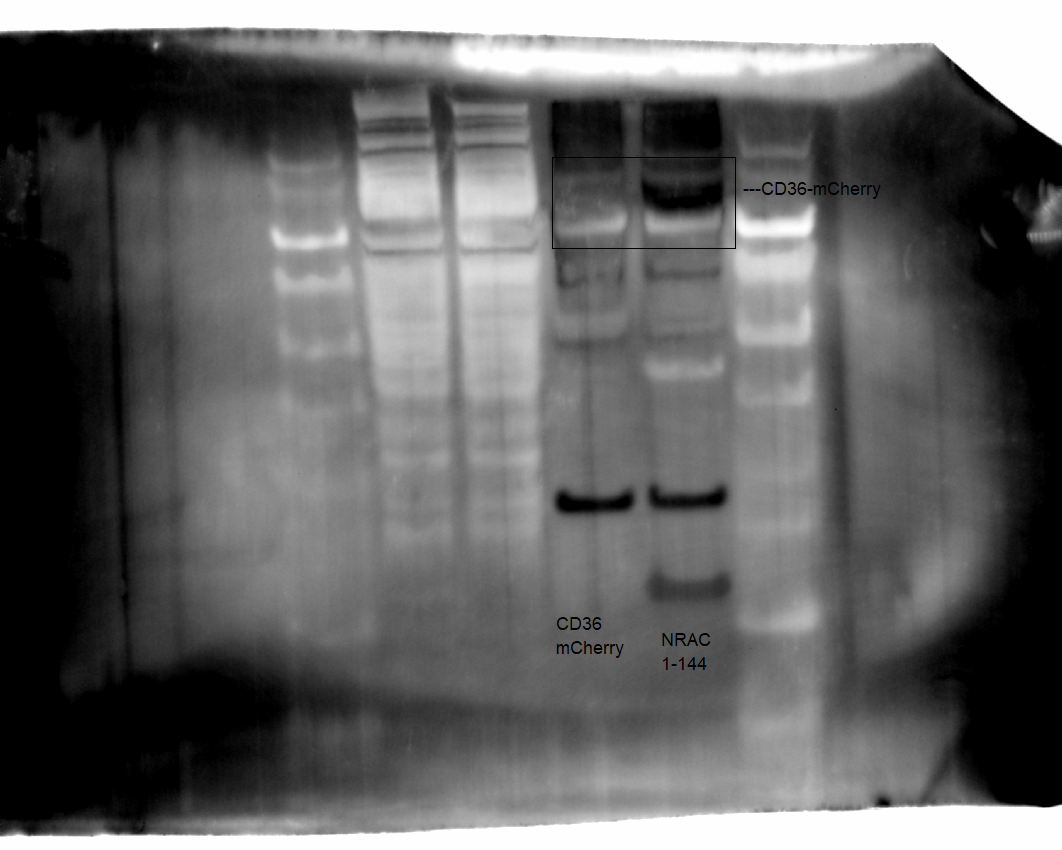

Supplement: Supplementary file 6 — Source data Fig. 4 [file 44318_2025_520_MOESM6_ESM.zip › Figure 4/4B/CD36 Nrac 1-144.tif]

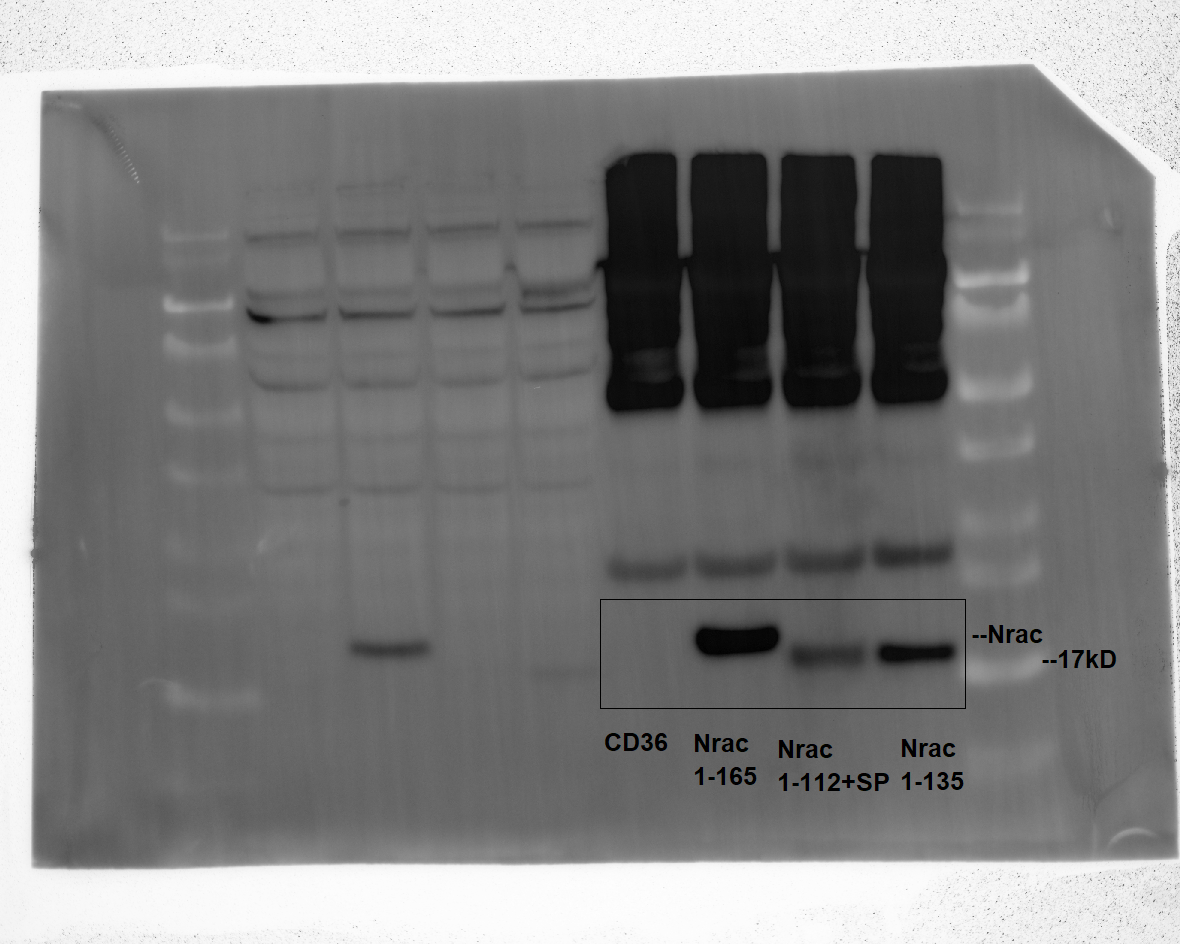

Supplement: Supplementary file 6 — Source data Fig. 4 [file 44318_2025_520_MOESM6_ESM.zip › Figure 4/4B/Nrac.tif]

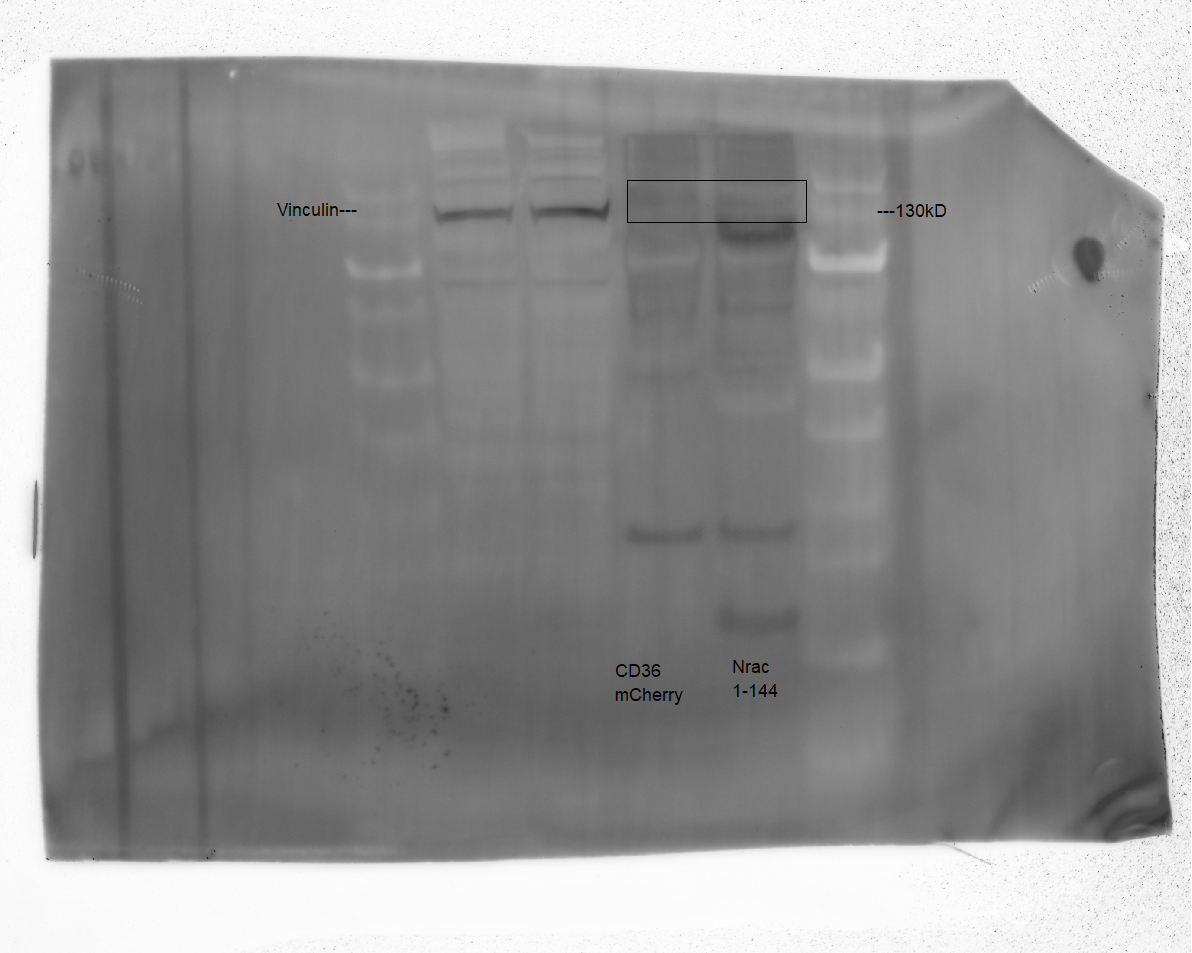

Supplement: Supplementary file 6 — Source data Fig. 4 [file 44318_2025_520_MOESM6_ESM.zip › Figure 4/4B/Vinculin 1-144.tif]

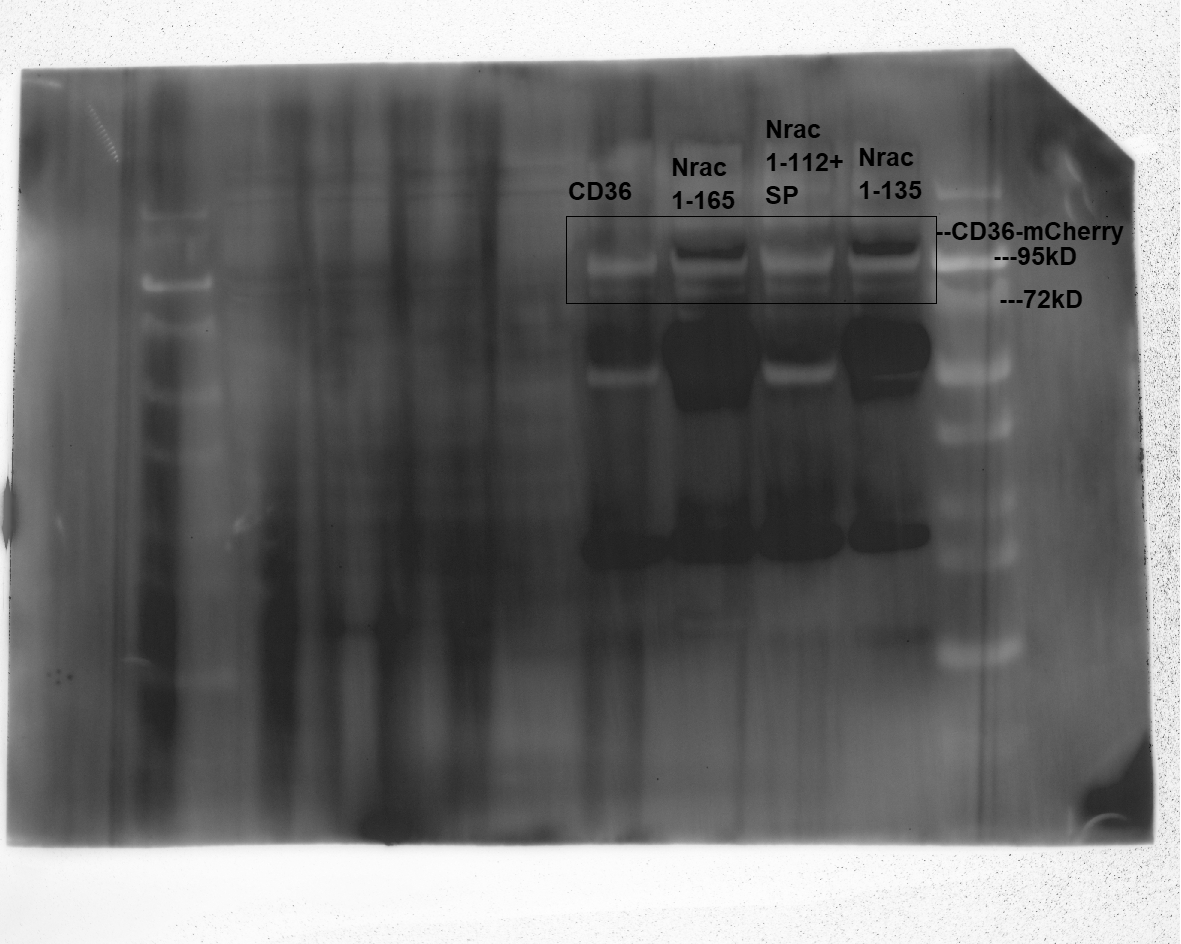

Supplement: Supplementary file 6 — Source data Fig. 4 [file 44318_2025_520_MOESM6_ESM.zip › Figure 4/4B/CD36.tif]

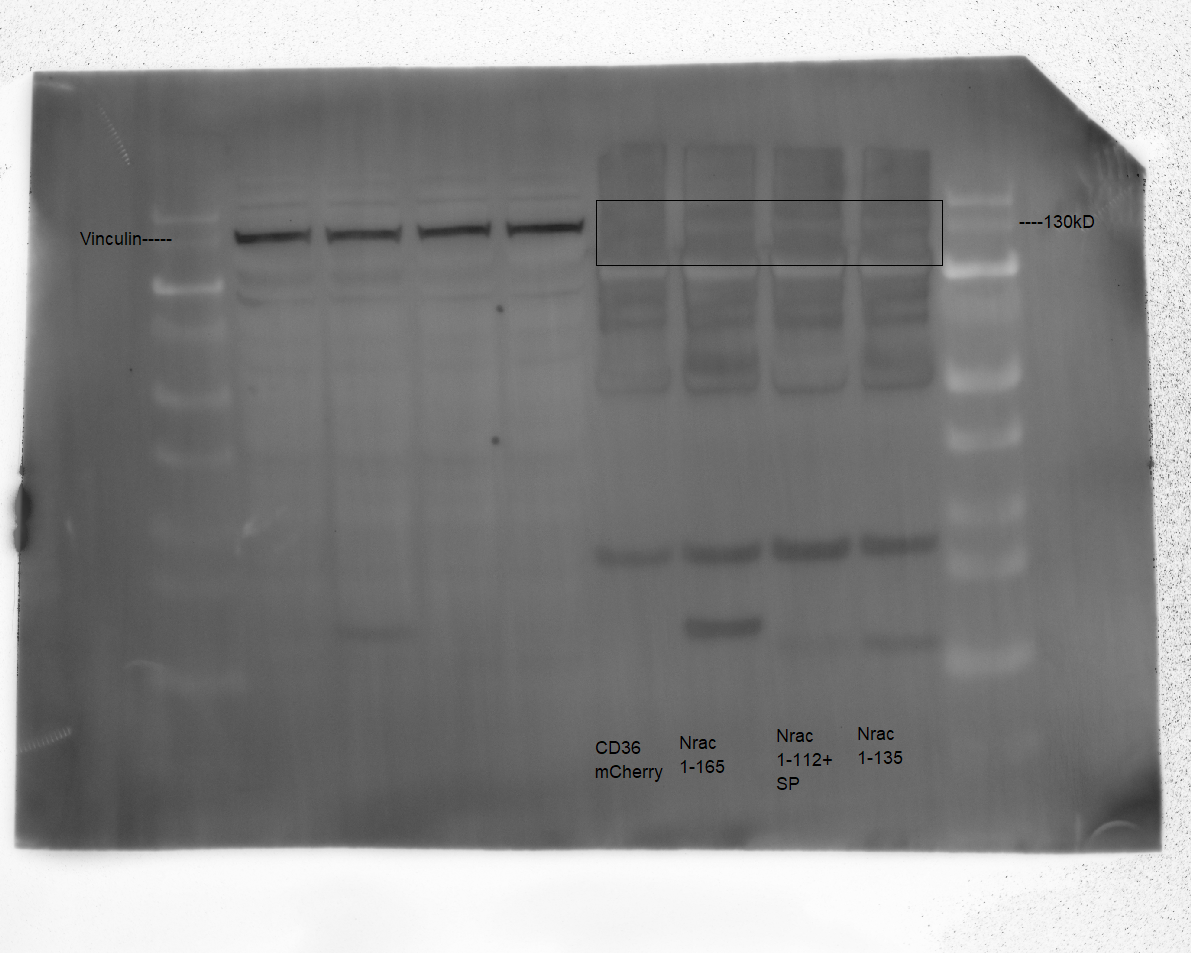

Supplement: Supplementary file 6 — Source data Fig. 4 [file 44318_2025_520_MOESM6_ESM.zip › Figure 4/4B/Vinculin.tif]

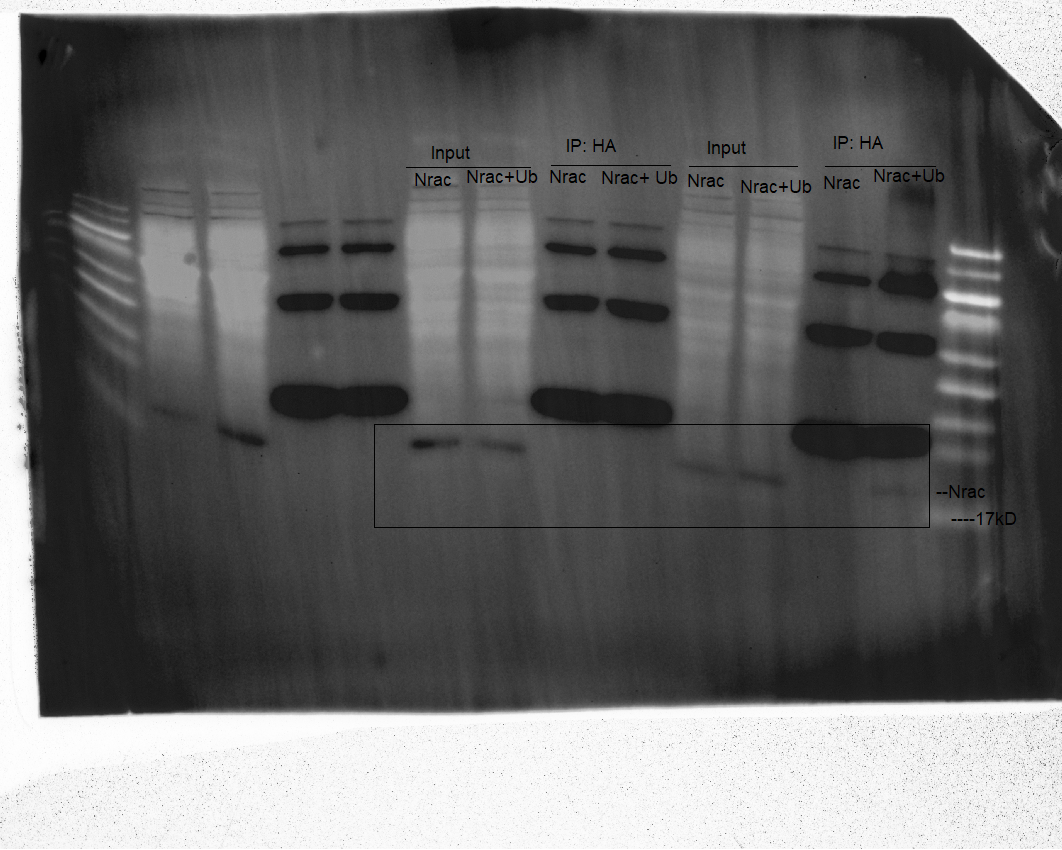

Supplement: Supplementary file 7 — Source data Fig. 5 [file 44318_2025_520_MOESM7_ESM.zip › Figure 5/5I/Nrac.tif]

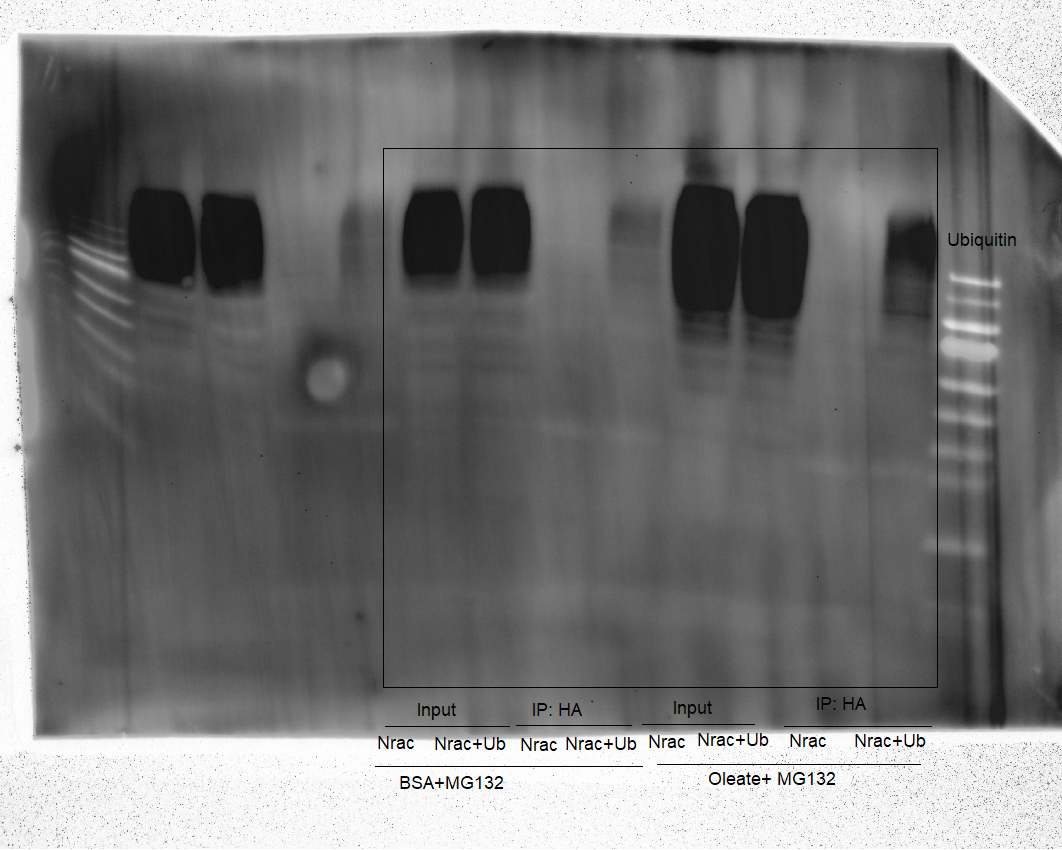

Supplement: Supplementary file 7 — Source data Fig. 5 [file 44318_2025_520_MOESM7_ESM.zip › Figure 5/5I/Ubiquitin.tif]

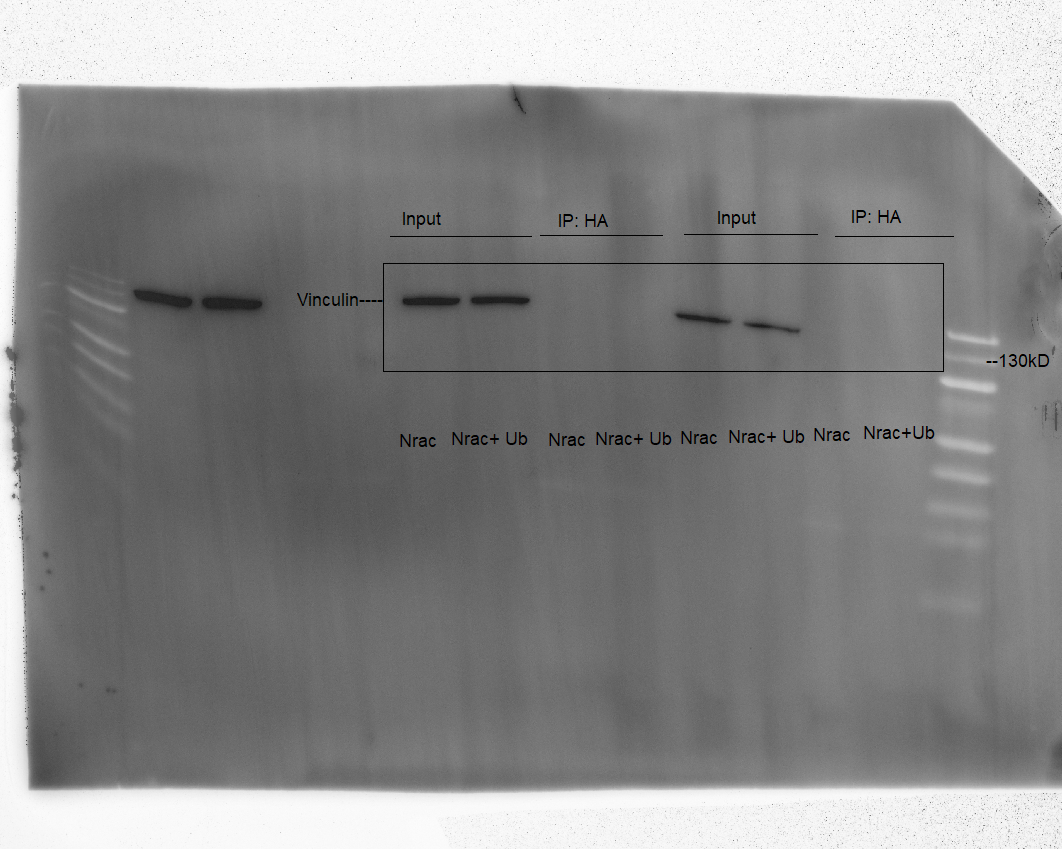

Supplement: Supplementary file 7 — Source data Fig. 5 [file 44318_2025_520_MOESM7_ESM.zip › Figure 5/5I/Vinculin.tif]

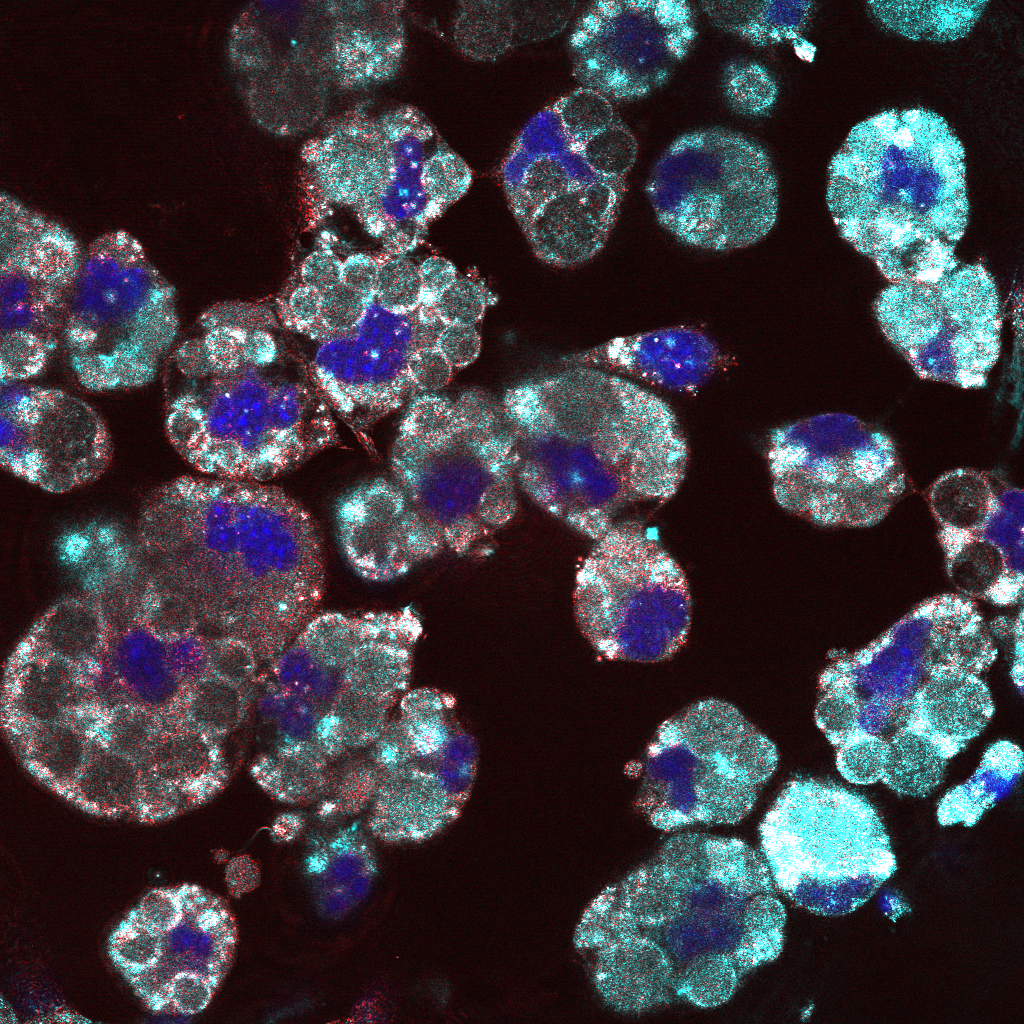

Supplement: Supplementary file 7 — Source data Fig. 5 [file 44318_2025_520_MOESM7_ESM.zip › Figure 5/5G/Experiment_Series099_KO_Oleate_edited.tif]

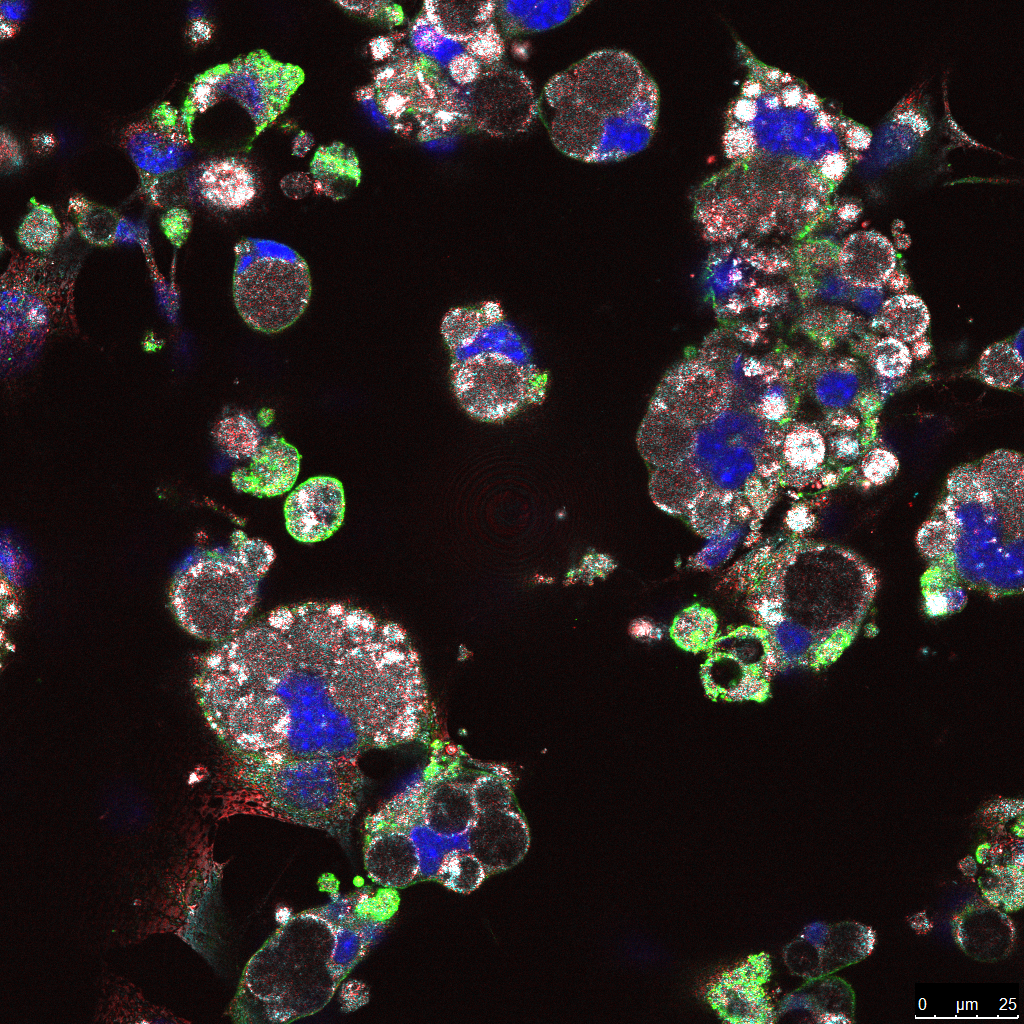

Supplement: Supplementary file 7 — Source data Fig. 5 [file 44318_2025_520_MOESM7_ESM.zip › Figure 5/5G/Experiment_Series063_WT_oleate_z0.tif]

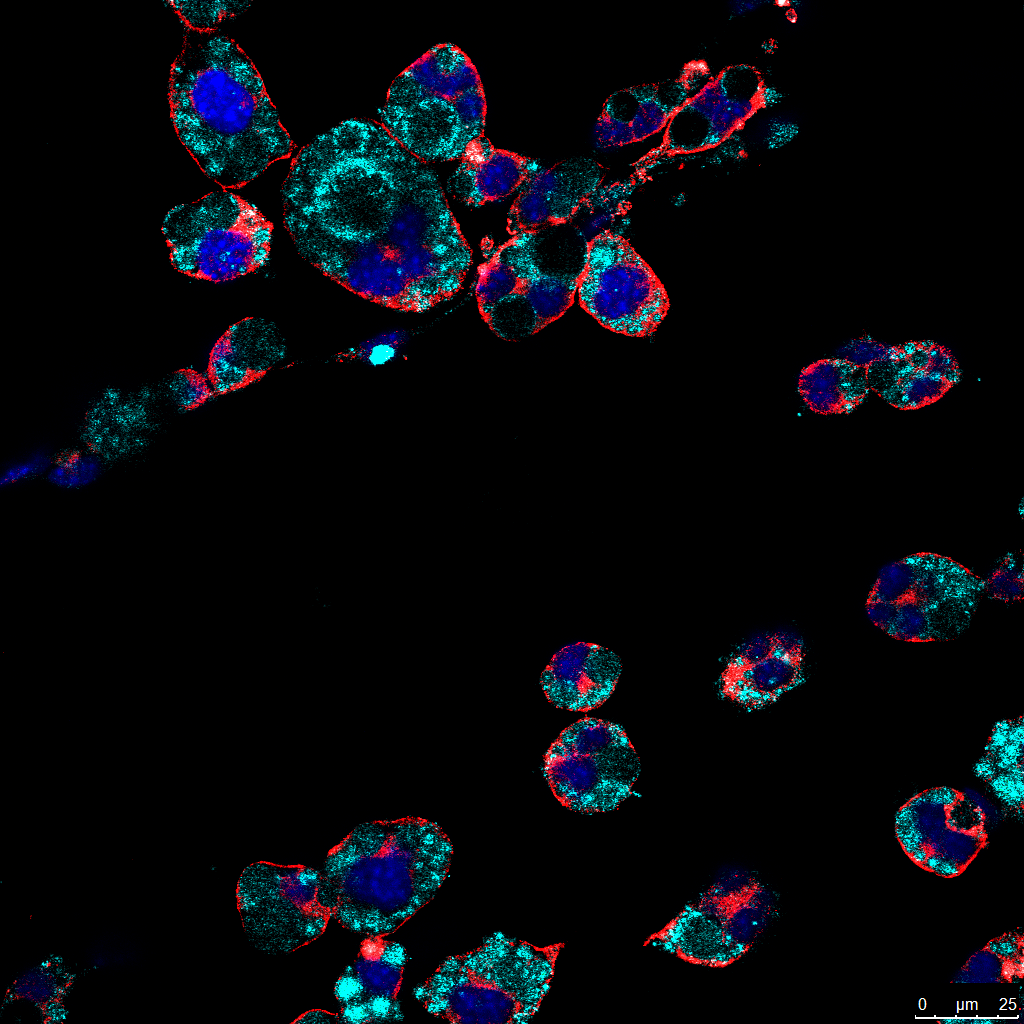

Supplement: Supplementary file 7 — Source data Fig. 5 [file 44318_2025_520_MOESM7_ESM.zip › Figure 5/5G/Experiment_Series081_Nrac KO_Oleate+CPZ_z0.tif]

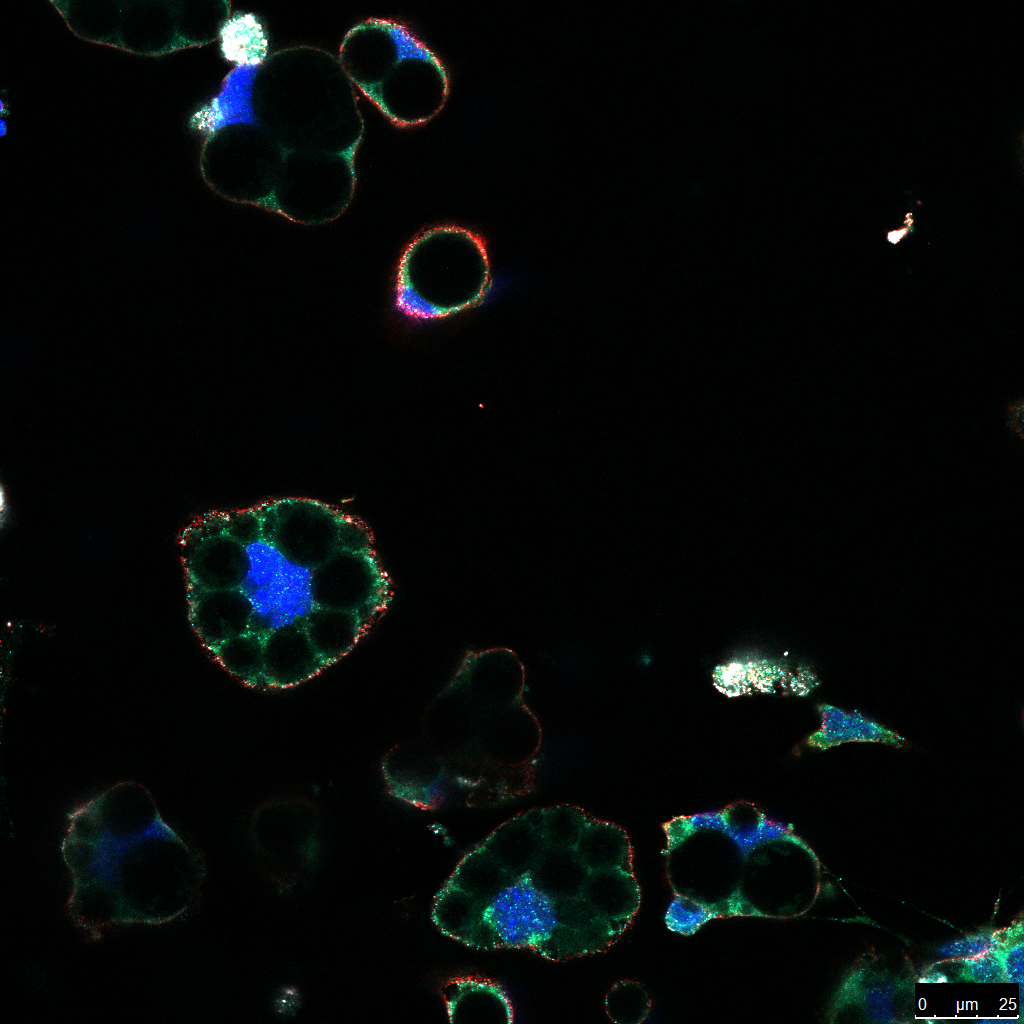

Supplement: Supplementary file 7 — Source data Fig. 5 [file 44318_2025_520_MOESM7_ESM.zip › Figure 5/5G/Experiment_Series037_WT_Oleate+cpz_z0.tif]

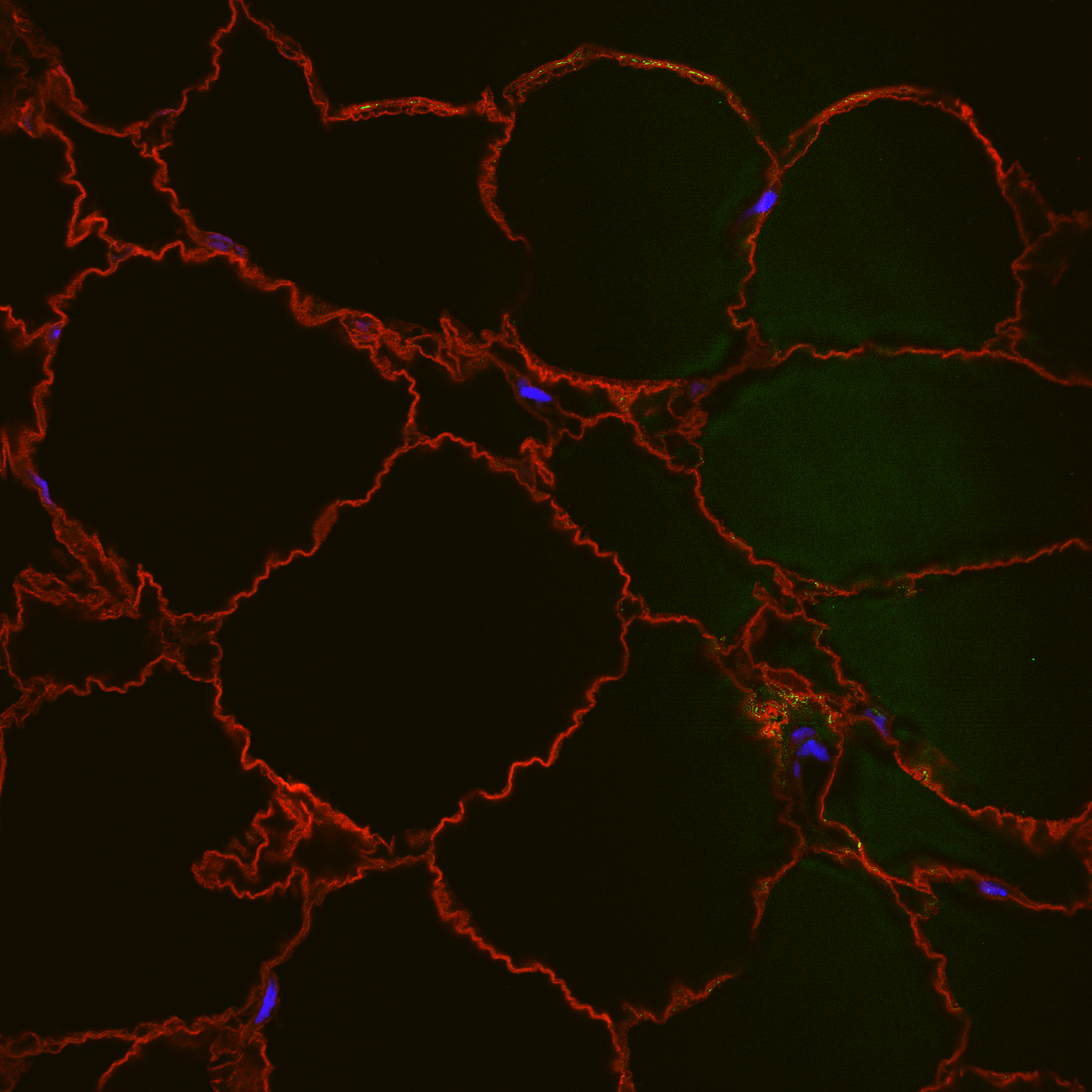

Supplement: Supplementary file 7 — Source data Fig. 5 [file 44318_2025_520_MOESM7_ESM.zip › Figure 5/5H/WT_HFD.tif]

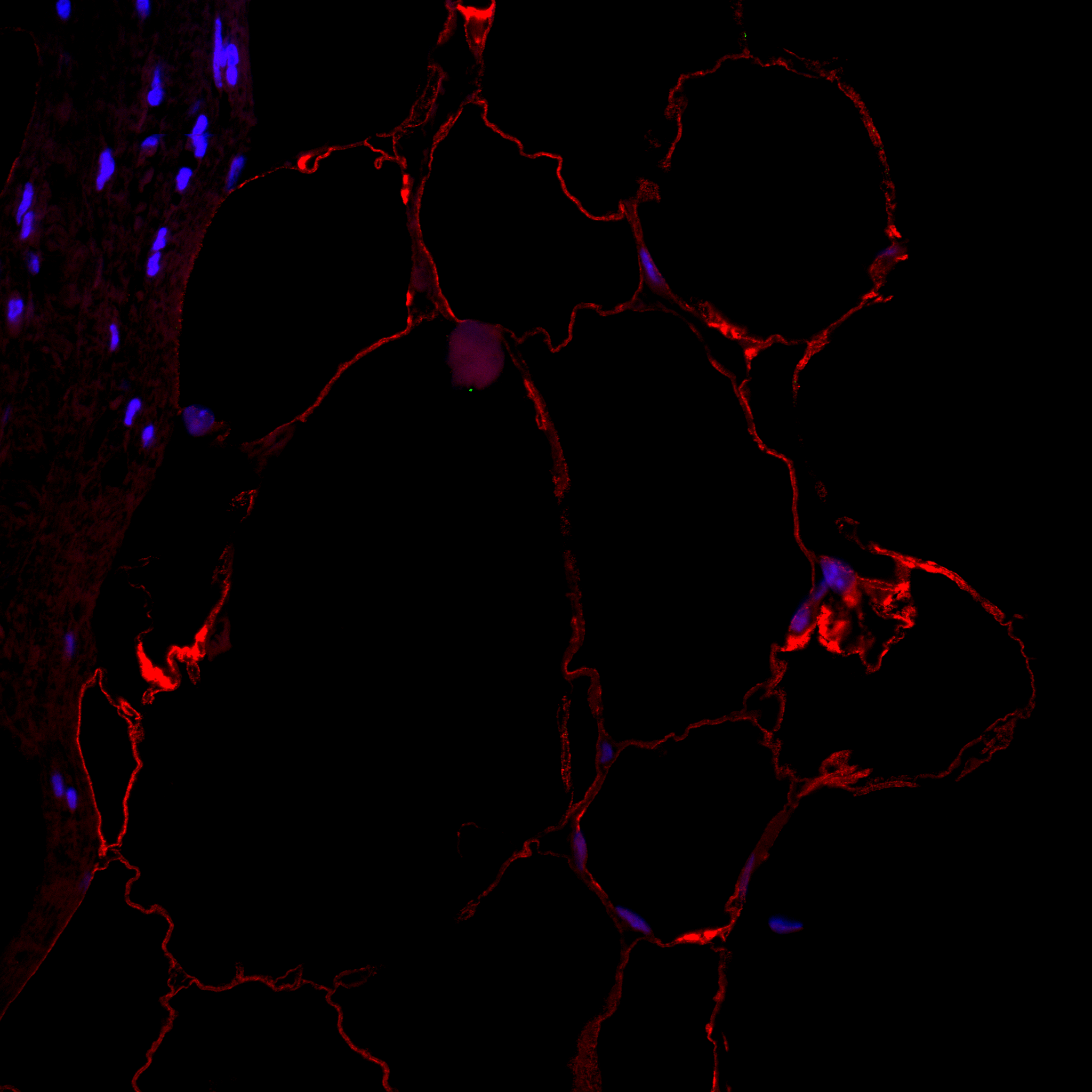

Supplement: Supplementary file 7 — Source data Fig. 5 [file 44318_2025_520_MOESM7_ESM.zip › Figure 5/5H/HFD_KO.tif]

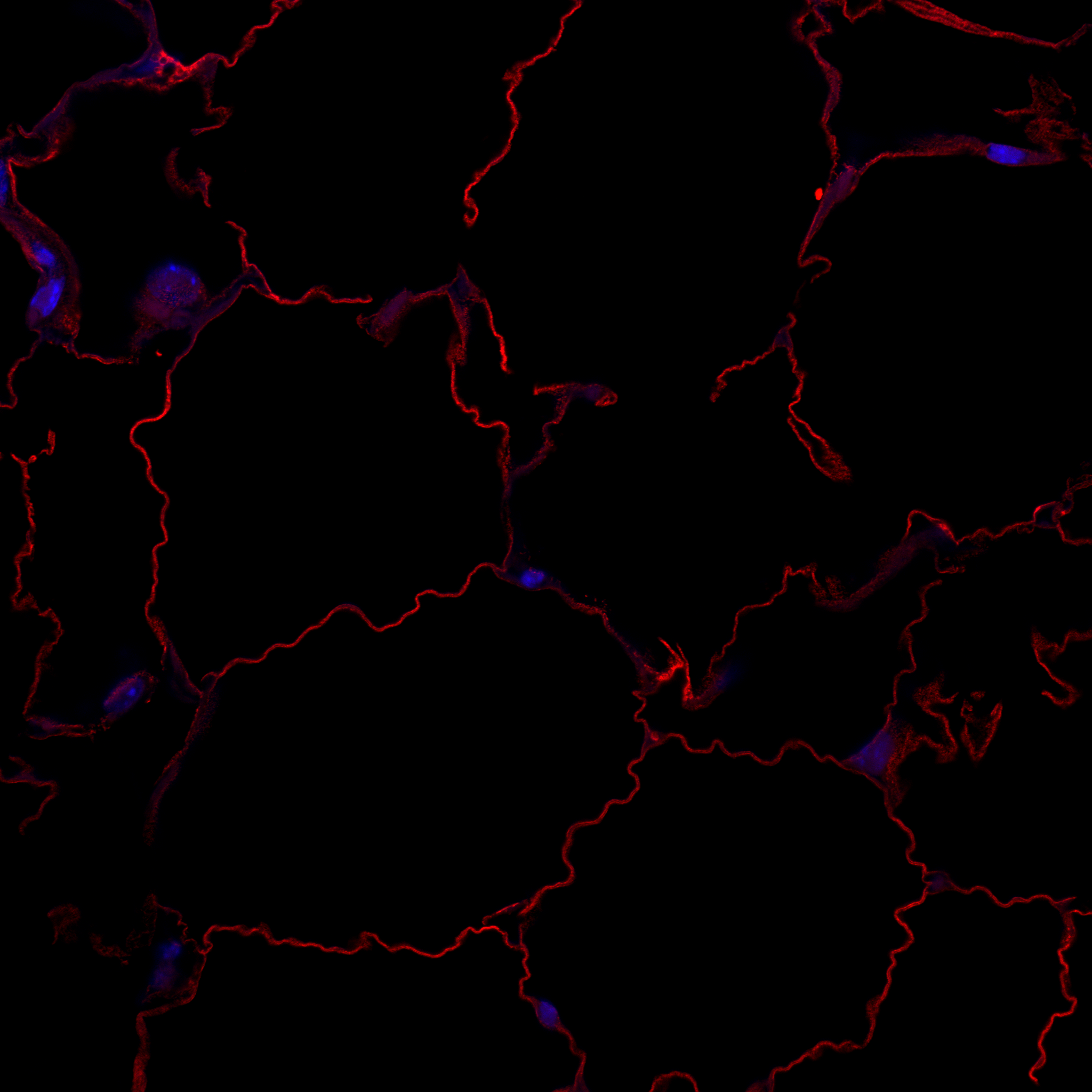

Supplement: Supplementary file 7 — Source data Fig. 5 [file 44318_2025_520_MOESM7_ESM.zip › Figure 5/5H/CD_KO.tif]

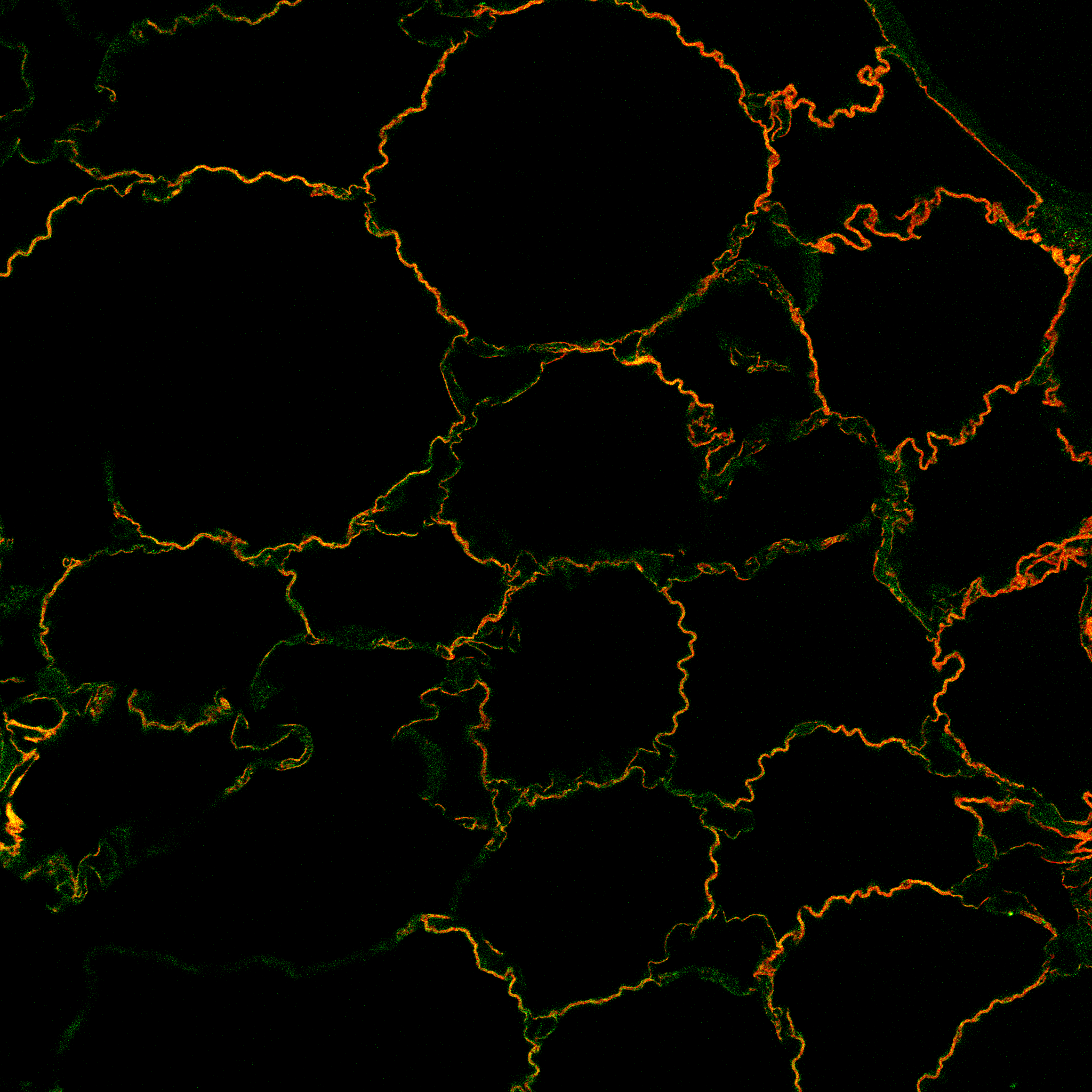

Supplement: Supplementary file 7 — Source data Fig. 5 [file 44318_2025_520_MOESM7_ESM.zip › Figure 5/5H/CD_WT.tif]

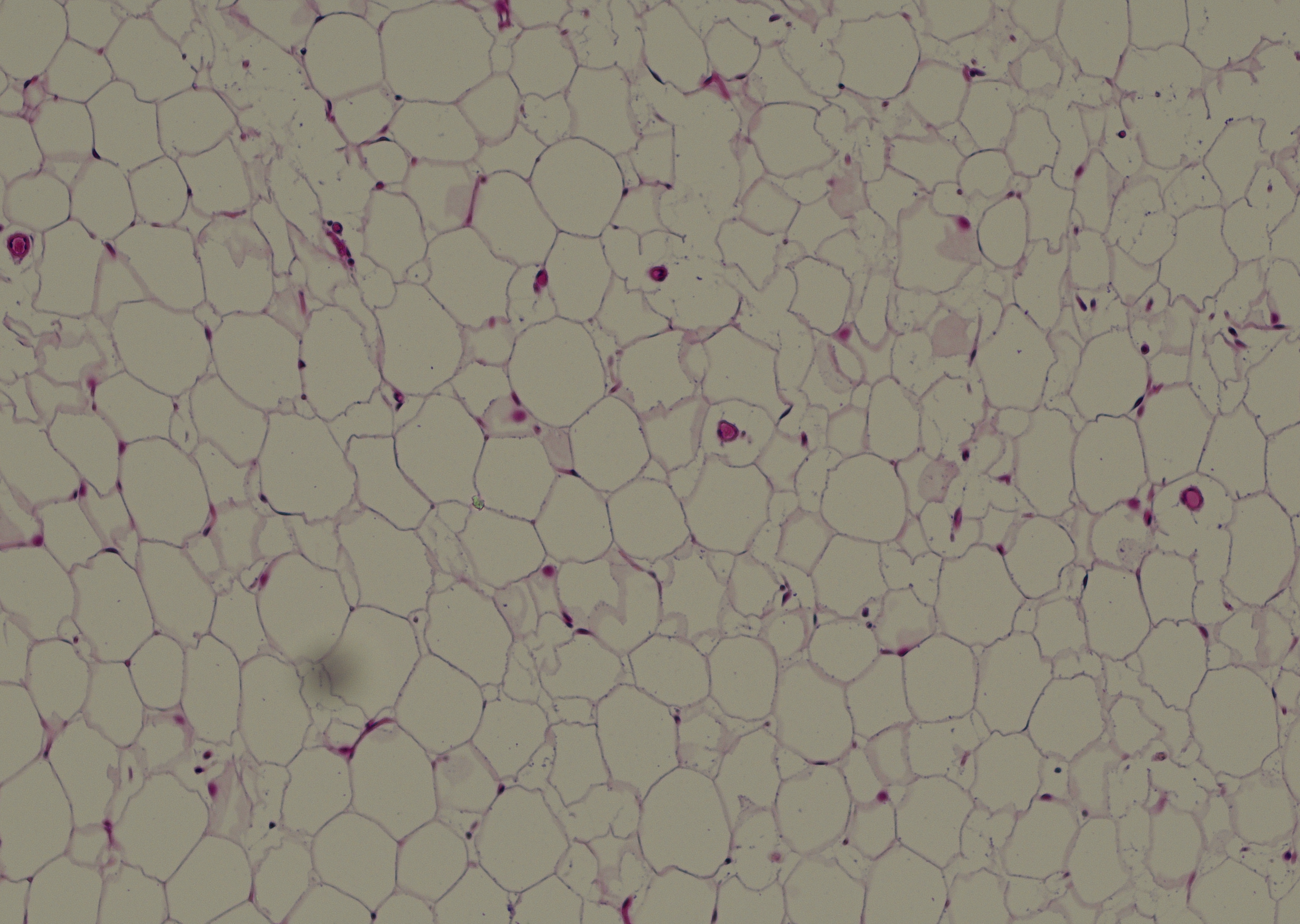

Supplement: Supplementary file 8 — Figure EV1 Source Data [file 44318_2025_520_MOESM8_ESM.zip › EV1/1S/PG KO.tif]

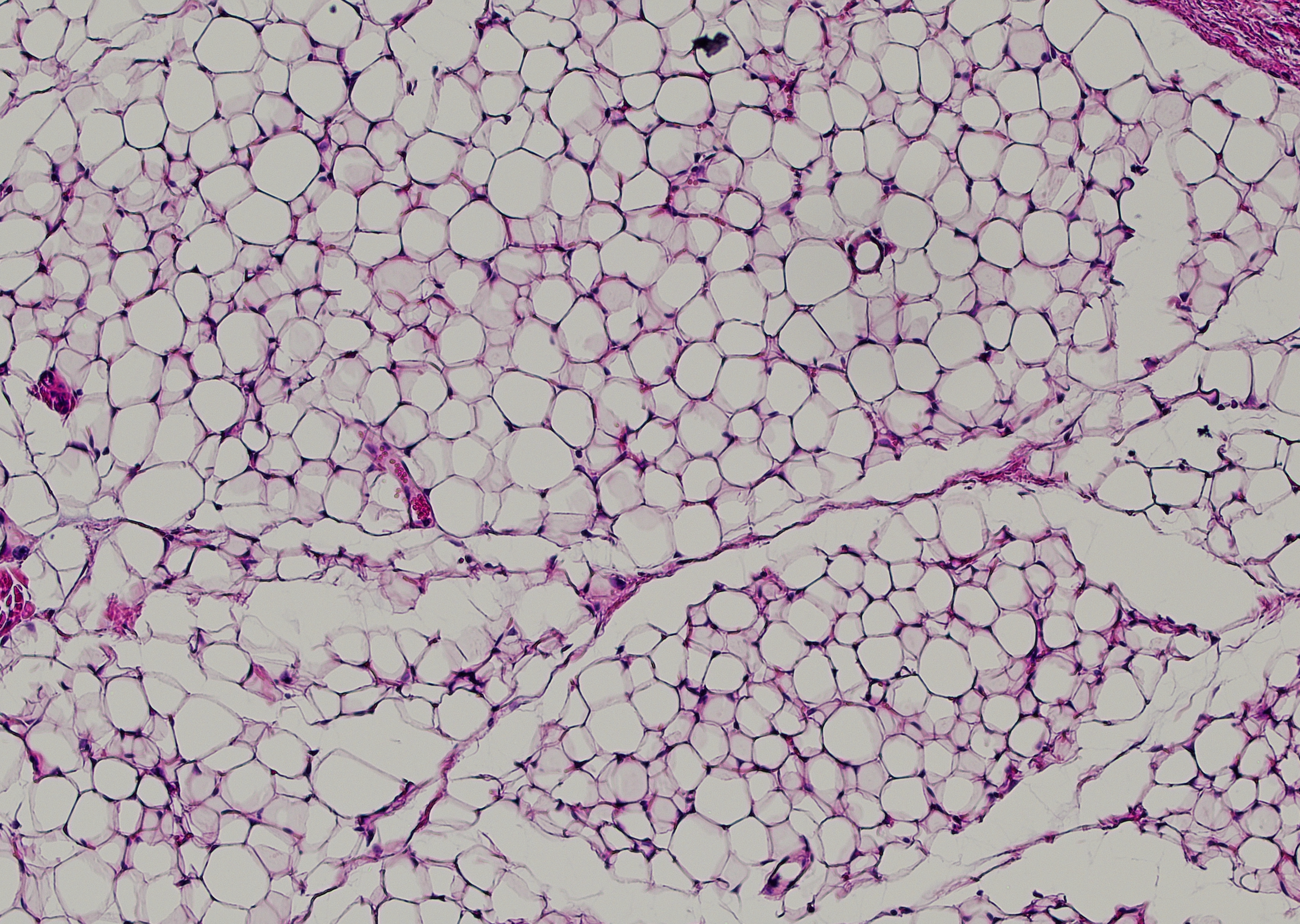

Supplement: Supplementary file 8 — Figure EV1 Source Data [file 44318_2025_520_MOESM8_ESM.zip › EV1/1S/SC_KO.tif]

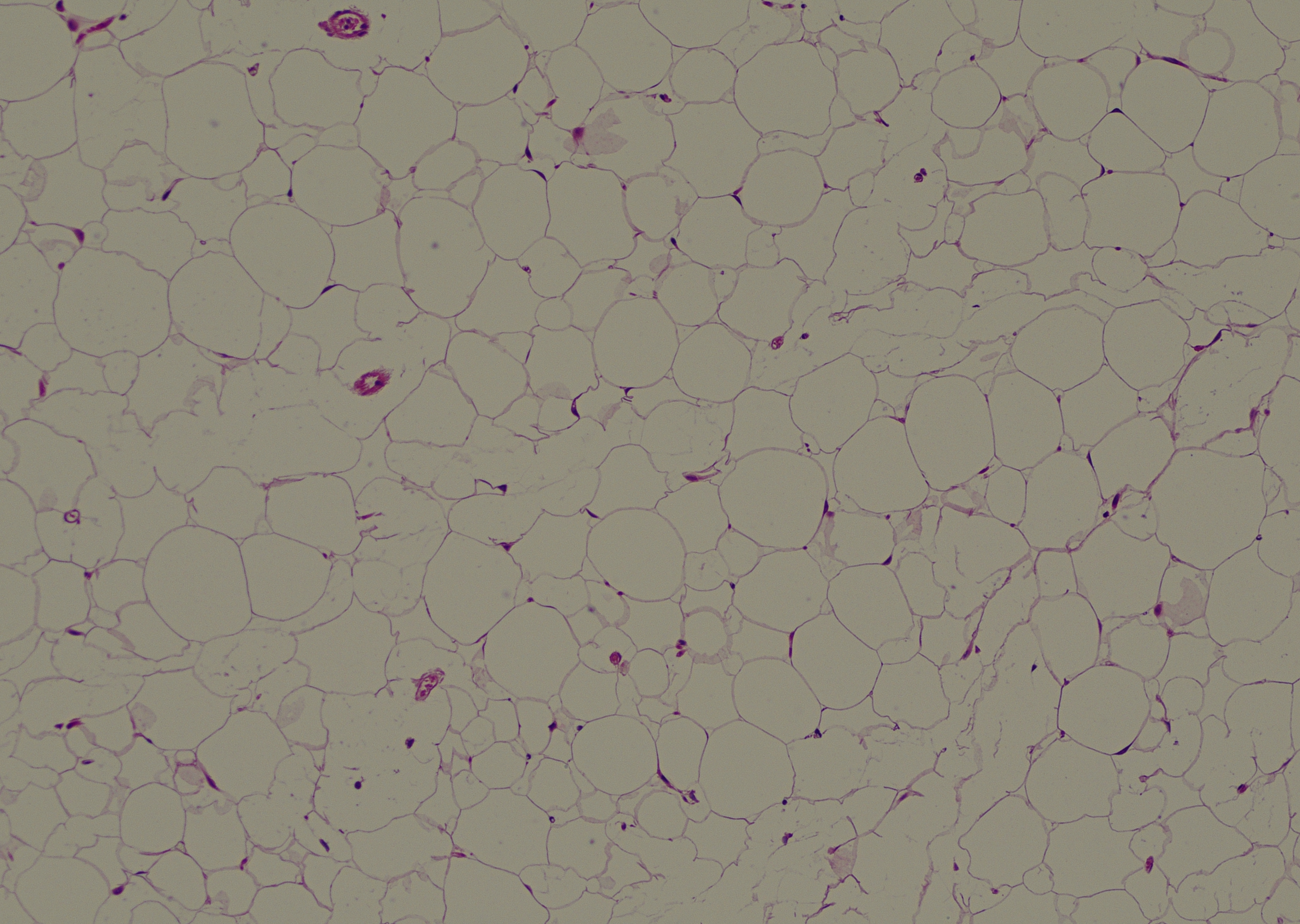

Supplement: Supplementary file 8 — Figure EV1 Source Data [file 44318_2025_520_MOESM8_ESM.zip › EV1/1S/PG_WT.tif]

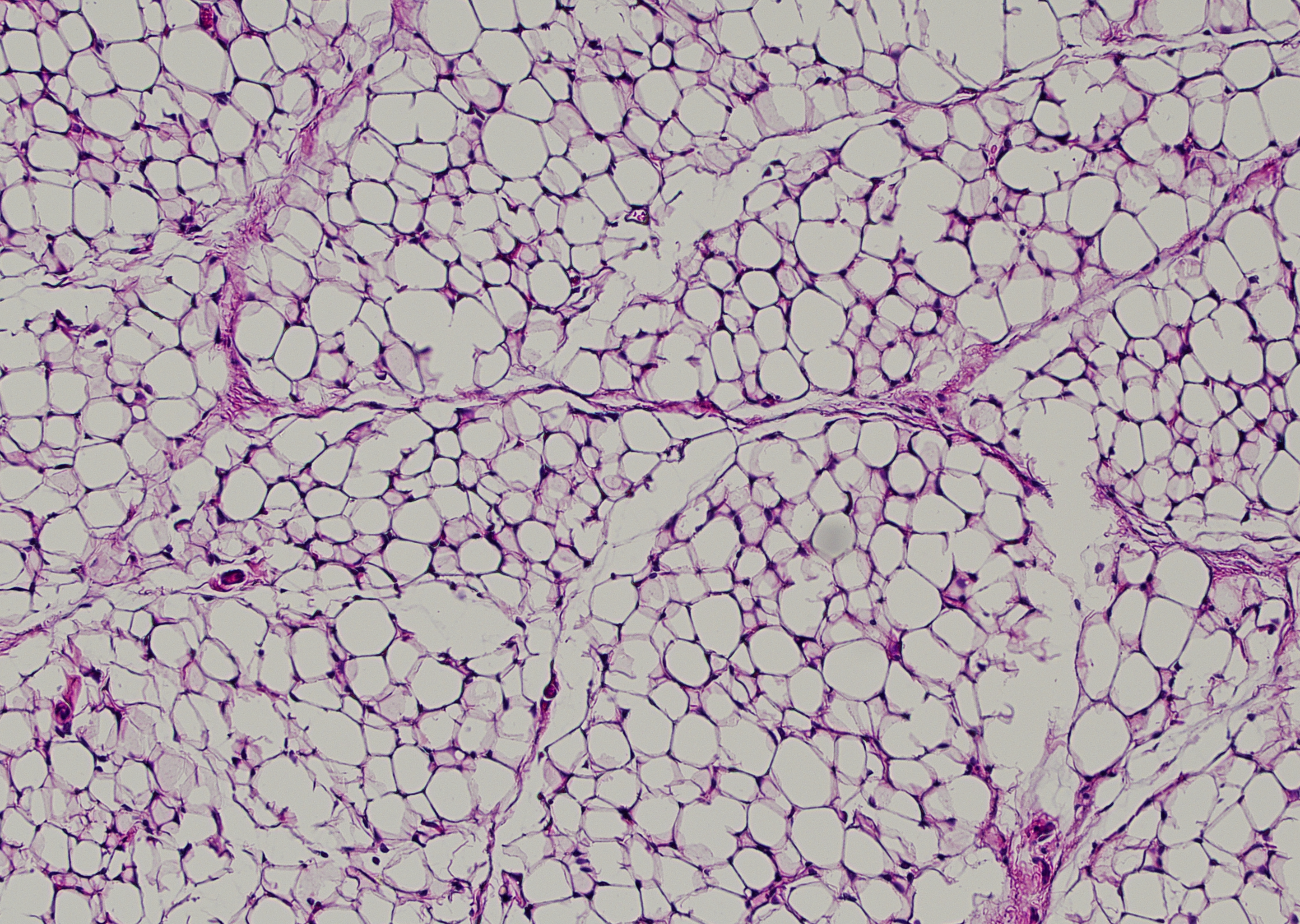

Supplement: Supplementary file 8 — Figure EV1 Source Data [file 44318_2025_520_MOESM8_ESM.zip › EV1/1S/SC_WT.tif]

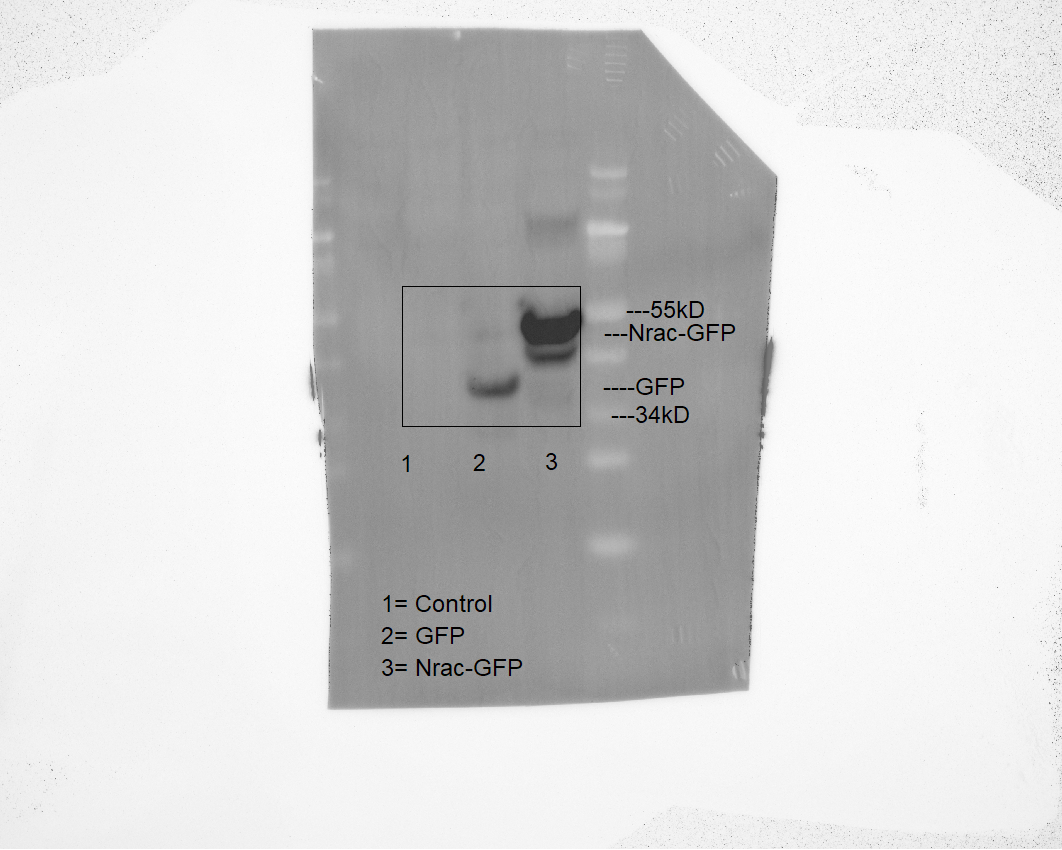

Supplement: Supplementary file 8 — Figure EV1 Source Data [file 44318_2025_520_MOESM8_ESM.zip › EV1/1A/GFP.tif]

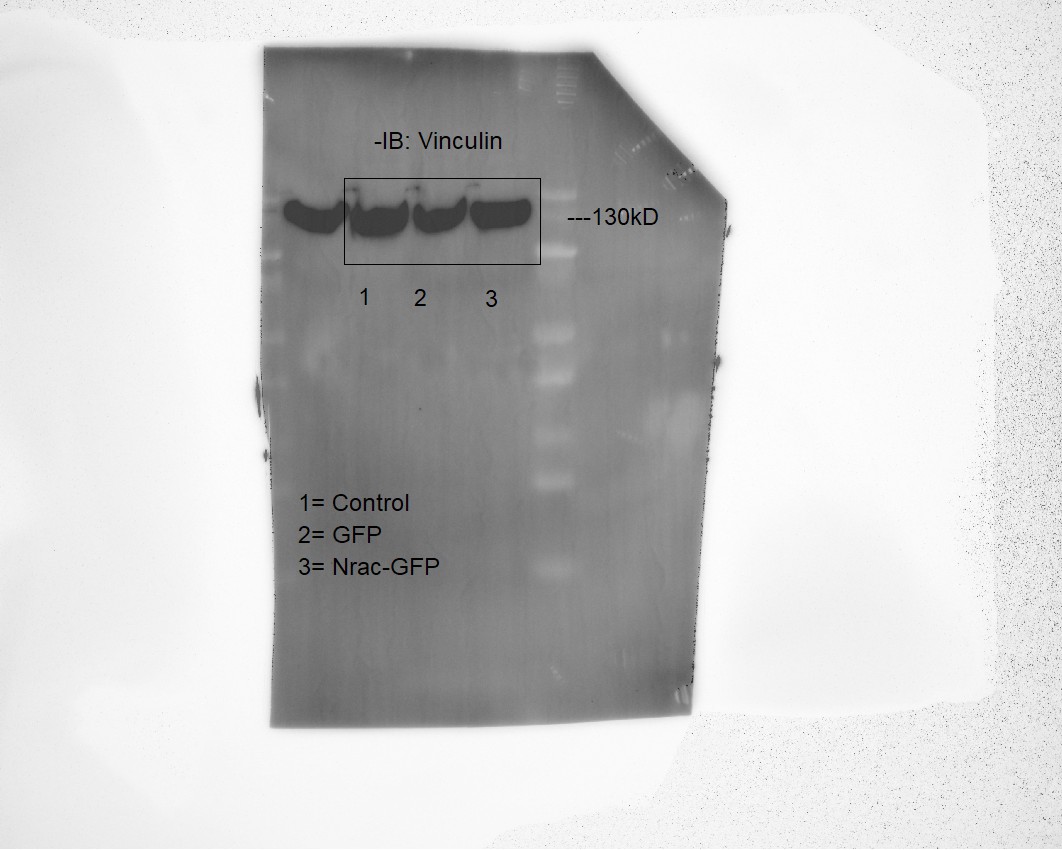

Supplement: Supplementary file 8 — Figure EV1 Source Data [file 44318_2025_520_MOESM8_ESM.zip › EV1/1A/Vinculin.tif]

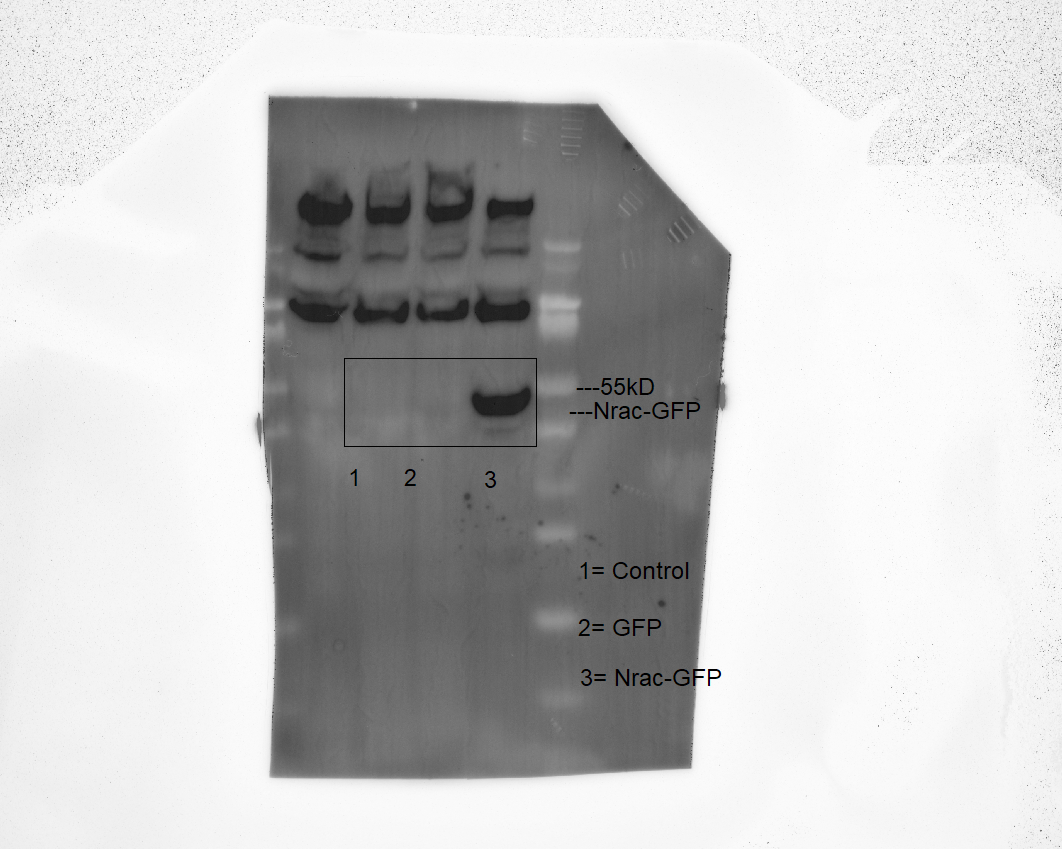

Supplement: Supplementary file 8 — Figure EV1 Source Data [file 44318_2025_520_MOESM8_ESM.zip › EV1/1A/NRAC-GFP.tif]

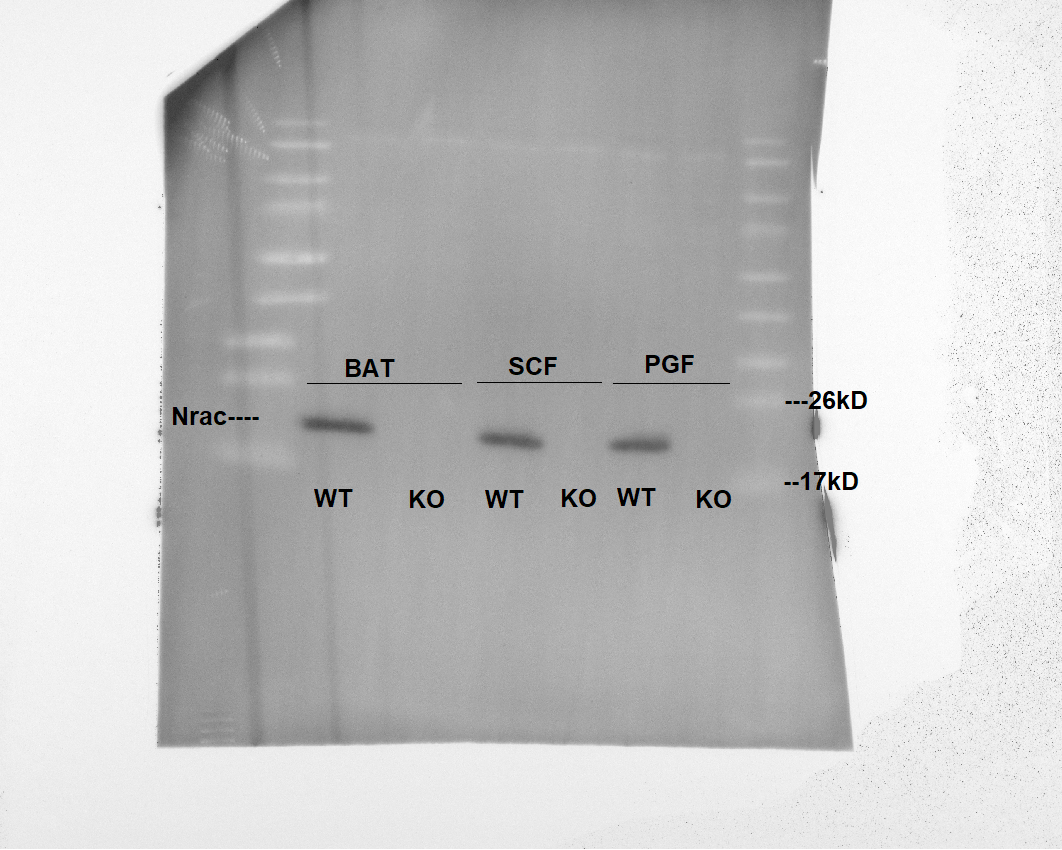

Supplement: Supplementary file 8 — Figure EV1 Source Data [file 44318_2025_520_MOESM8_ESM.zip › EV1/1B/NRAC.tif]

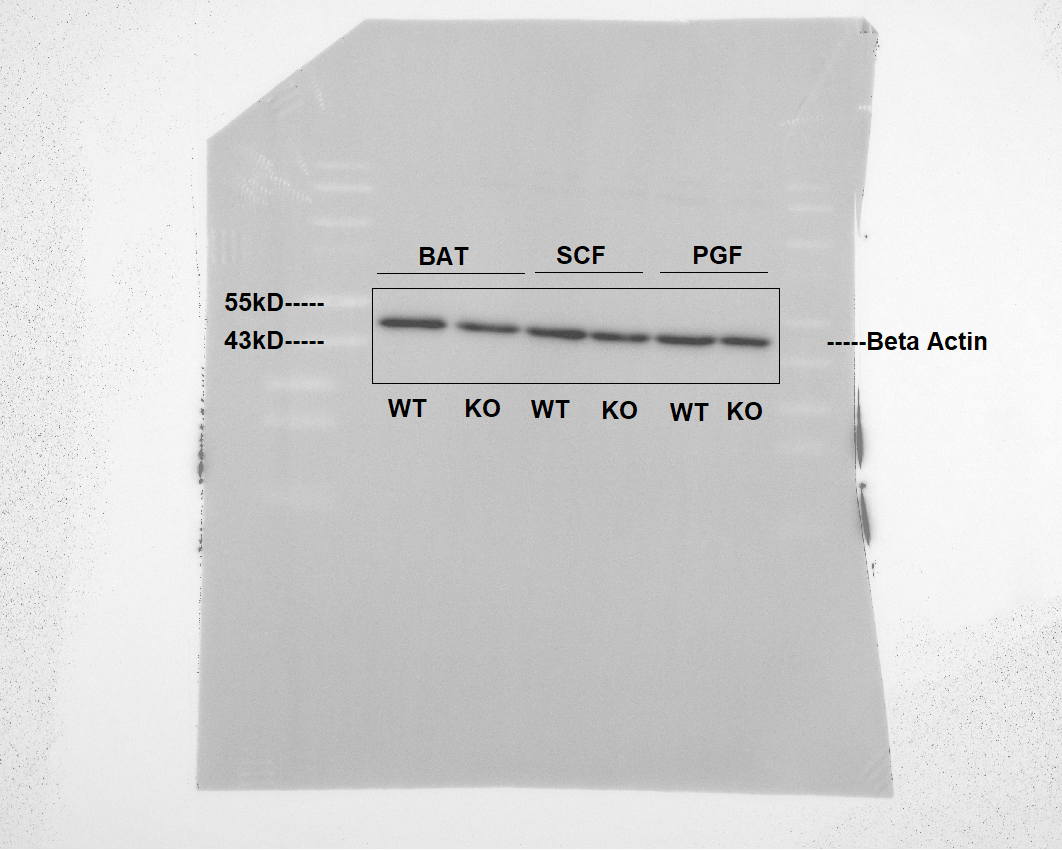

Supplement: Supplementary file 8 — Figure EV1 Source Data [file 44318_2025_520_MOESM8_ESM.zip › EV1/1B/Beta Actin.tif]

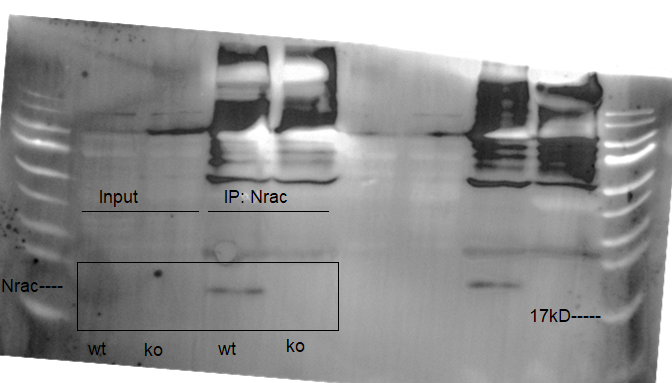

Supplement: Supplementary file 8 — Figure EV1 Source Data [file 44318_2025_520_MOESM8_ESM.zip › EV1/1D/edited_IP Nrac.tif]

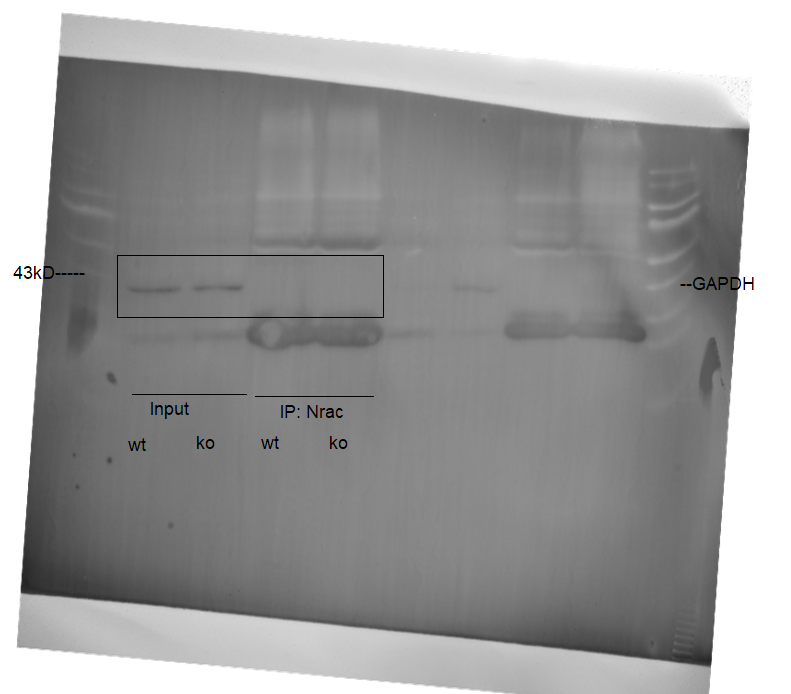

Supplement: Supplementary file 8 — Figure EV1 Source Data [file 44318_2025_520_MOESM8_ESM.zip › EV1/1D/edited_GAPDH.tif]

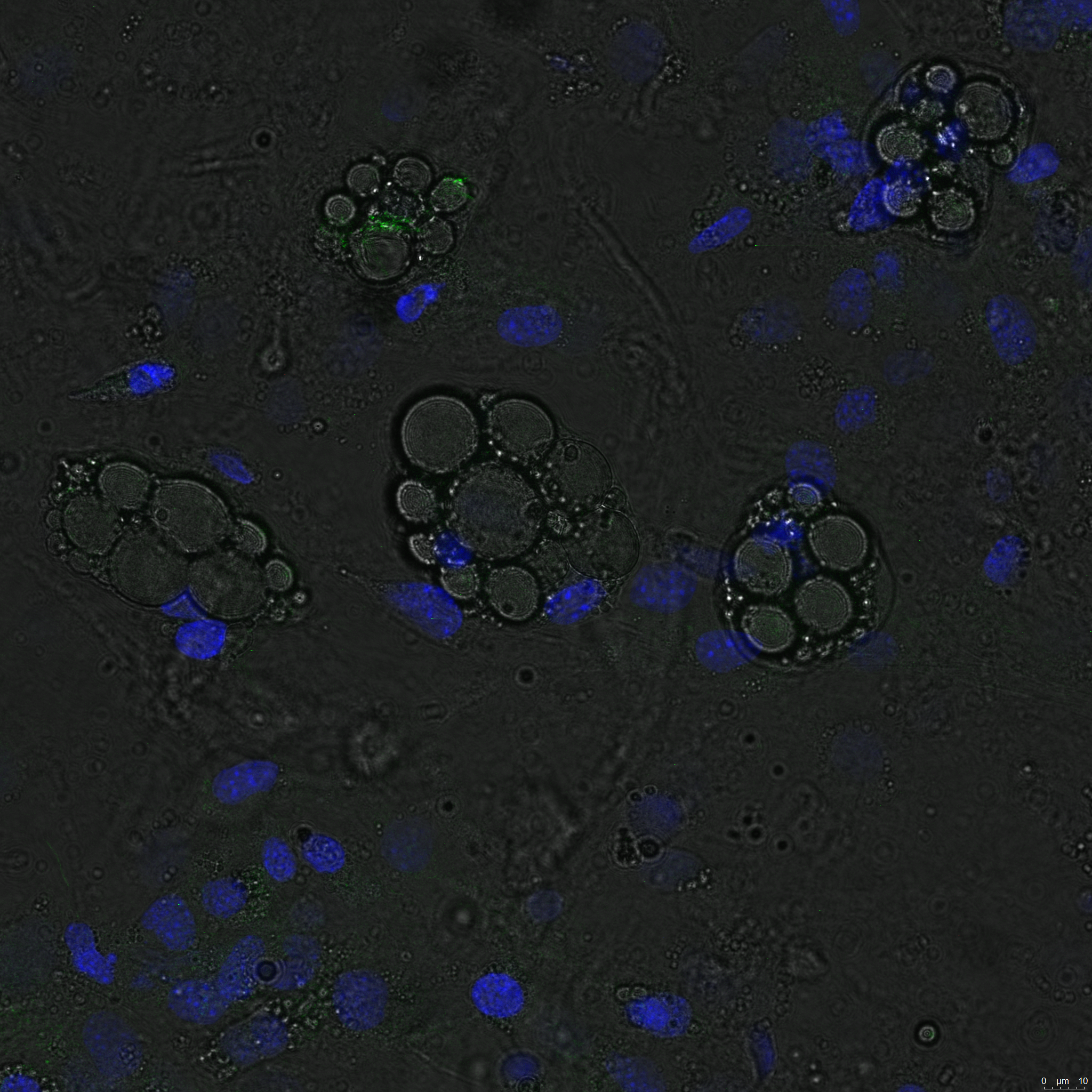

Supplement: Supplementary file 8 — Figure EV1 Source Data [file 44318_2025_520_MOESM8_ESM.zip › EV1/1C/Nrac_KO.tif]

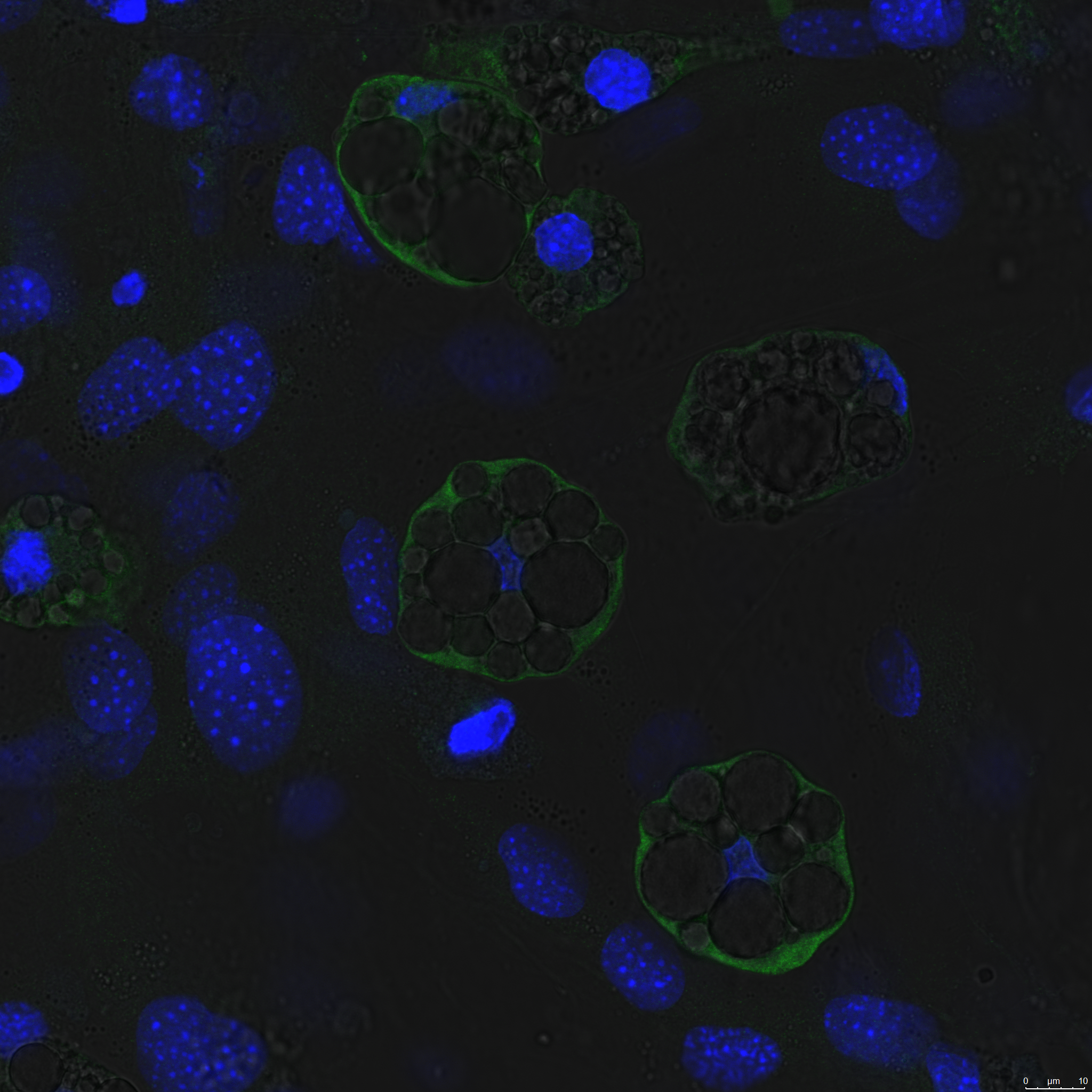

Supplement: Supplementary file 8 — Figure EV1 Source Data [file 44318_2025_520_MOESM8_ESM.zip › EV1/1C/WT.tif]

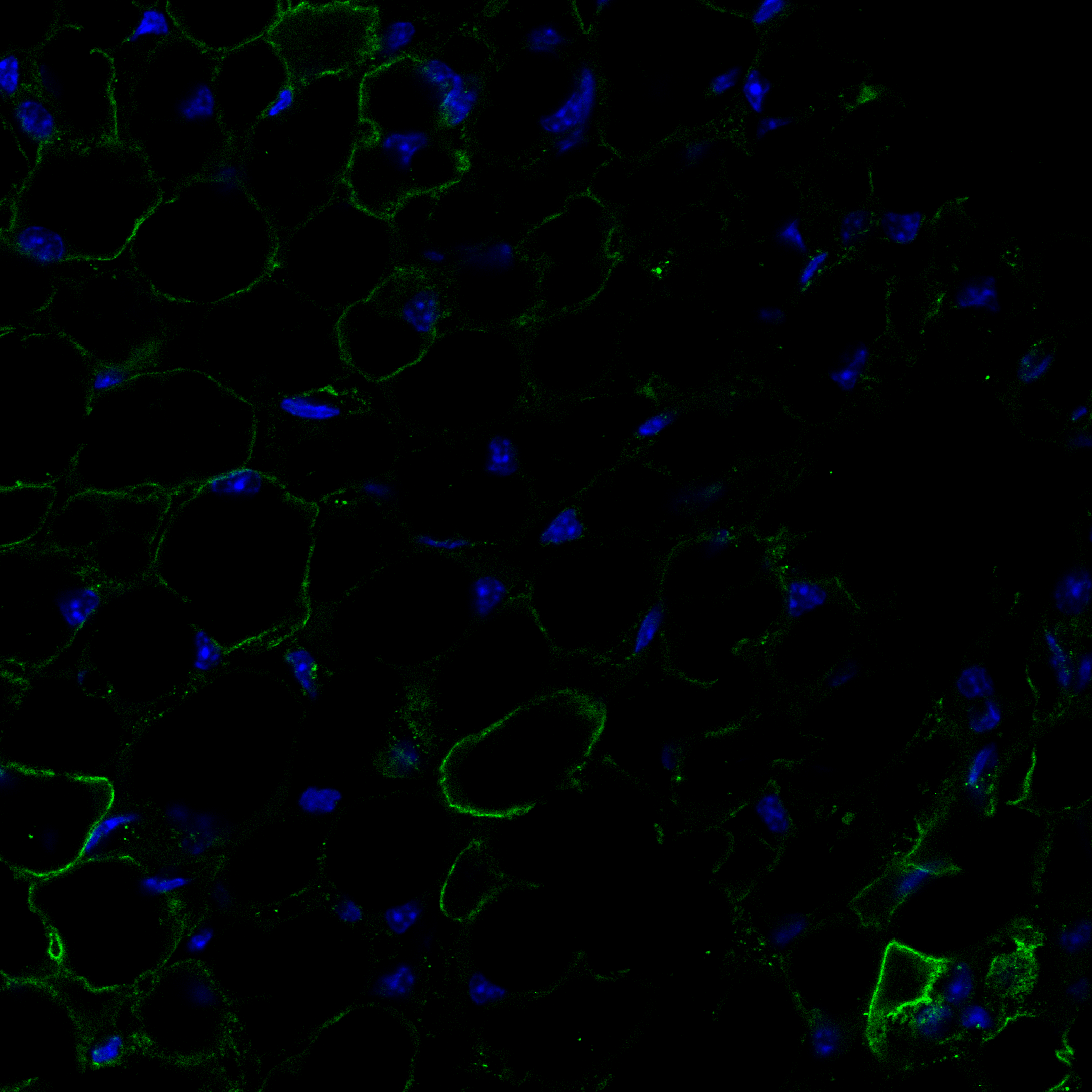

Supplement: Supplementary file 9 — Figure EV2 Source Data [file 44318_2025_520_MOESM9_ESM.zip › EV2/2D/CL316243.tif]

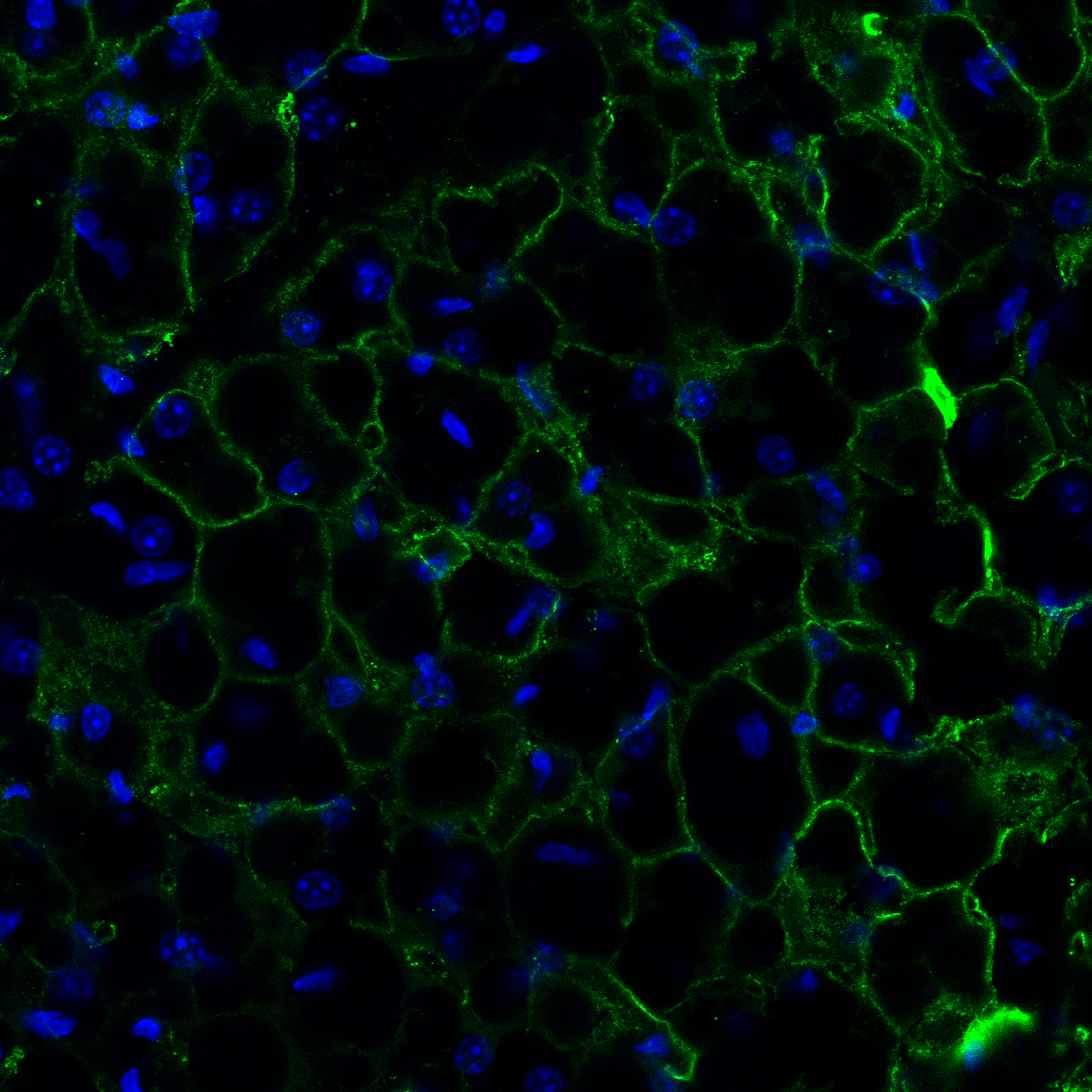

Supplement: Supplementary file 9 — Figure EV2 Source Data [file 44318_2025_520_MOESM9_ESM.zip › EV2/2D/Starved.tif]

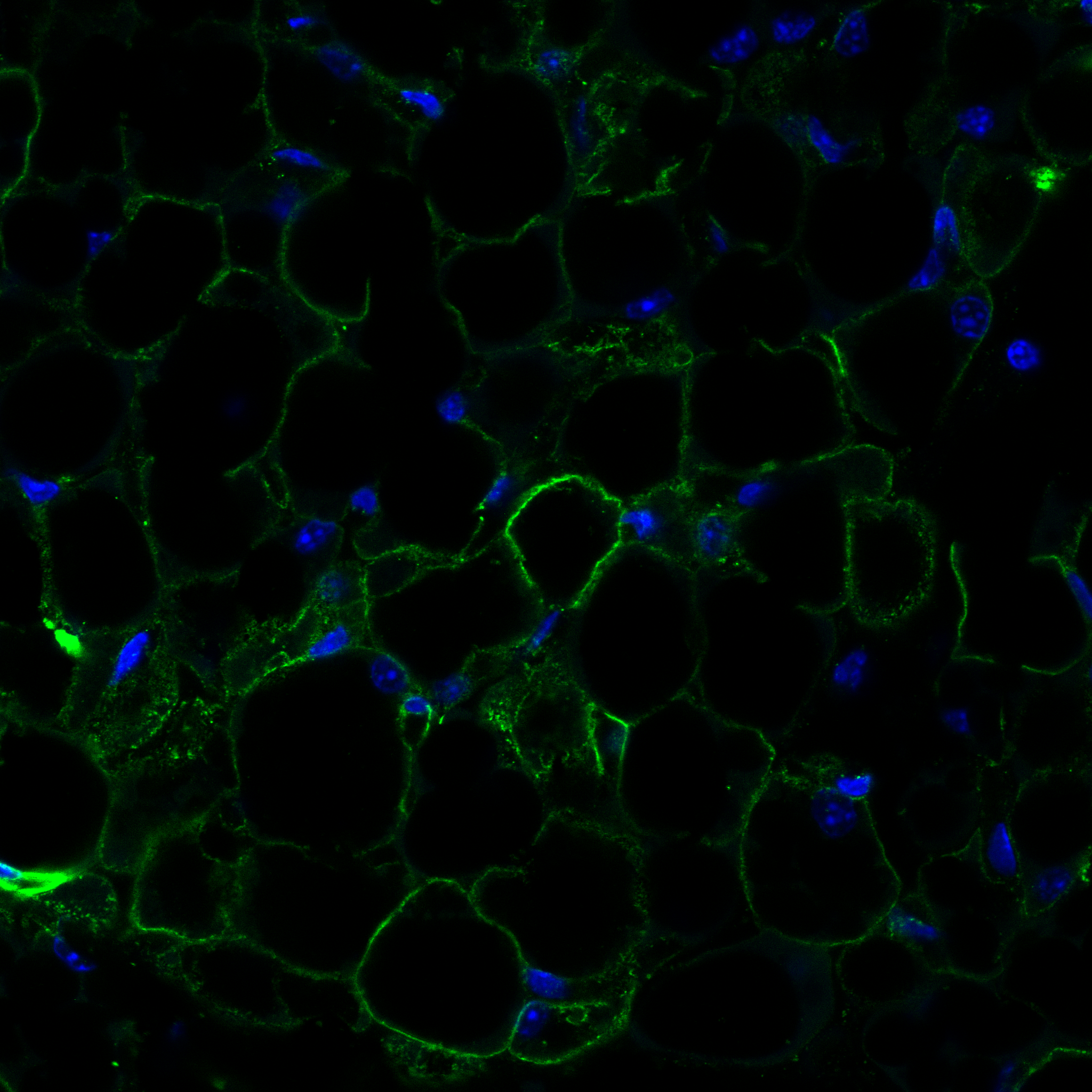

Supplement: Supplementary file 9 — Figure EV2 Source Data [file 44318_2025_520_MOESM9_ESM.zip › EV2/2D/Random fed.tif]

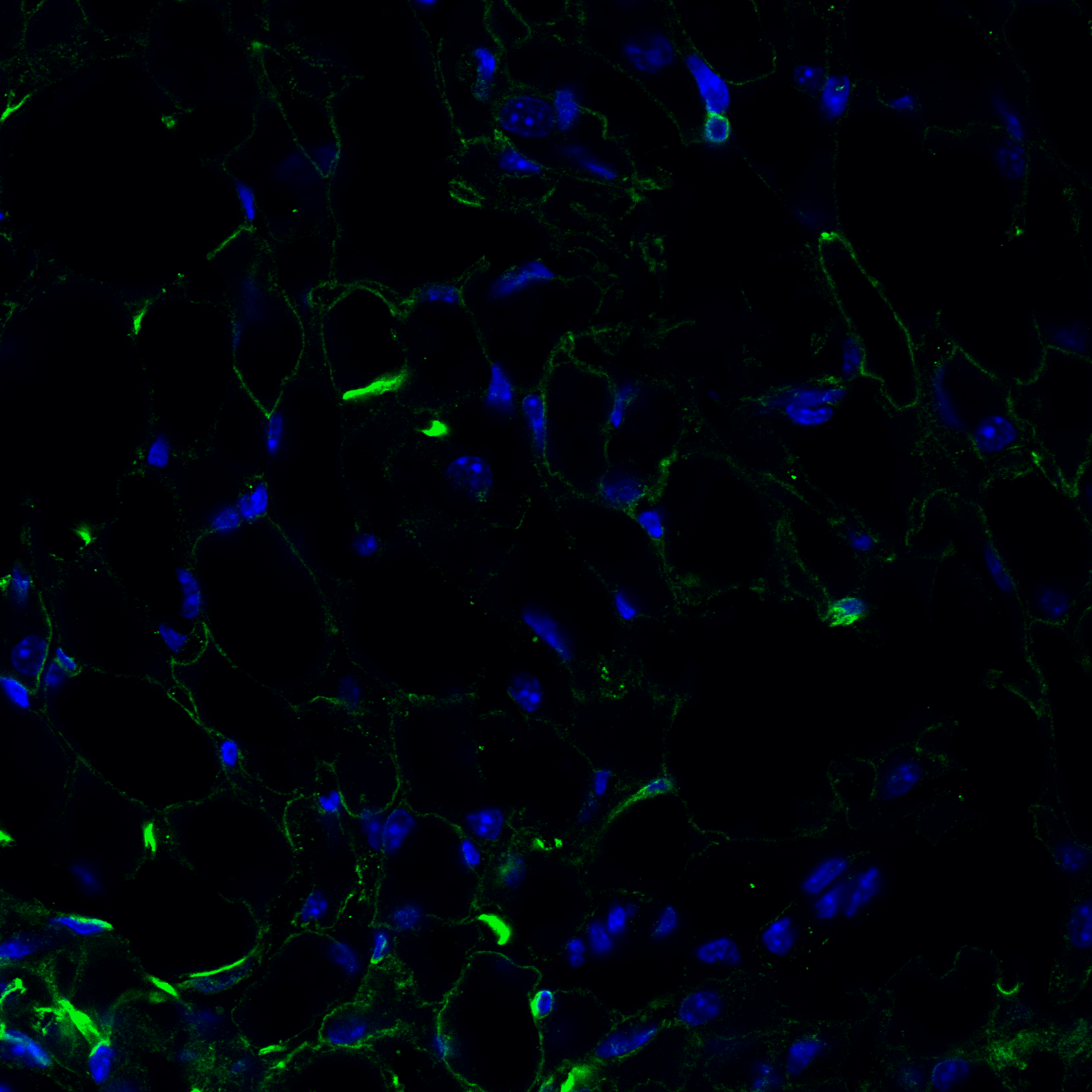

Supplement: Supplementary file 9 — Figure EV2 Source Data [file 44318_2025_520_MOESM9_ESM.zip › EV2/2D/Cold.tif]

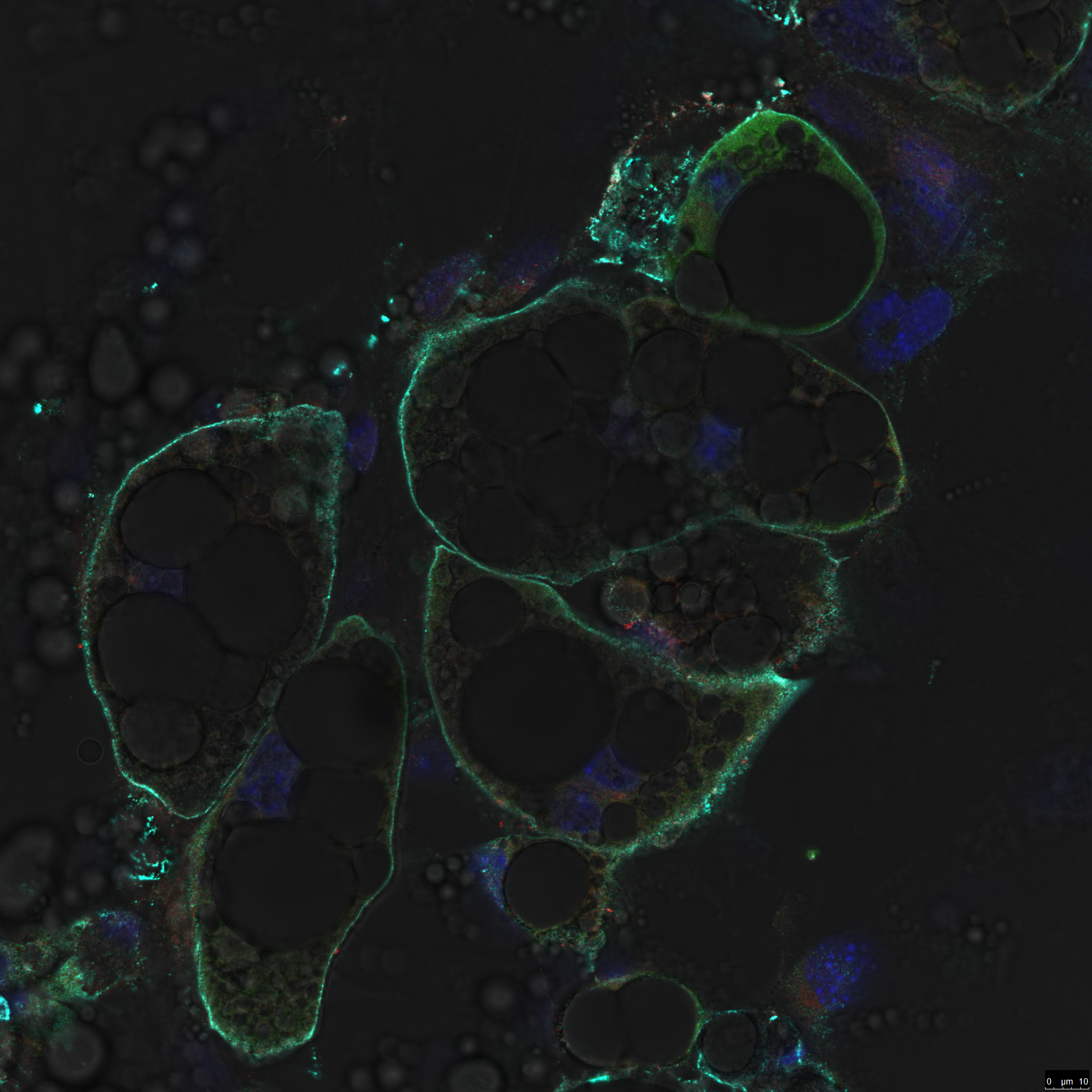

Supplement: Supplementary file 10 — Figure EV3 Source Data [file 44318_2025_520_MOESM10_ESM.zip › EV3/3B/WT_CPZ.tif]

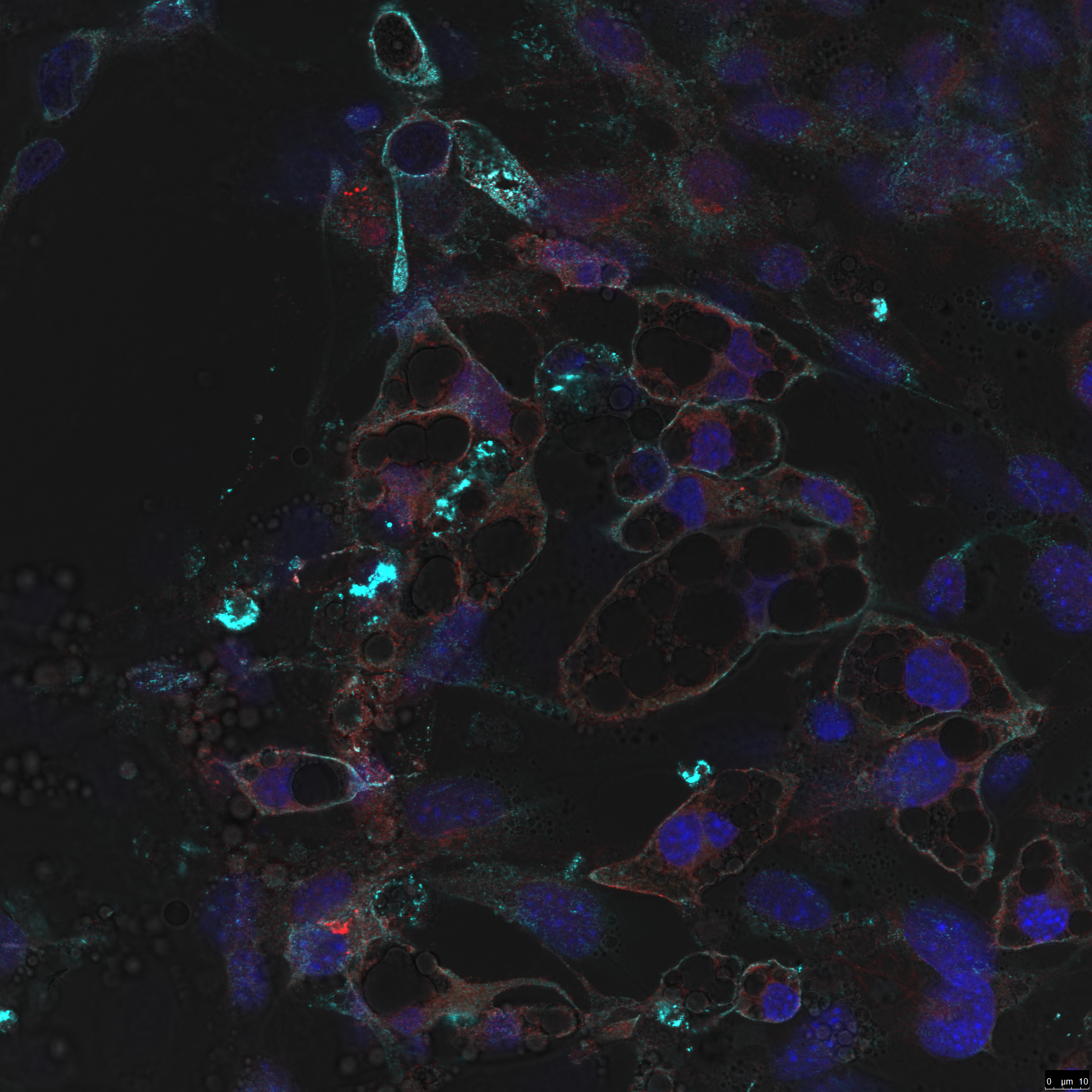

Supplement: Supplementary file 10 — Figure EV3 Source Data [file 44318_2025_520_MOESM10_ESM.zip › EV3/3B/KO_CPZ.tif]

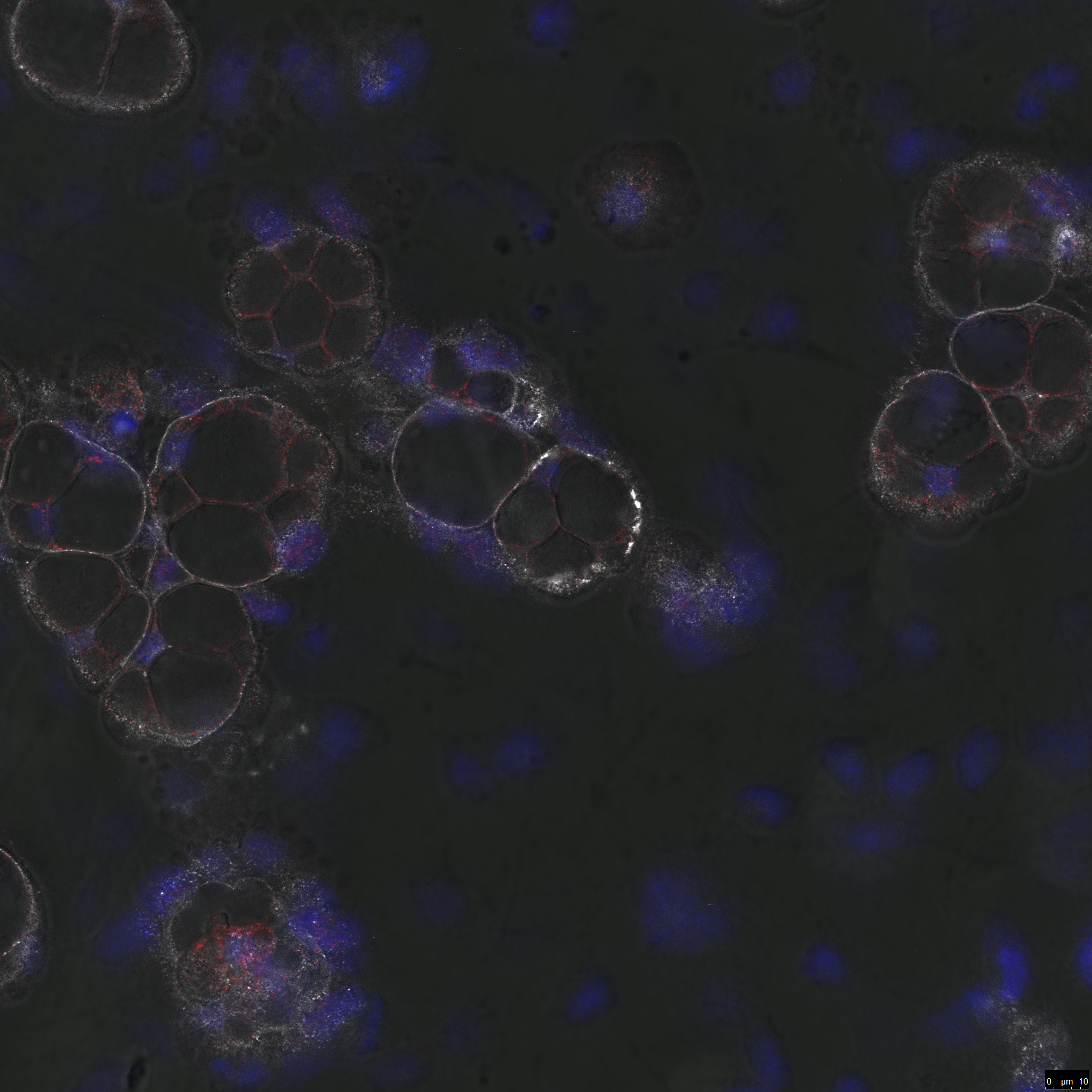

Supplement: Supplementary file 10 — Figure EV3 Source Data [file 44318_2025_520_MOESM10_ESM.zip › EV3/3B/KO_vehicle.tif]

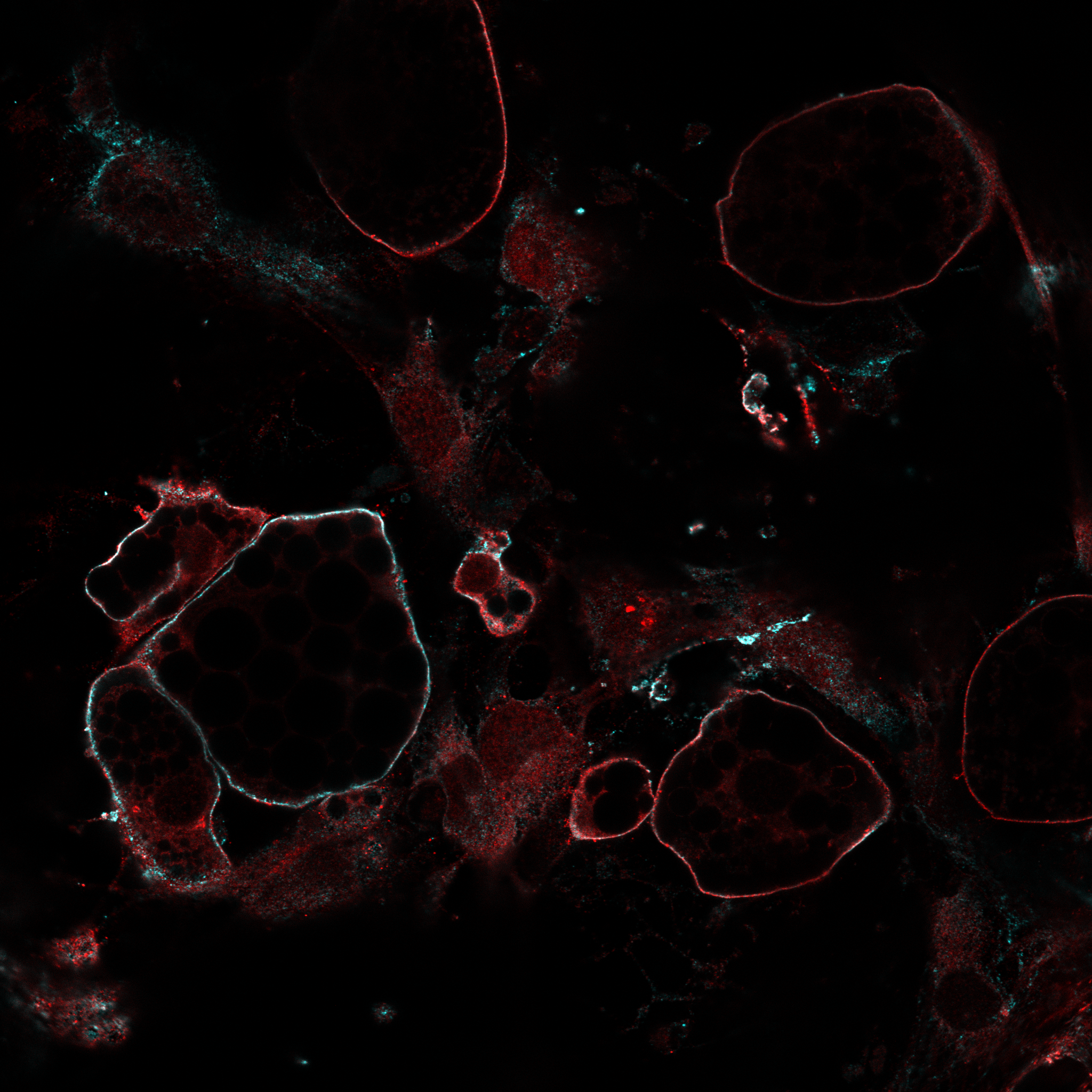

Supplement: Supplementary file 10 — Figure EV3 Source Data [file 44318_2025_520_MOESM10_ESM.zip › EV3/3B/WT_vehicle.tif]

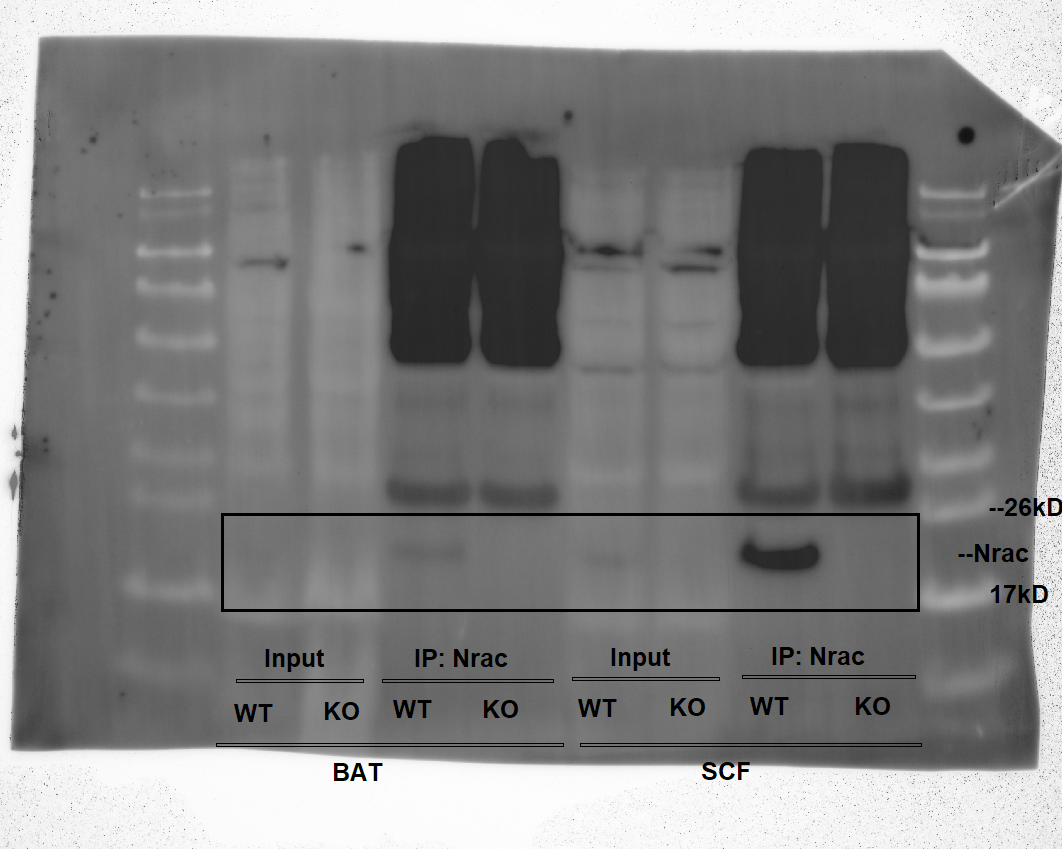

Supplement: Supplementary file 10 — Figure EV3 Source Data [file 44318_2025_520_MOESM10_ESM.zip › EV3/3A/NRAC.tif]

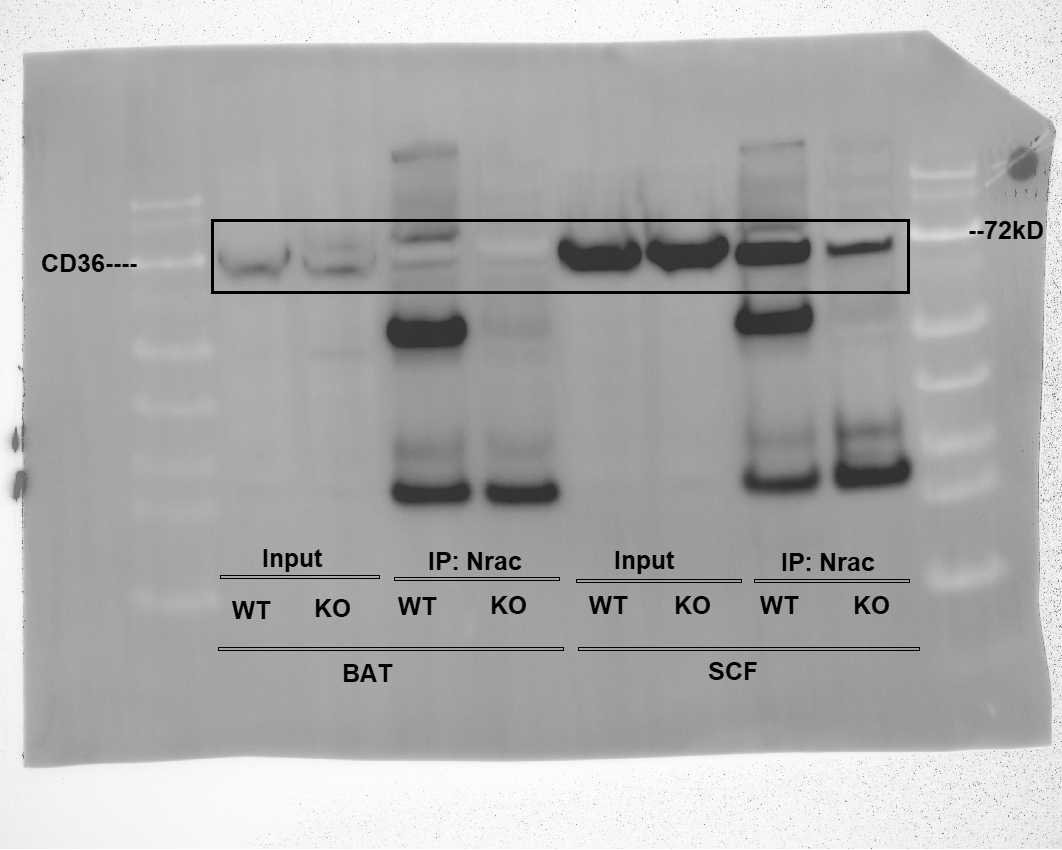

Supplement: Supplementary file 10 — Figure EV3 Source Data [file 44318_2025_520_MOESM10_ESM.zip › EV3/3A/CD36.tif]

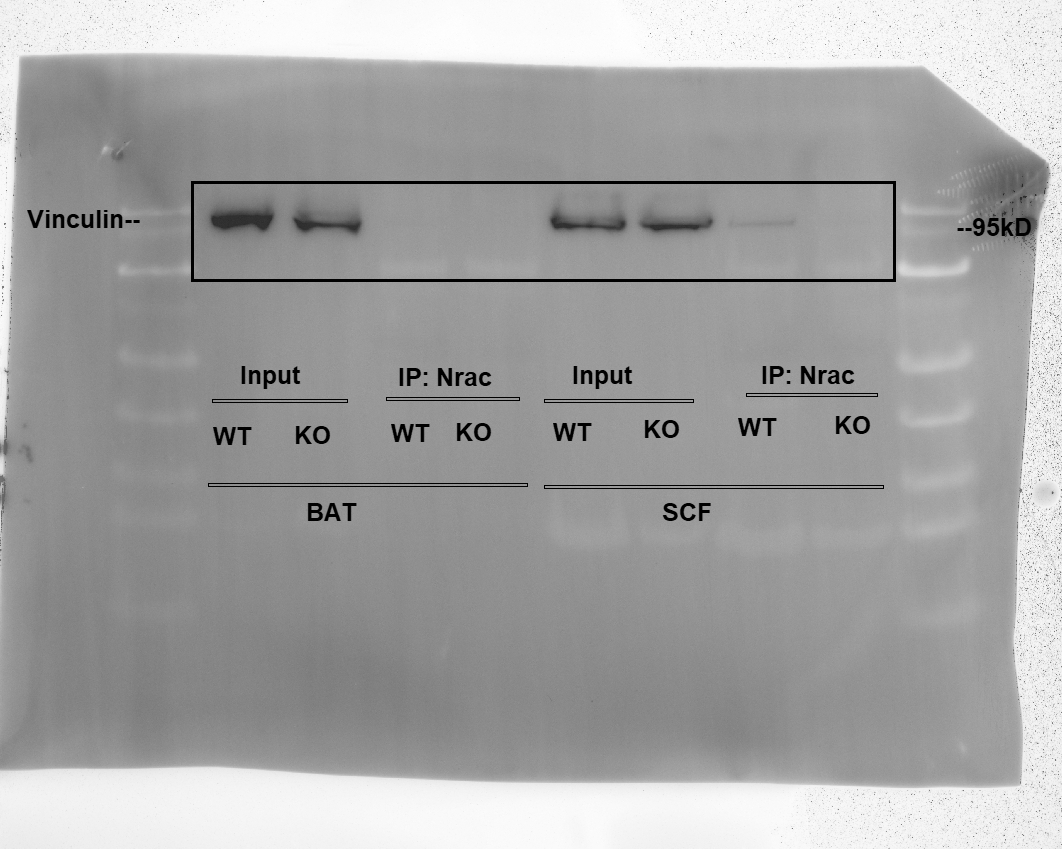

Supplement: Supplementary file 10 — Figure EV3 Source Data [file 44318_2025_520_MOESM10_ESM.zip › EV3/3A/VINCULIN.tif]

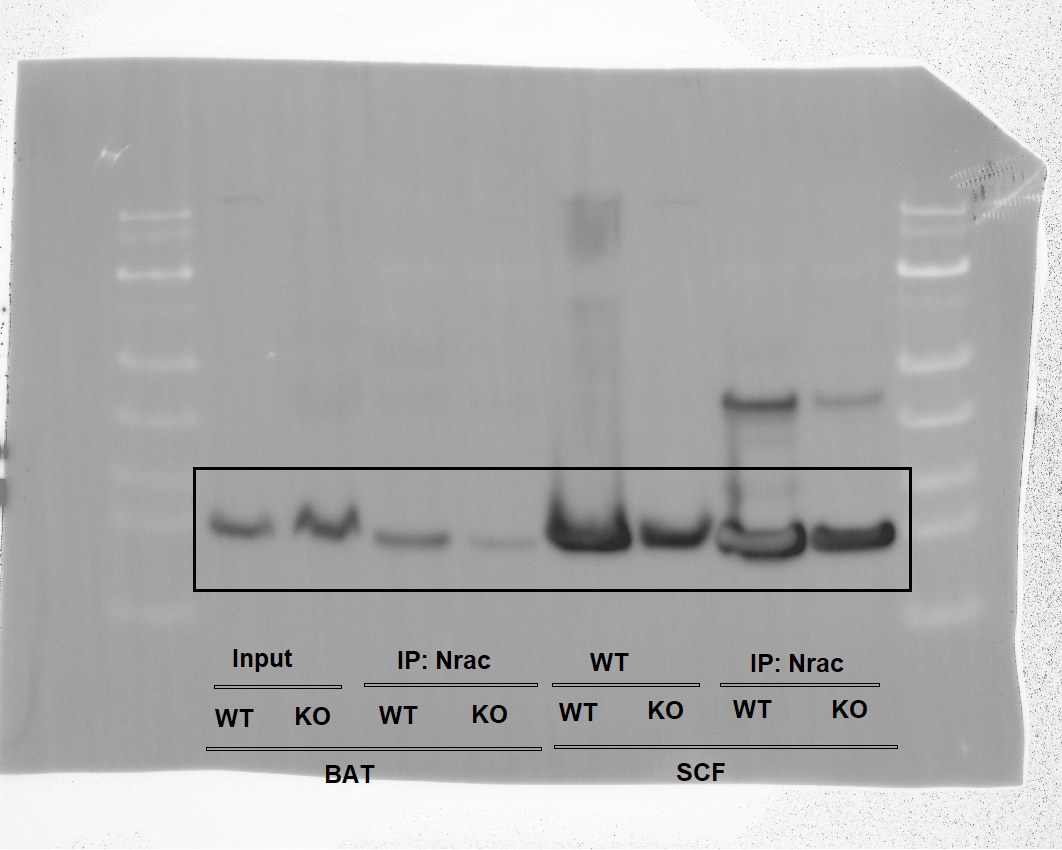

Supplement: Supplementary file 10 — Figure EV3 Source Data [file 44318_2025_520_MOESM10_ESM.zip › EV3/3A/CAV1.tif]

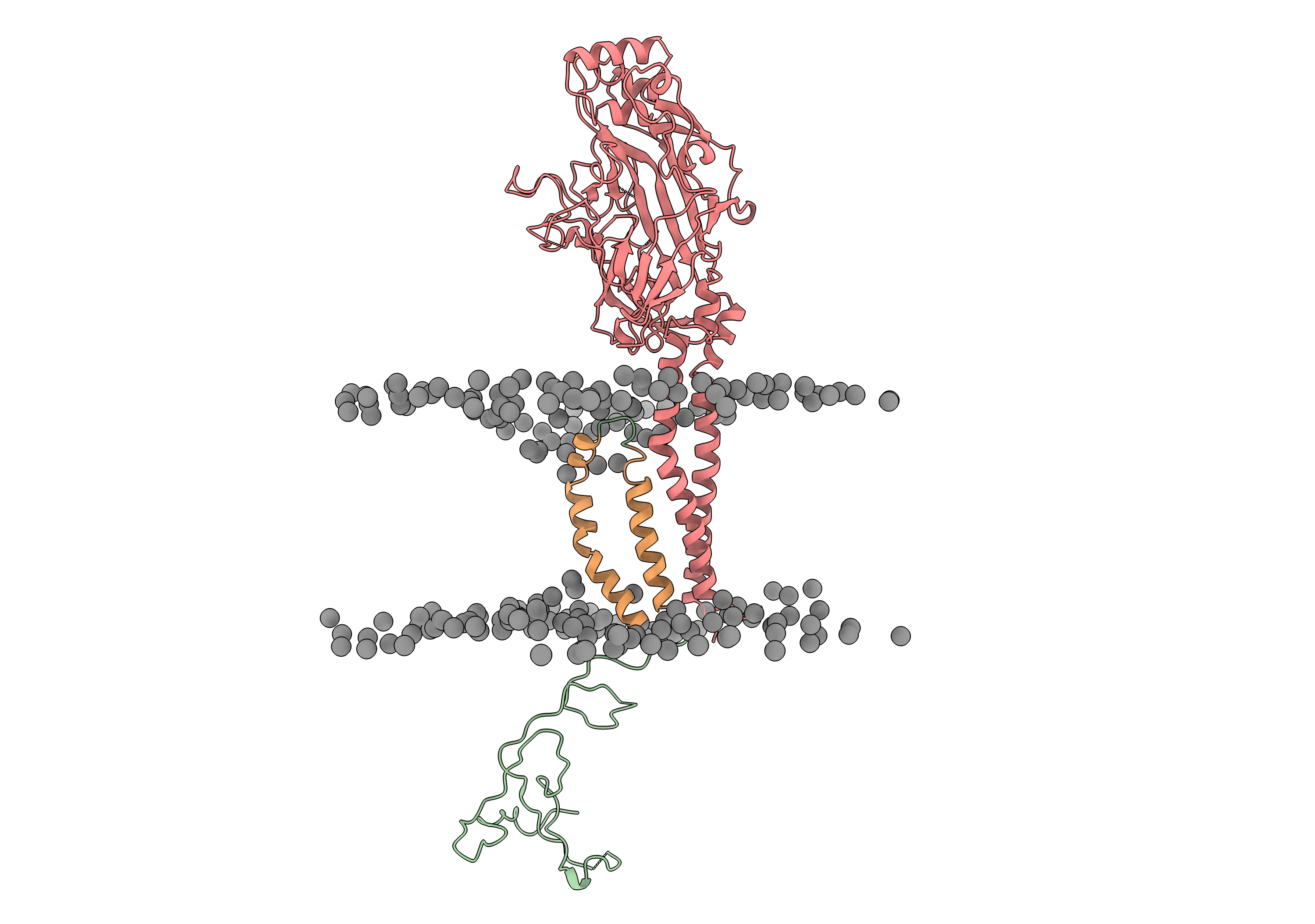

Supplement: Supplementary file 11 — Figure EV4 Source Data [file 44318_2025_520_MOESM11_ESM.zip › EV4/4A-C/MD Simulations/untitled folder/protein_membrane_zoom_3I.png]

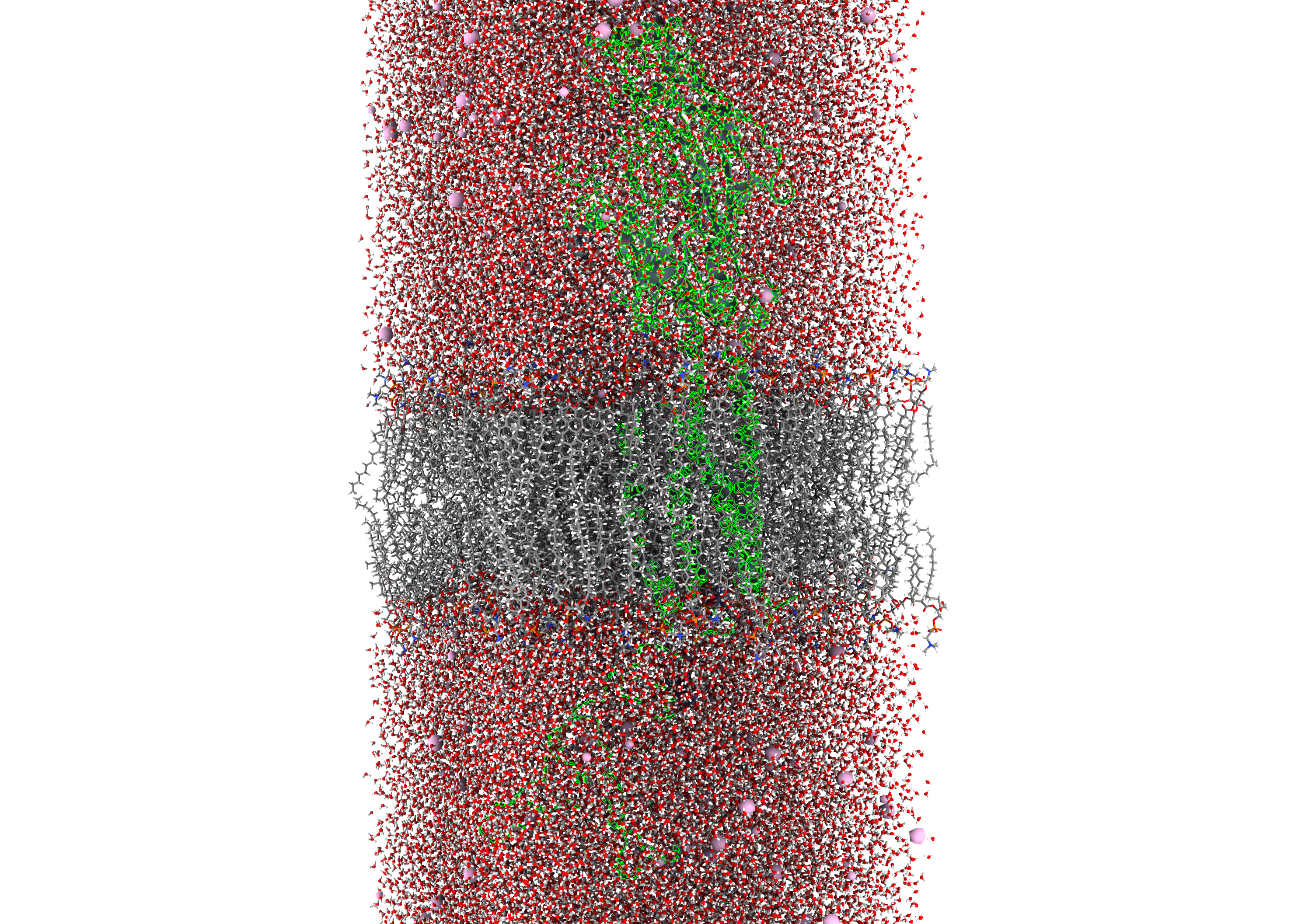

Supplement: Supplementary file 11 — Figure EV4 Source Data [file 44318_2025_520_MOESM11_ESM.zip › EV4/4A-C/MD Simulations/untitled folder/full_zoom_1.png]

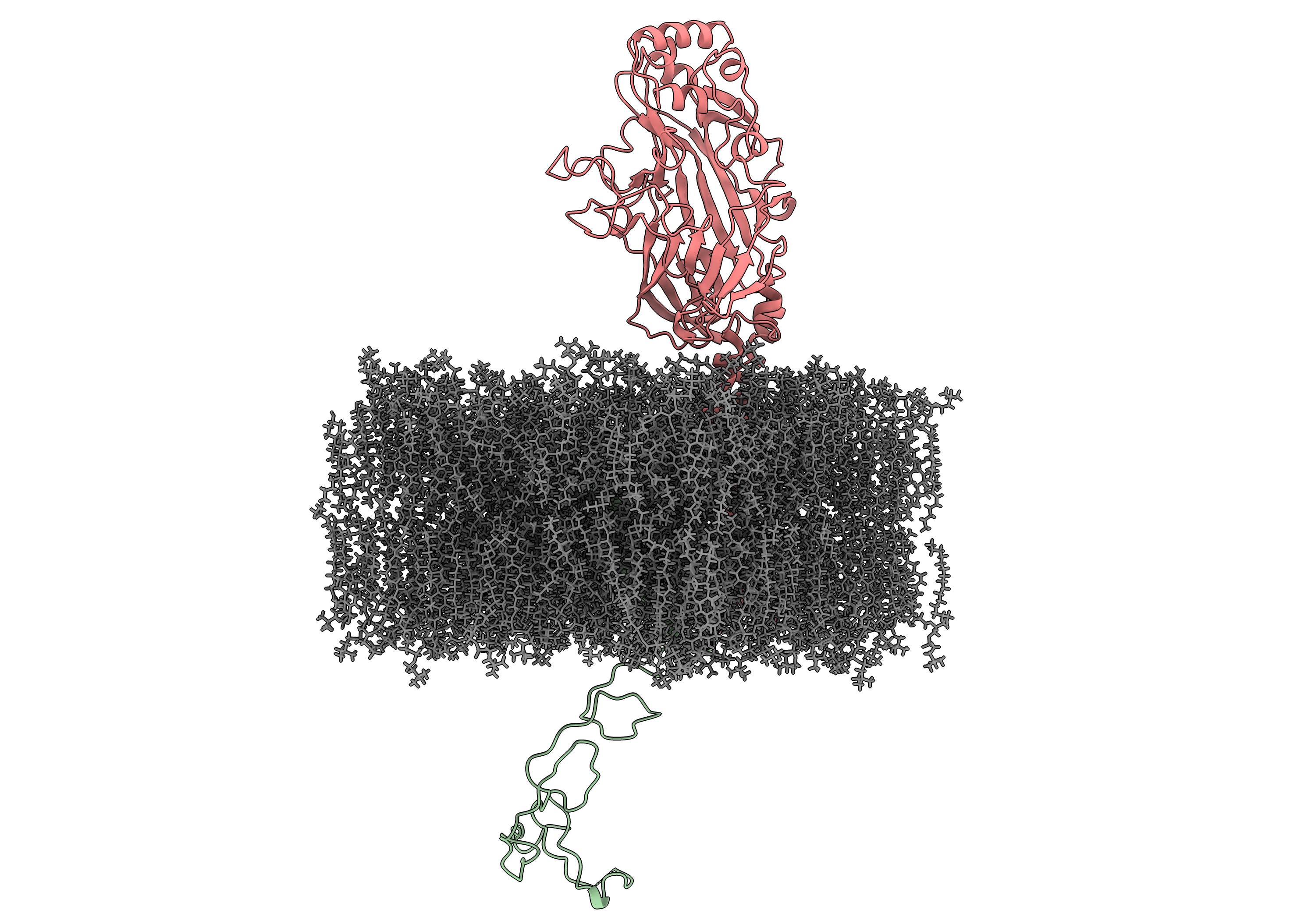

Supplement: Supplementary file 11 — Figure EV4 Source Data [file 44318_2025_520_MOESM11_ESM.zip › EV4/4A-C/MD Simulations/untitled folder/protein_membrane_zoom_1C.png]

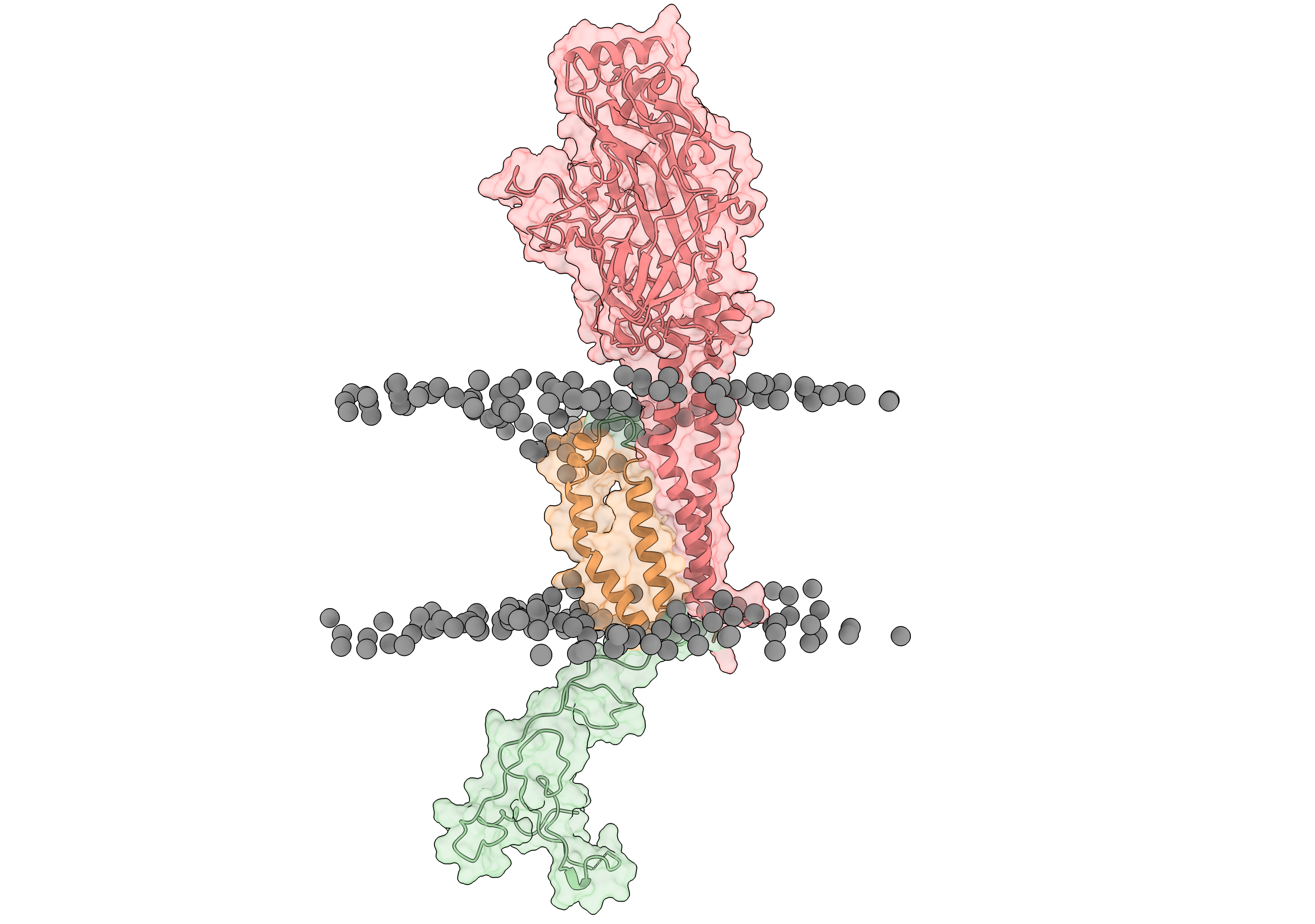

Supplement: Supplementary file 11 — Figure EV4 Source Data [file 44318_2025_520_MOESM11_ESM.zip › EV4/4A-C/MD Simulations/untitled folder/protein_membrane_zoom_3F.png]

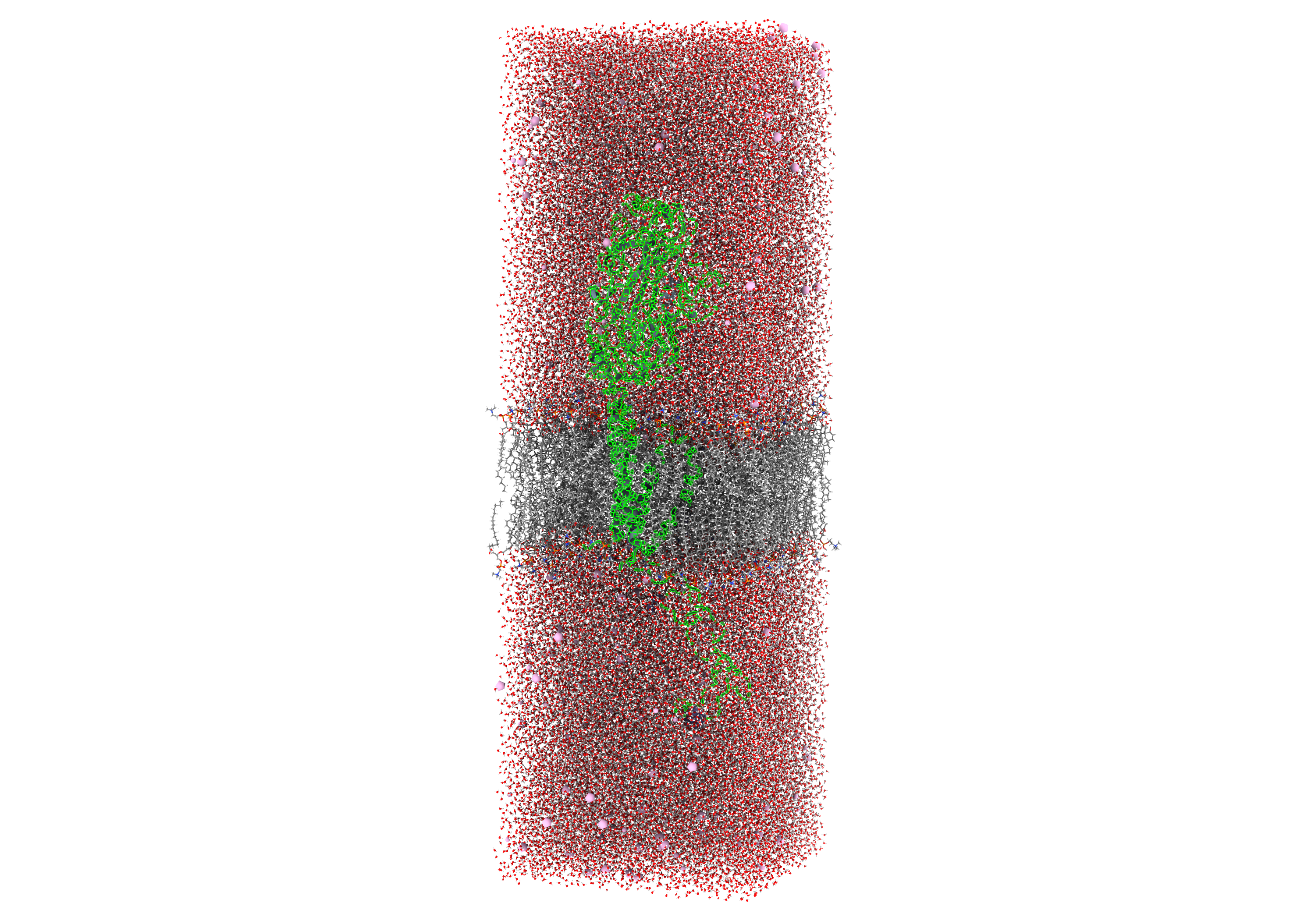

Supplement: Supplementary file 11 — Figure EV4 Source Data [file 44318_2025_520_MOESM11_ESM.zip › EV4/4A-C/MD Simulations/untitled folder/full_1.png]

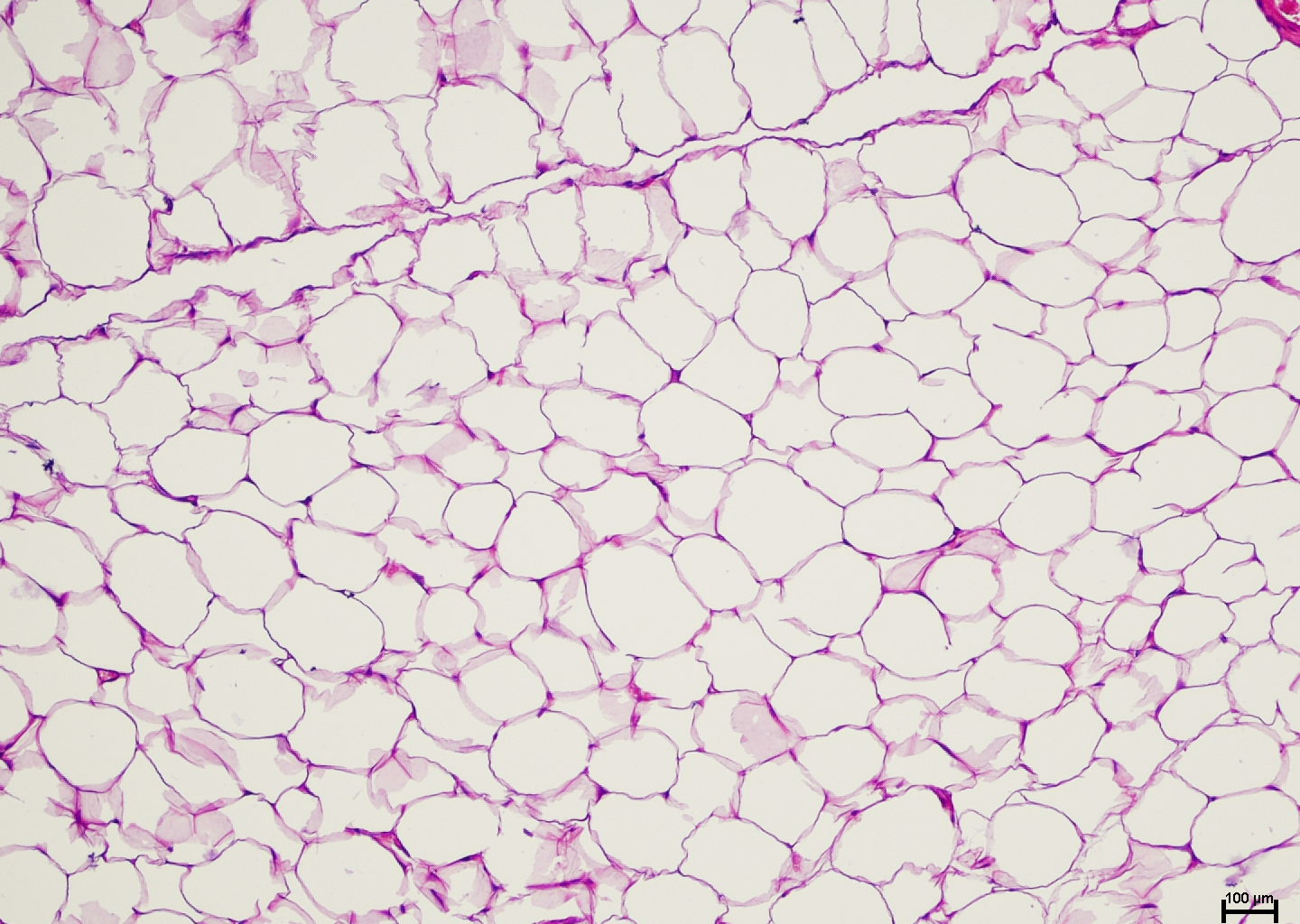

Supplement: Supplementary file 12 — Figure EV5 Source Data [file 44318_2025_520_MOESM12_ESM.zip › EV5/5M/PGF_KO.tif]

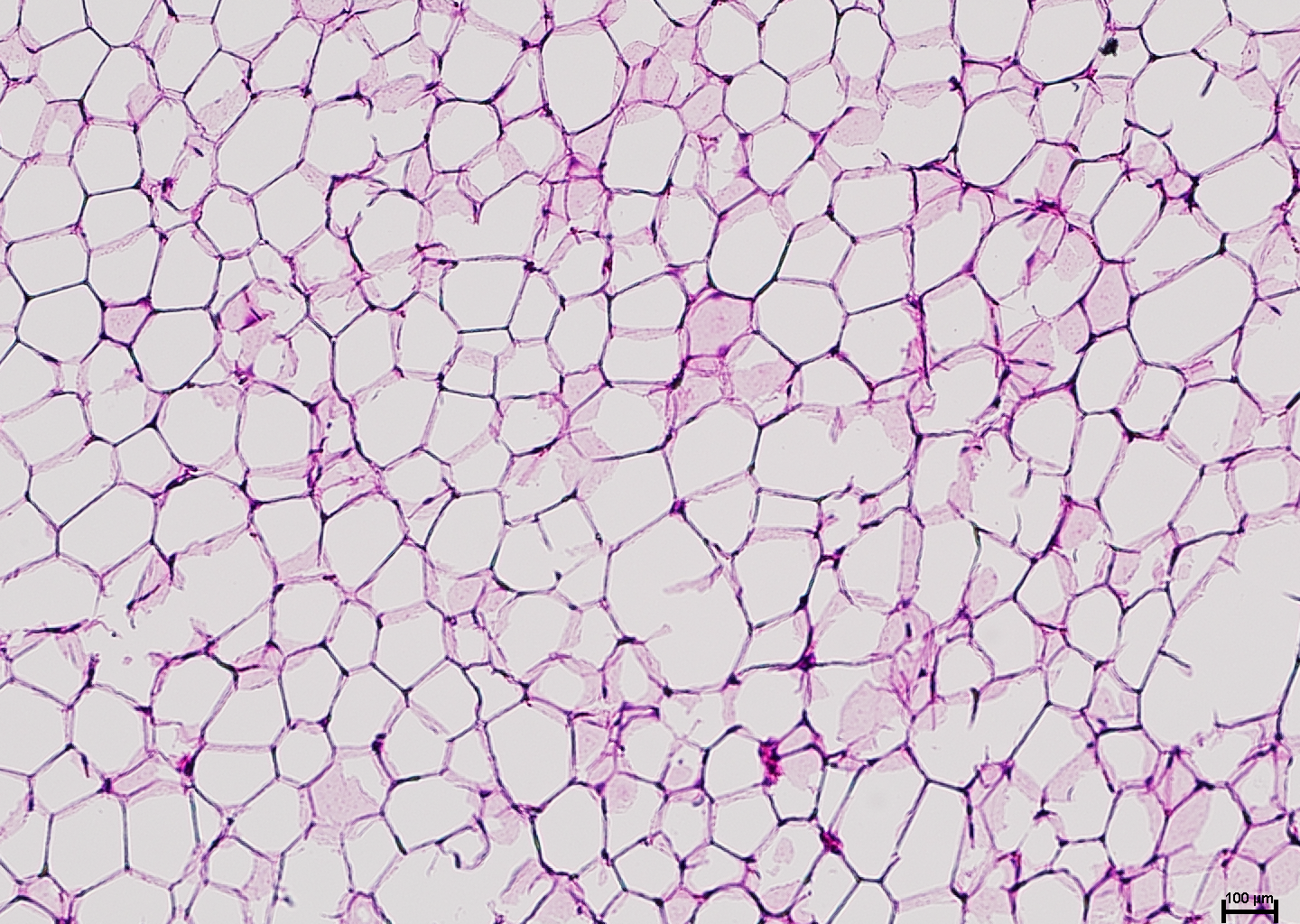

Supplement: Supplementary file 12 — Figure EV5 Source Data [file 44318_2025_520_MOESM12_ESM.zip › EV5/5M/SCF_WT.tif]

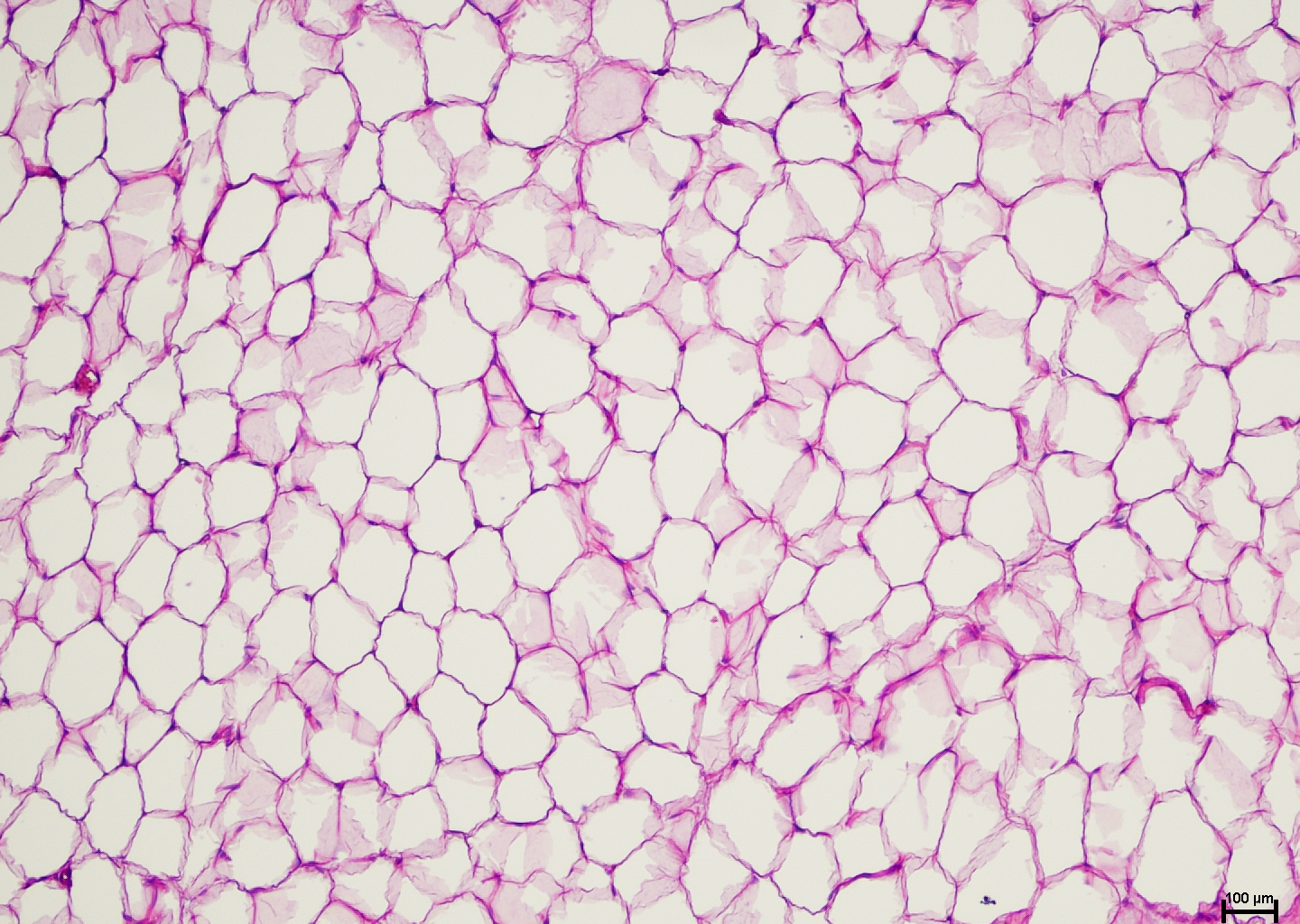

Supplement: Supplementary file 12 — Figure EV5 Source Data [file 44318_2025_520_MOESM12_ESM.zip › EV5/5M/PGF_WT.tif]

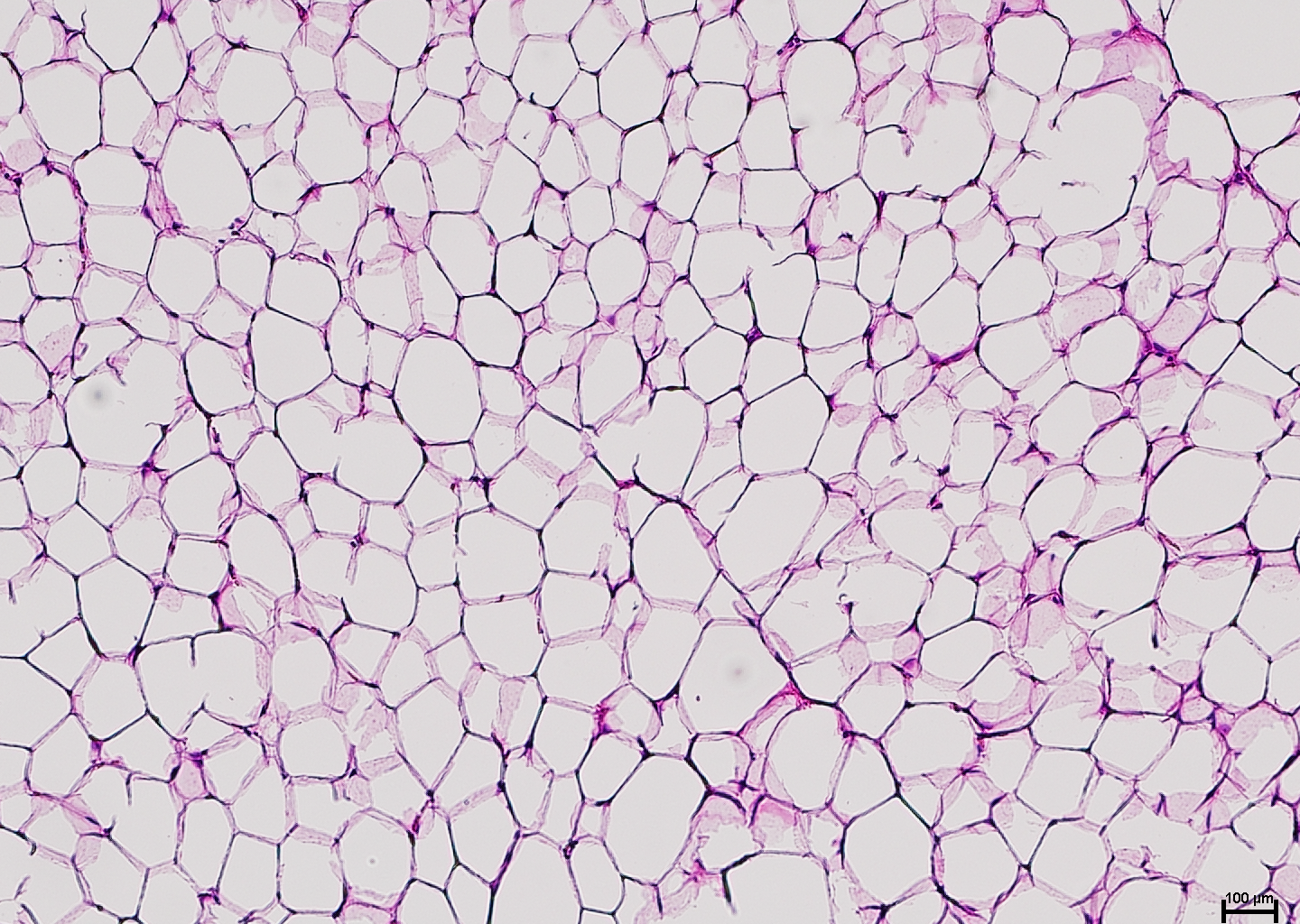

Supplement: Supplementary file 12 — Figure EV5 Source Data [file 44318_2025_520_MOESM12_ESM.zip › EV5/5M/SCF_KO.tif]

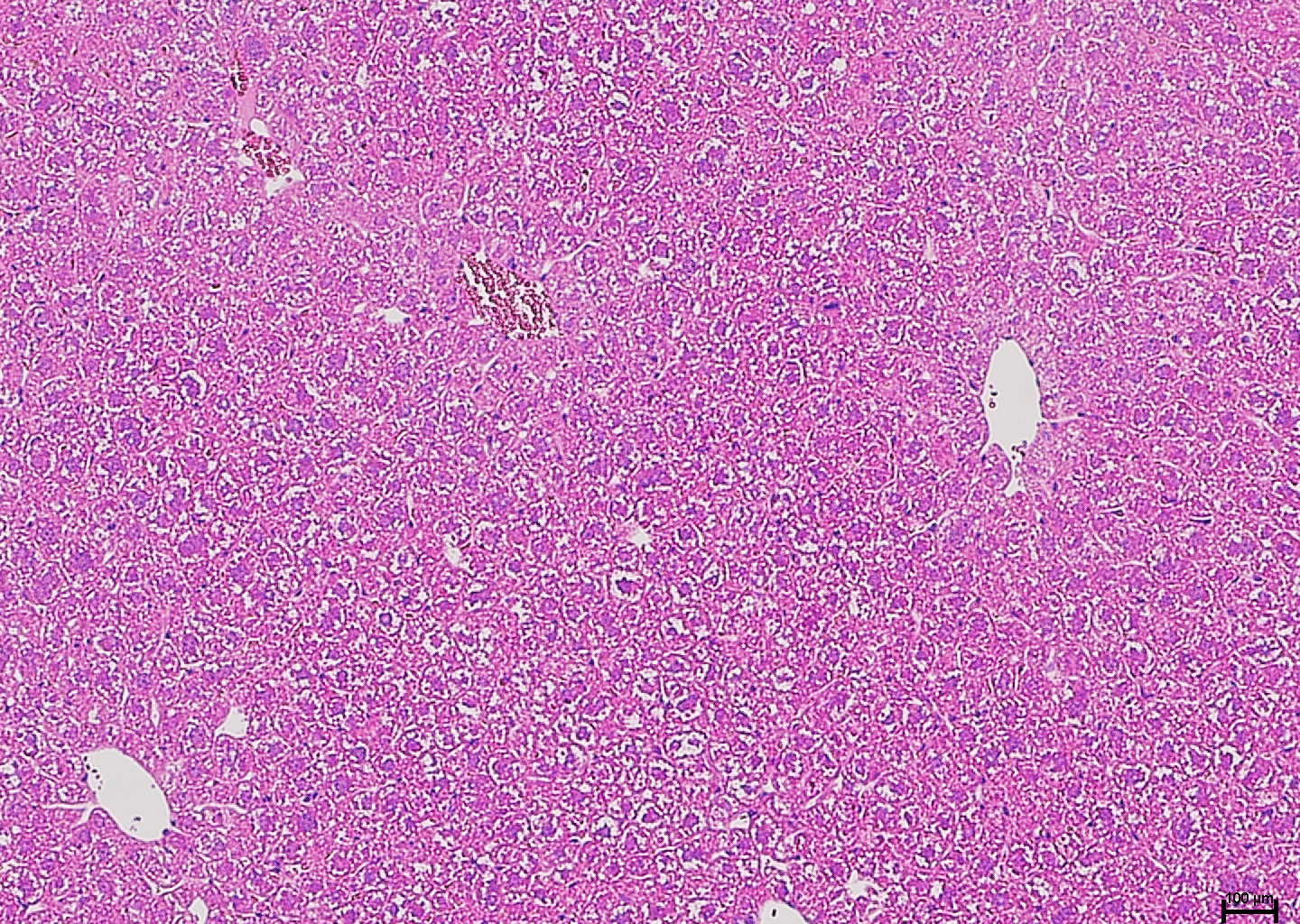

Supplement: Supplementary file 12 — Figure EV5 Source Data [file 44318_2025_520_MOESM12_ESM.zip › EV5/5D/Liver_WT.tif]

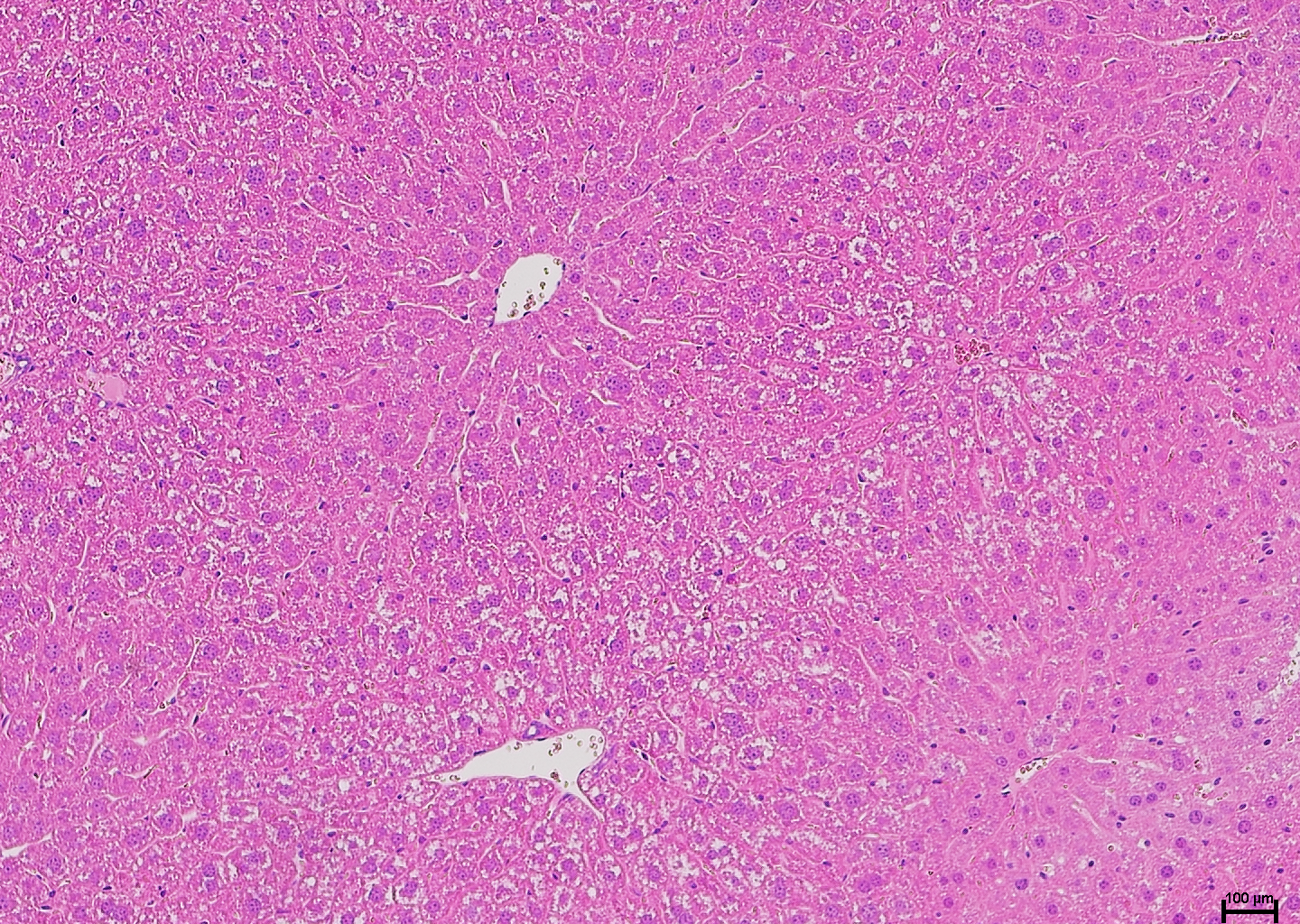

Supplement: Supplementary file 12 — Figure EV5 Source Data [file 44318_2025_520_MOESM12_ESM.zip › EV5/5D/Liver_KO.tif]

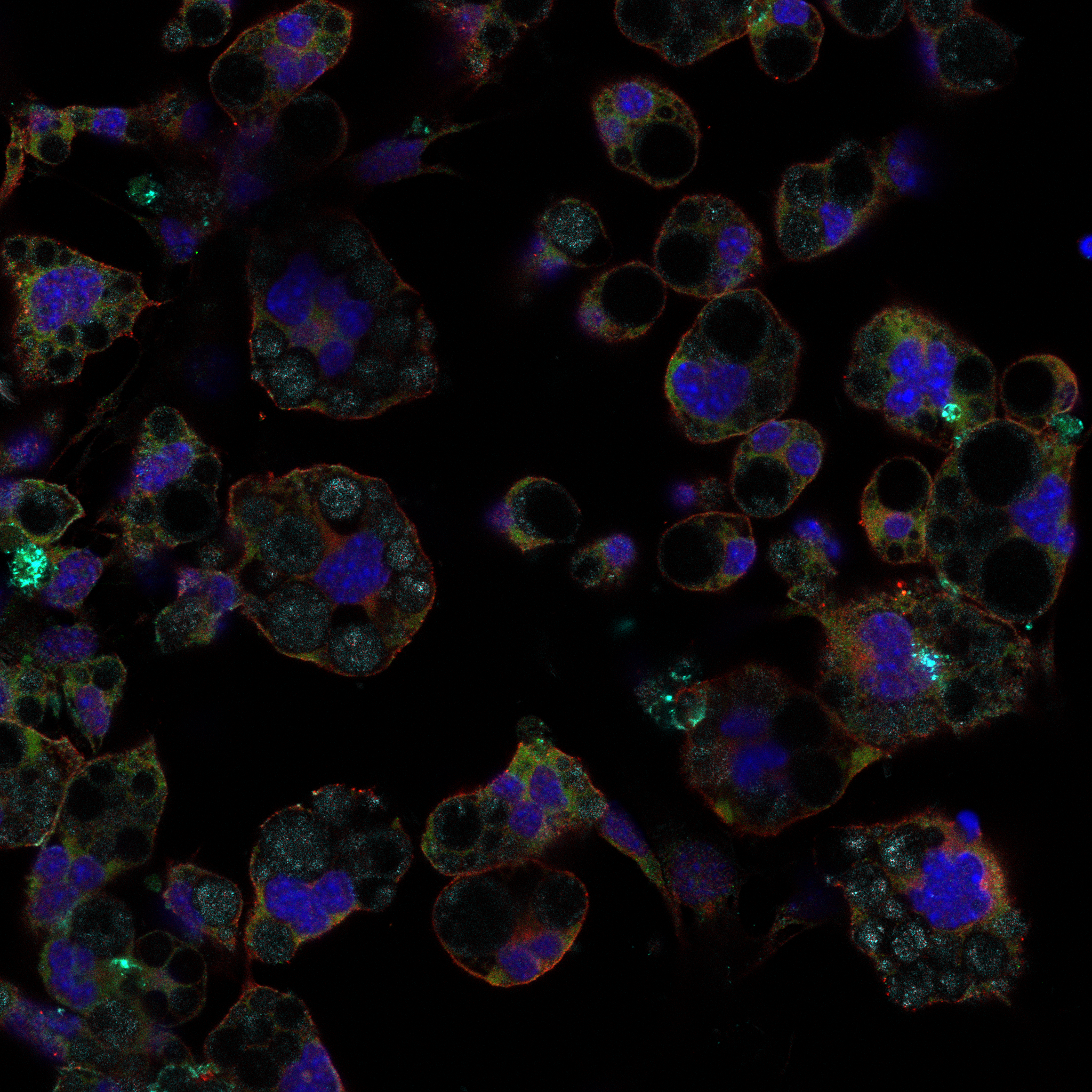

Supplement: Supplementary file 12 — Figure EV5 Source Data [file 44318_2025_520_MOESM12_ESM.zip › EV5/5Q/Oleate+CPZ_WT.tif]

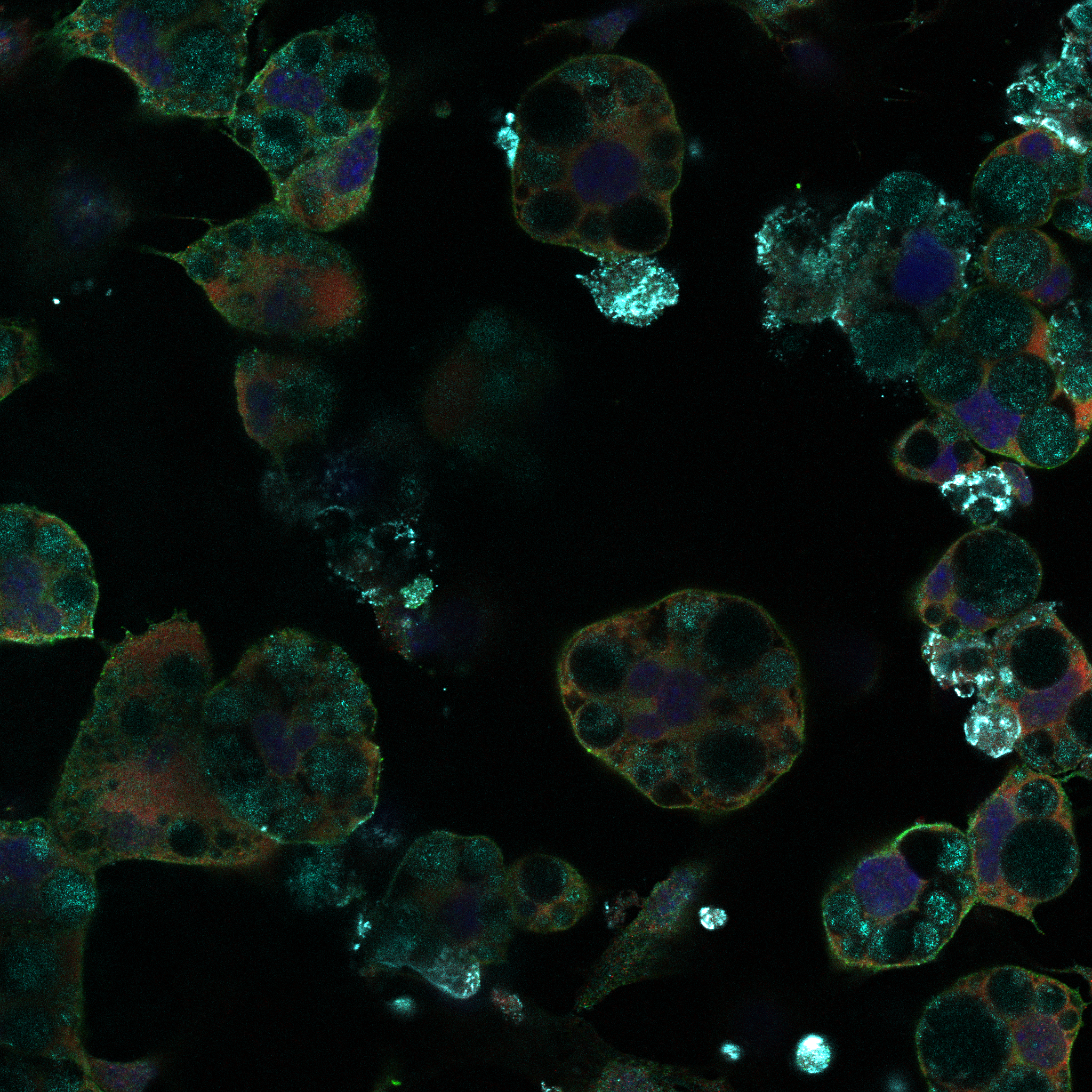

Supplement: Supplementary file 12 — Figure EV5 Source Data [file 44318_2025_520_MOESM12_ESM.zip › EV5/5Q/Oleate_WT.tif]

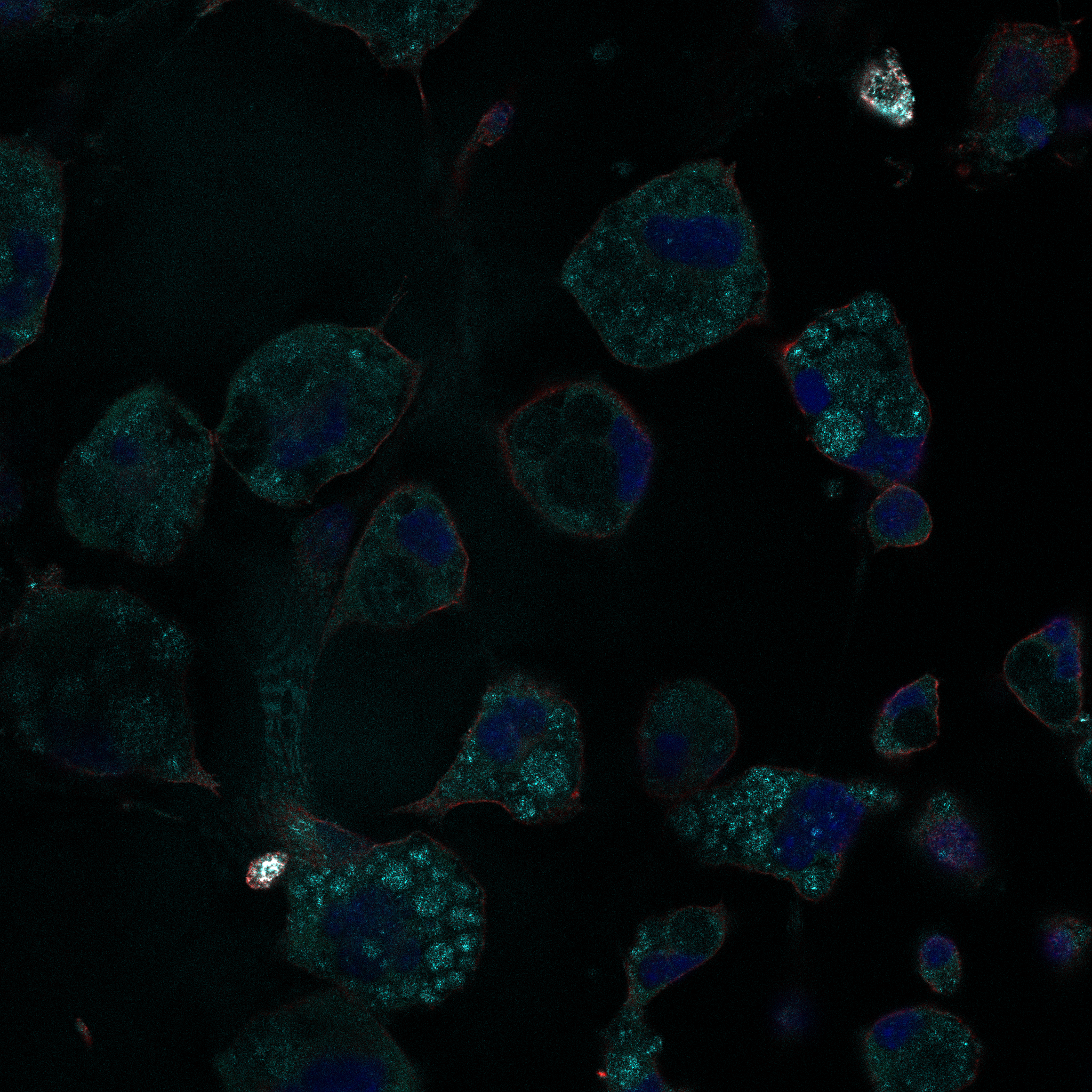

Supplement: Supplementary file 12 — Figure EV5 Source Data [file 44318_2025_520_MOESM12_ESM.zip › EV5/5Q/Oleate+ CPZ_KO.tif]

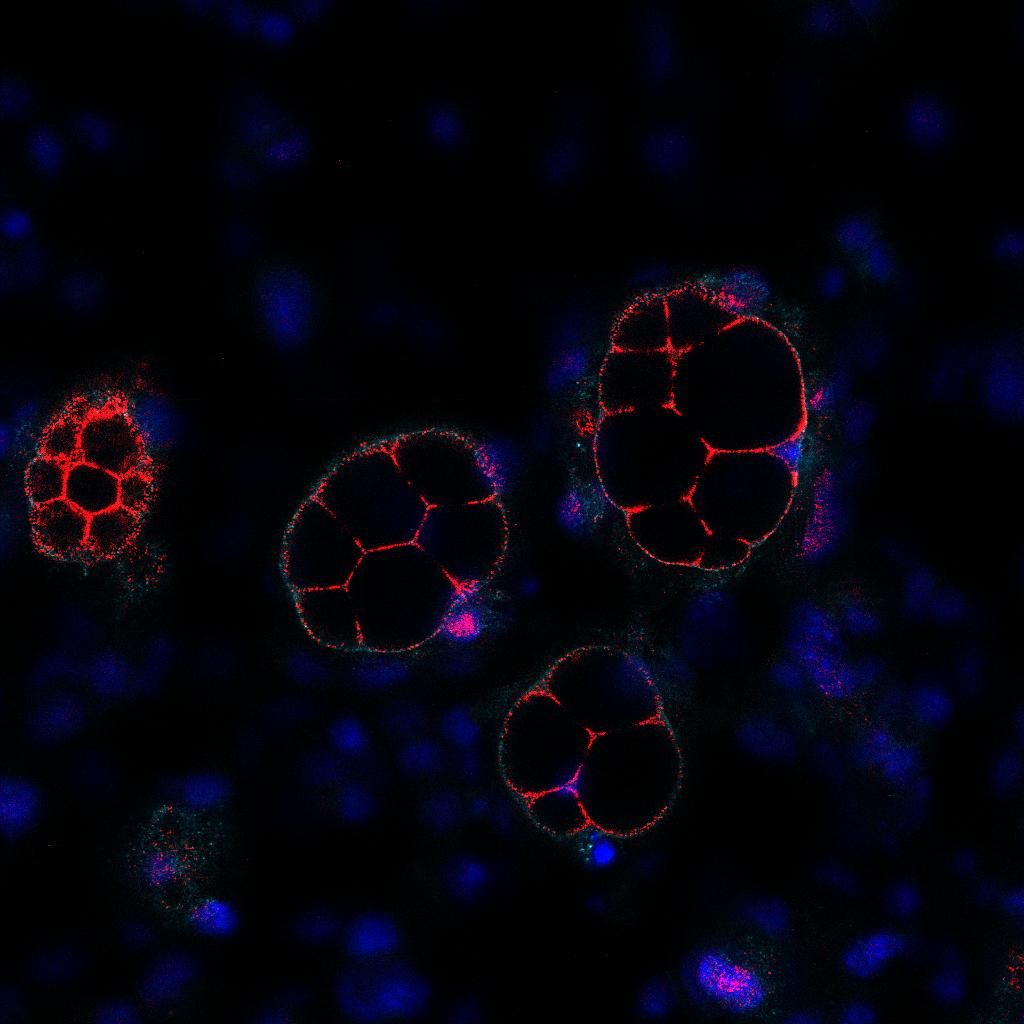

Supplement: Supplementary file 12 — Figure EV5 Source Data [file 44318_2025_520_MOESM12_ESM.zip › EV5/5Q/BSA_KO.tif]

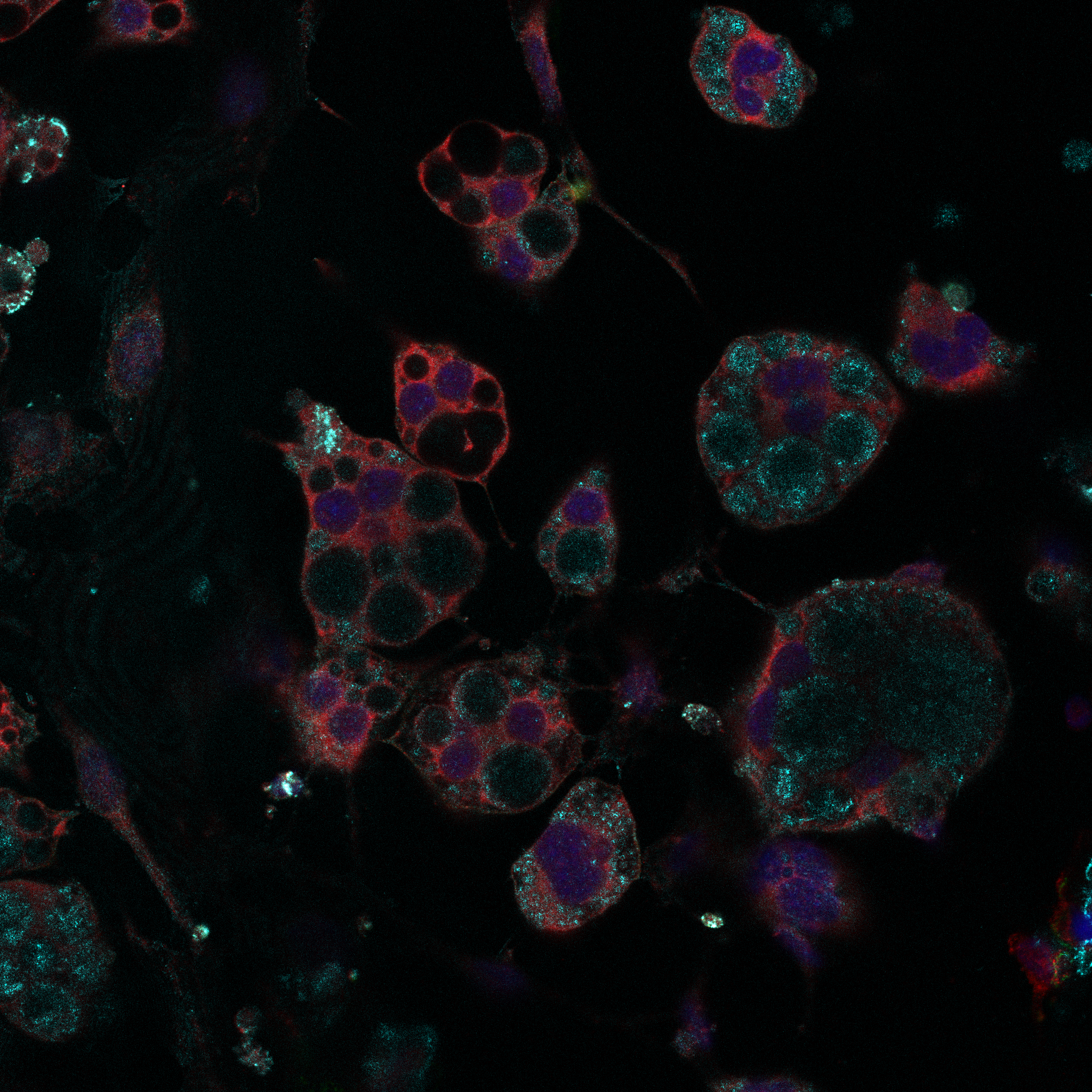

Supplement: Supplementary file 12 — Figure EV5 Source Data [file 44318_2025_520_MOESM12_ESM.zip › EV5/5Q/KO_Oleate.tif]

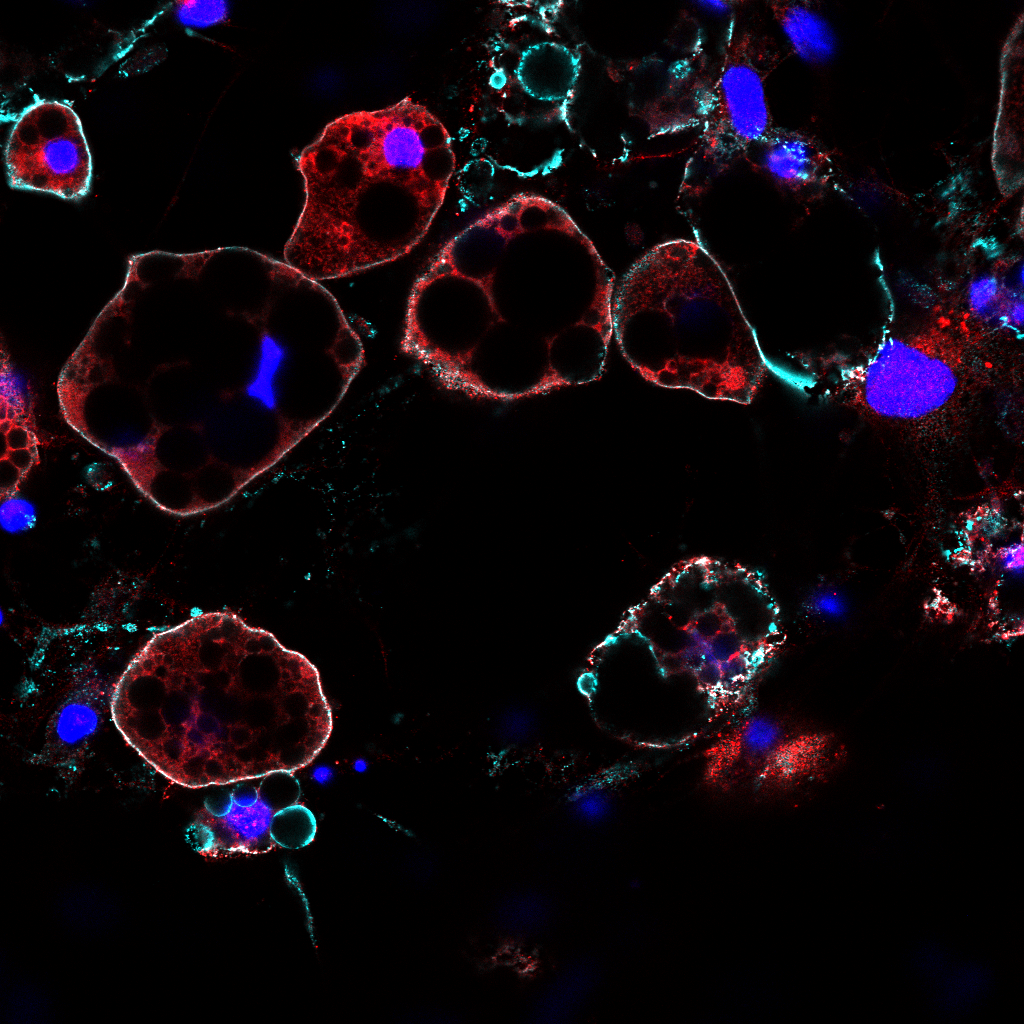

Supplement: Supplementary file 12 — Figure EV5 Source Data [file 44318_2025_520_MOESM12_ESM.zip › EV5/5Q/BSA_WT.tif]

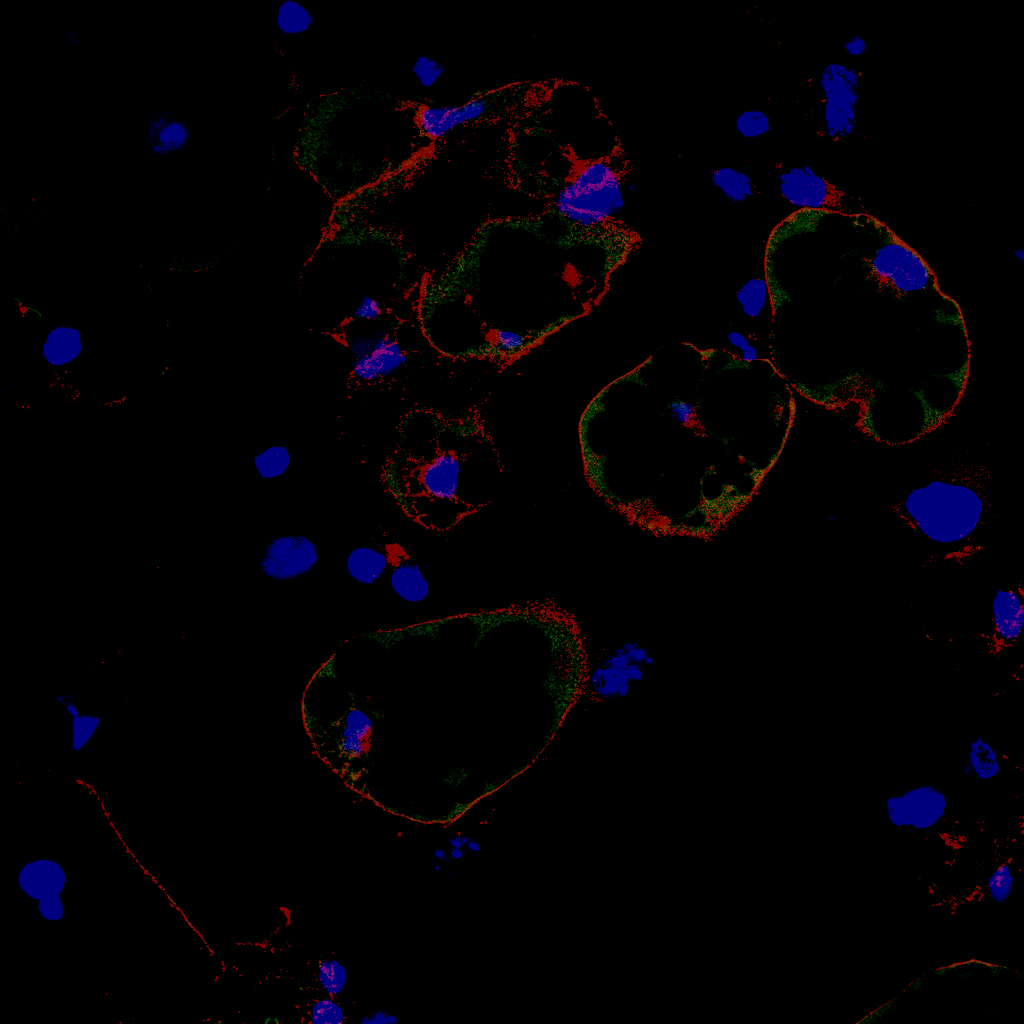

Supplement: Supplementary file 12 — Figure EV5 Source Data [file 44318_2025_520_MOESM12_ESM.zip › EV5/5P/Project_IS035_Oleate 10-200uM_Series001_oleate 10uM.tif]

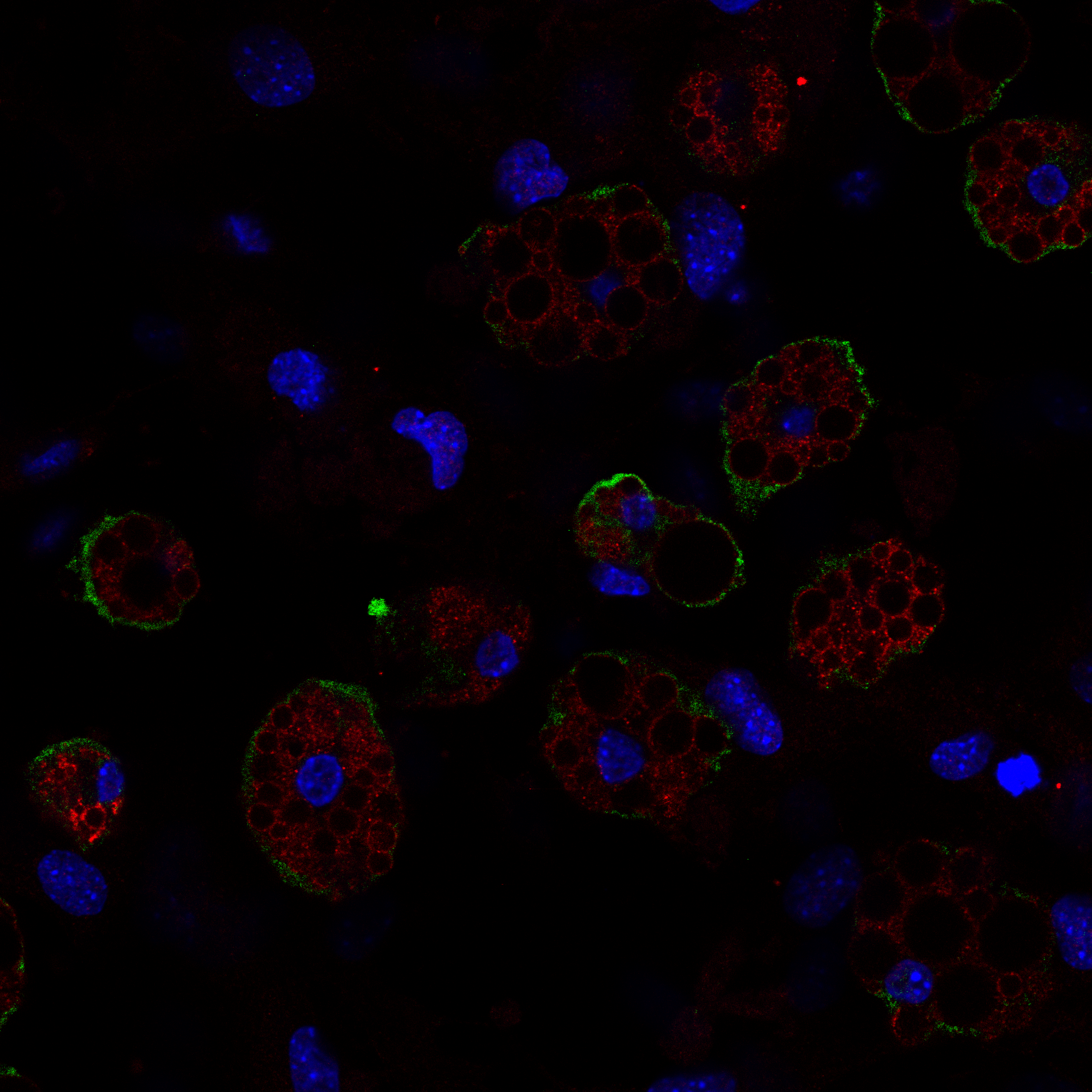

Supplement: Supplementary file 12 — Figure EV5 Source Data [file 44318_2025_520_MOESM12_ESM.zip › EV5/5P/Project_IS035_Oleate 10-200uM_Series009_oleate_200uM_merge.tif]

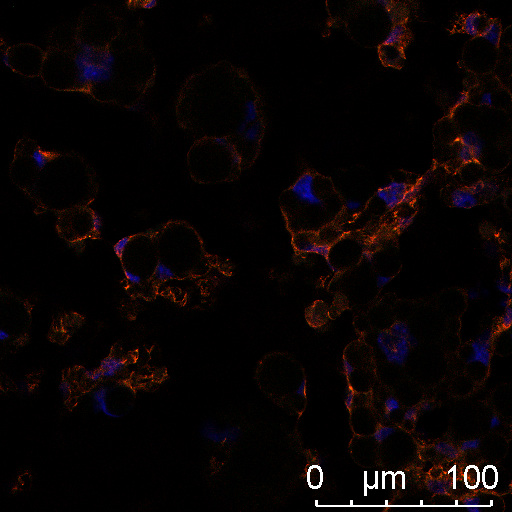

Supplement: Supplementary file 12 — Figure EV5 Source Data [file 44318_2025_520_MOESM12_ESM.zip › EV5/5P/Project_IS035_Oleate 10-200uM_Series002_oleate 10uM.tif]

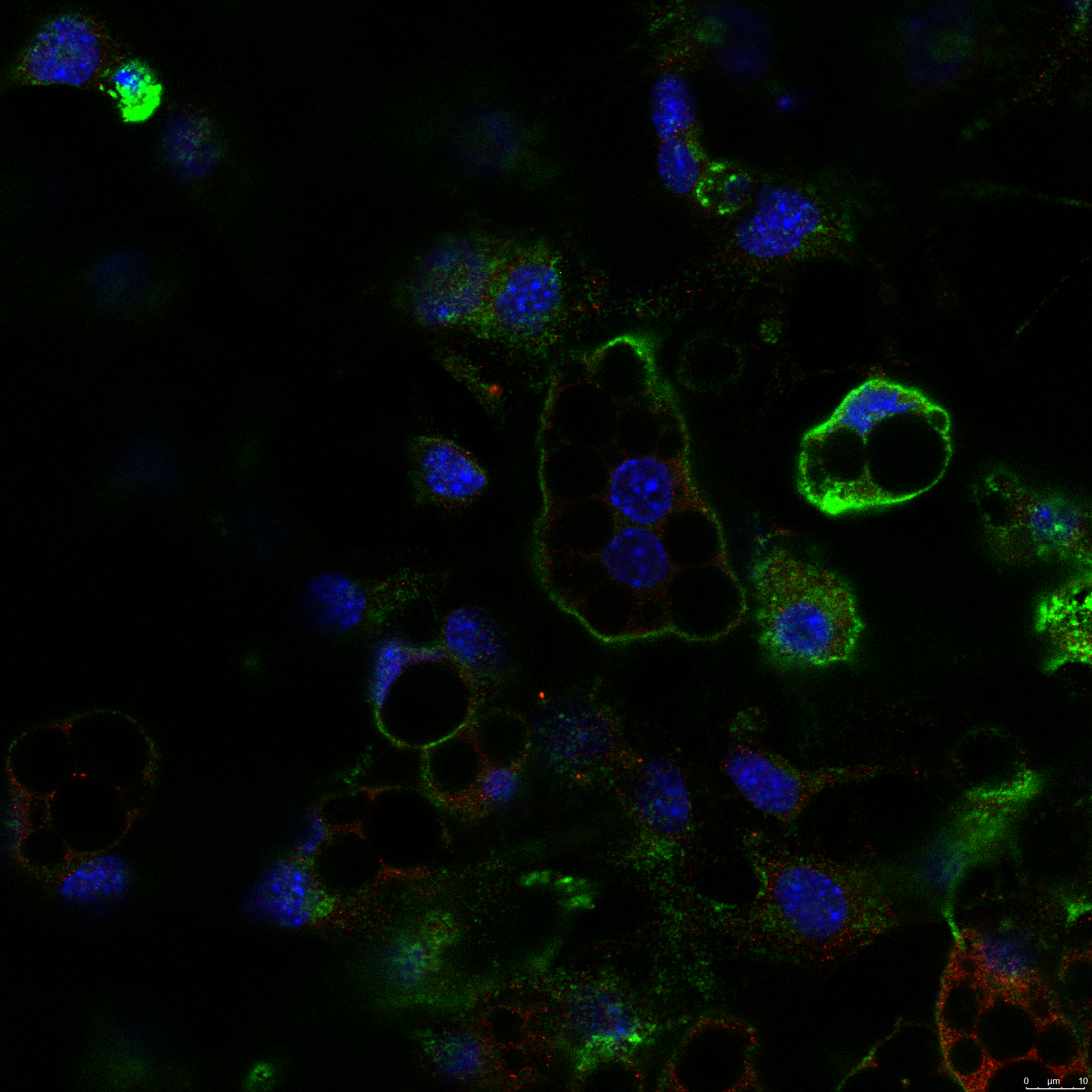

Supplement: Supplementary file 13 — Figure EV6 Source Data [file 44318_2025_520_MOESM13_ESM.zip › EV6/6A/Oleate_5min.tif]

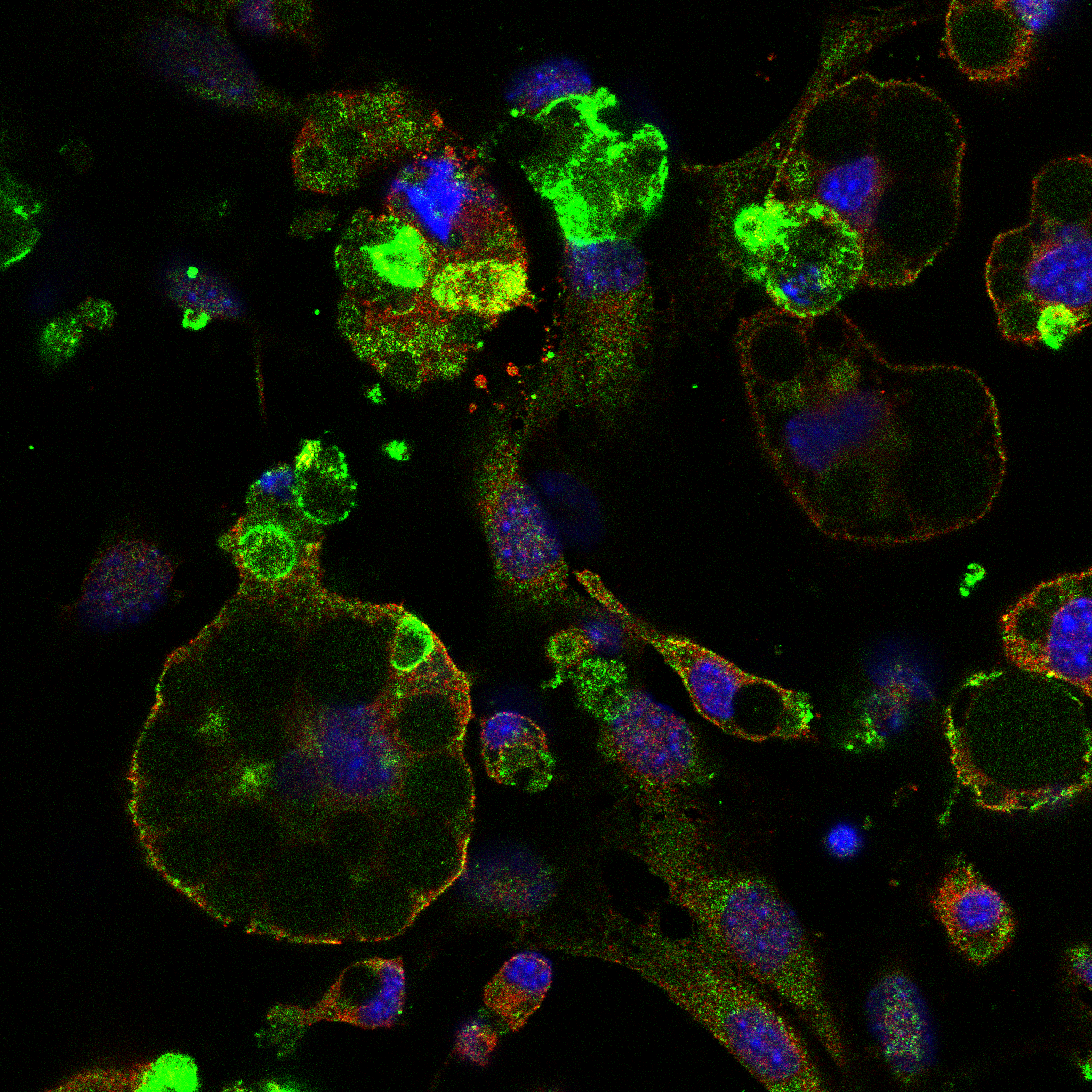

Supplement: Supplementary file 13 — Figure EV6 Source Data [file 44318_2025_520_MOESM13_ESM.zip › EV6/6A/BSA_5min.tif]

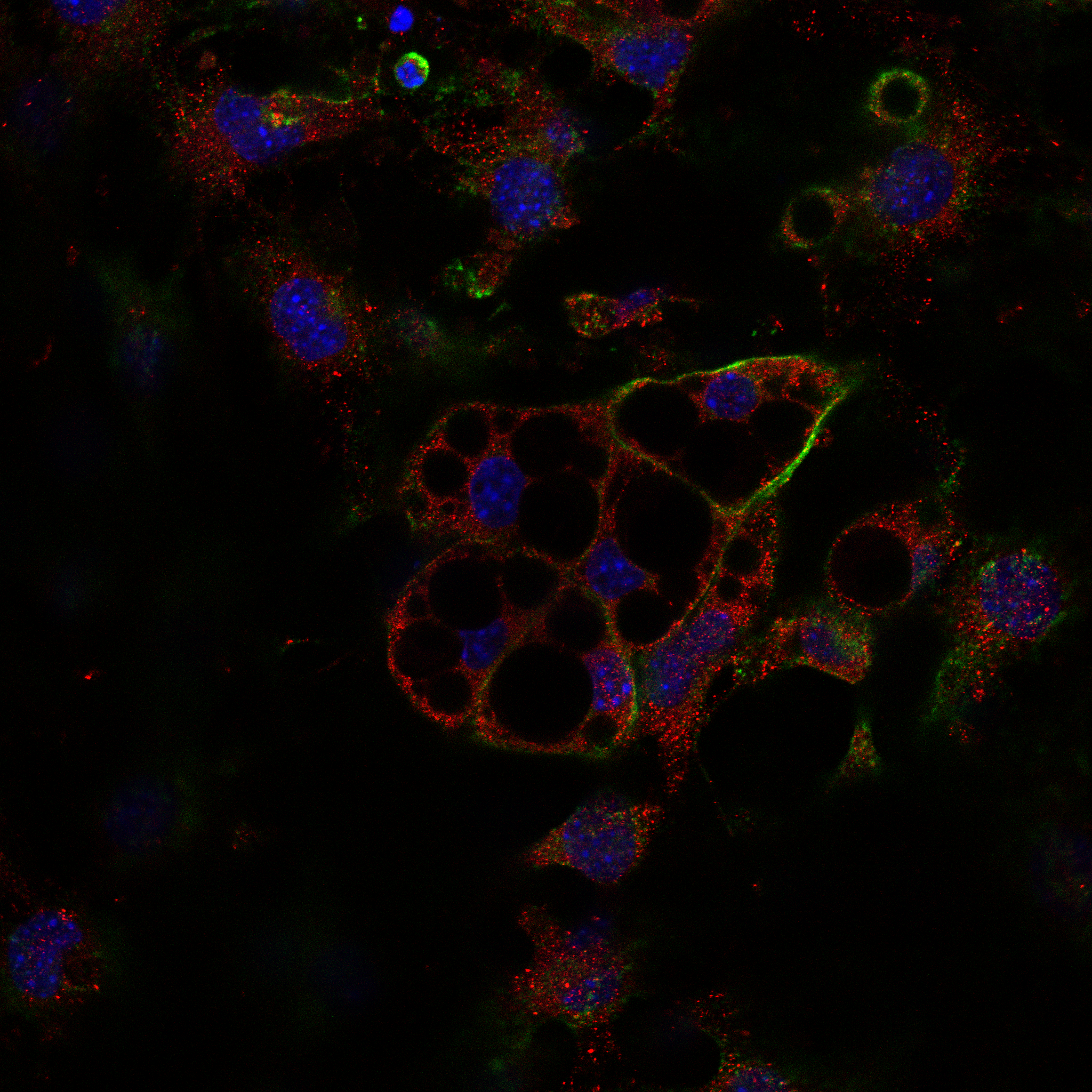

Supplement: Supplementary file 13 — Figure EV6 Source Data [file 44318_2025_520_MOESM13_ESM.zip › EV6/6A/Palmitate_5min.tif]

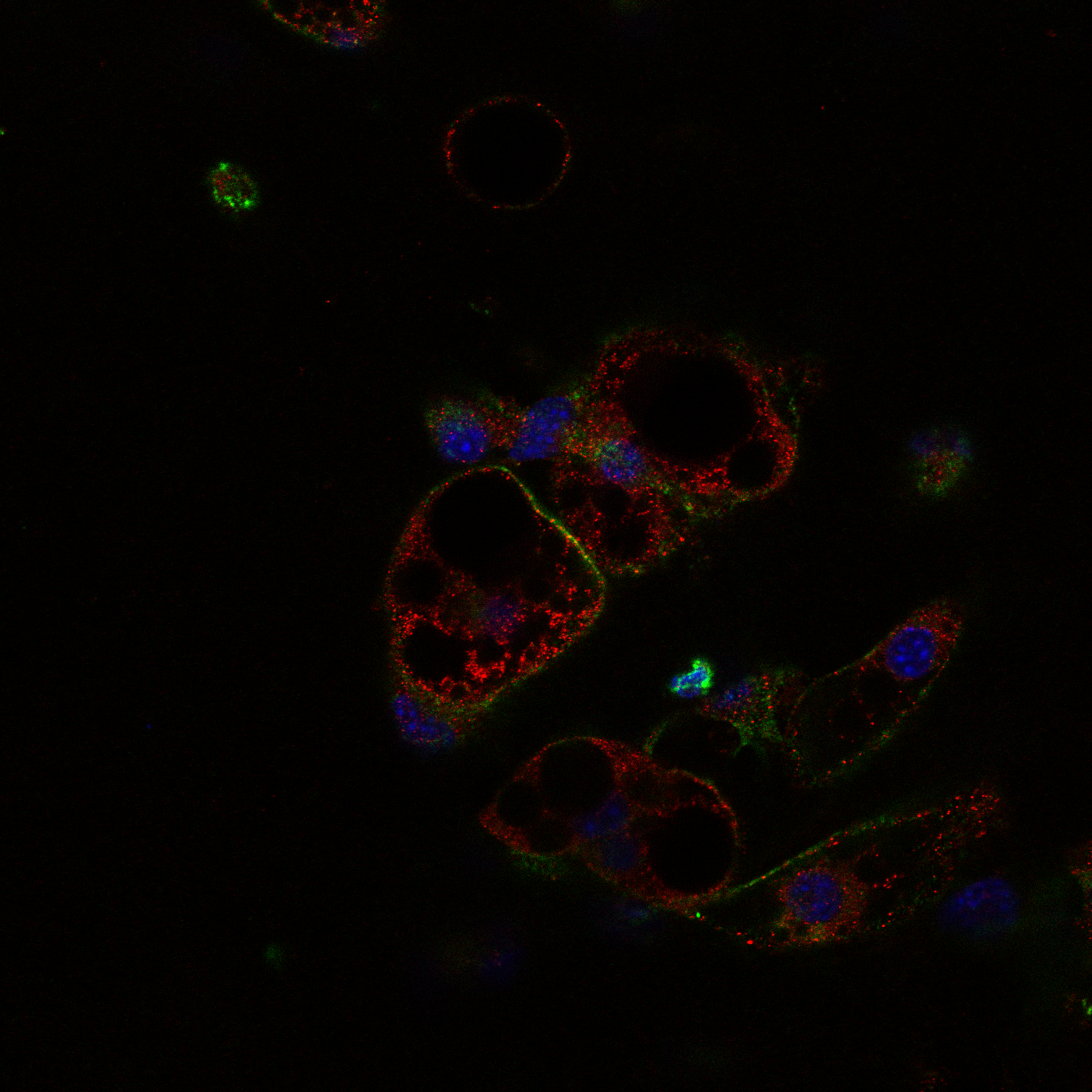

Supplement: Supplementary file 13 — Figure EV6 Source Data [file 44318_2025_520_MOESM13_ESM.zip › EV6/6A/Palmitate_1h.tif]

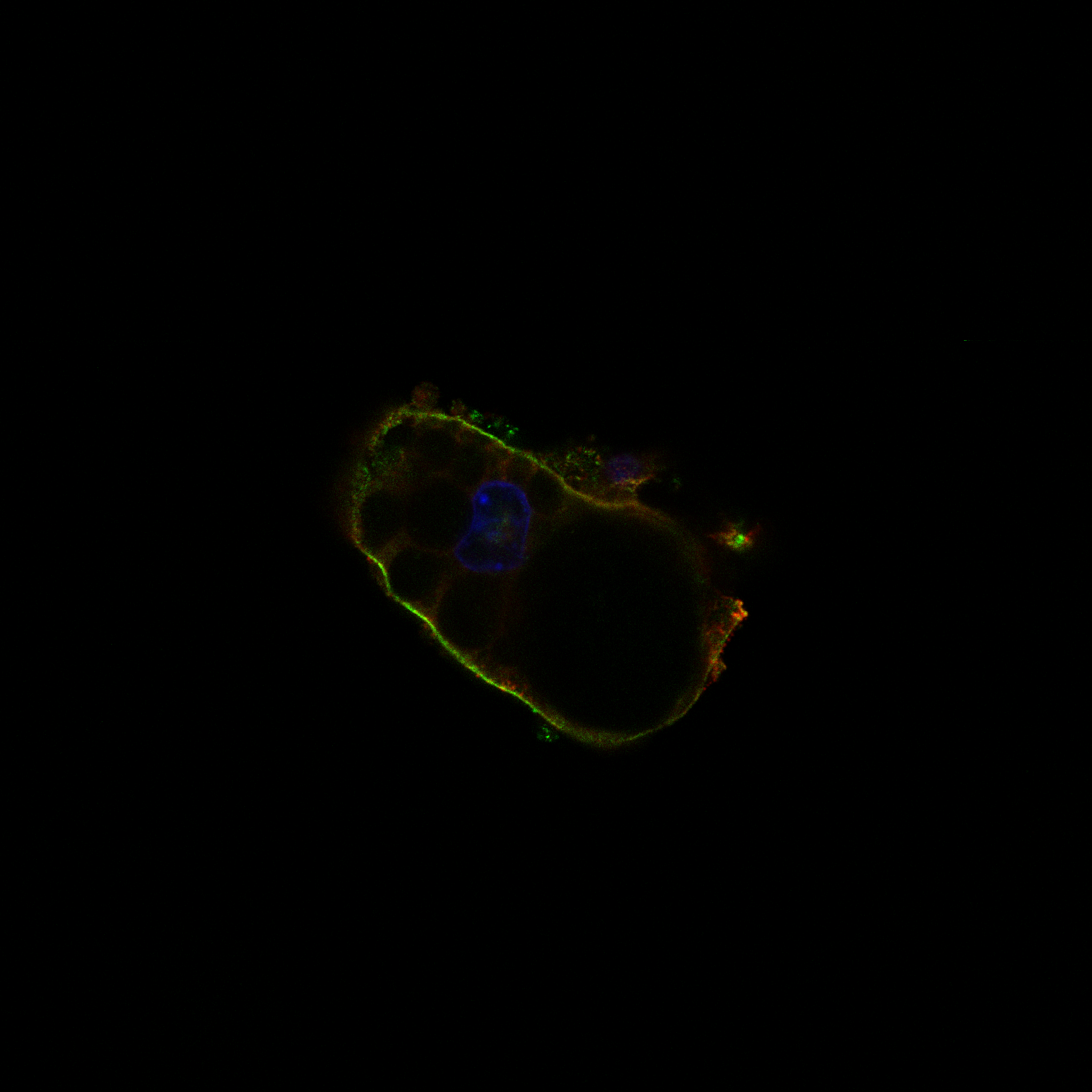

Supplement: Supplementary file 13 — Figure EV6 Source Data [file 44318_2025_520_MOESM13_ESM.zip › EV6/6A/BSA_30min.tif]

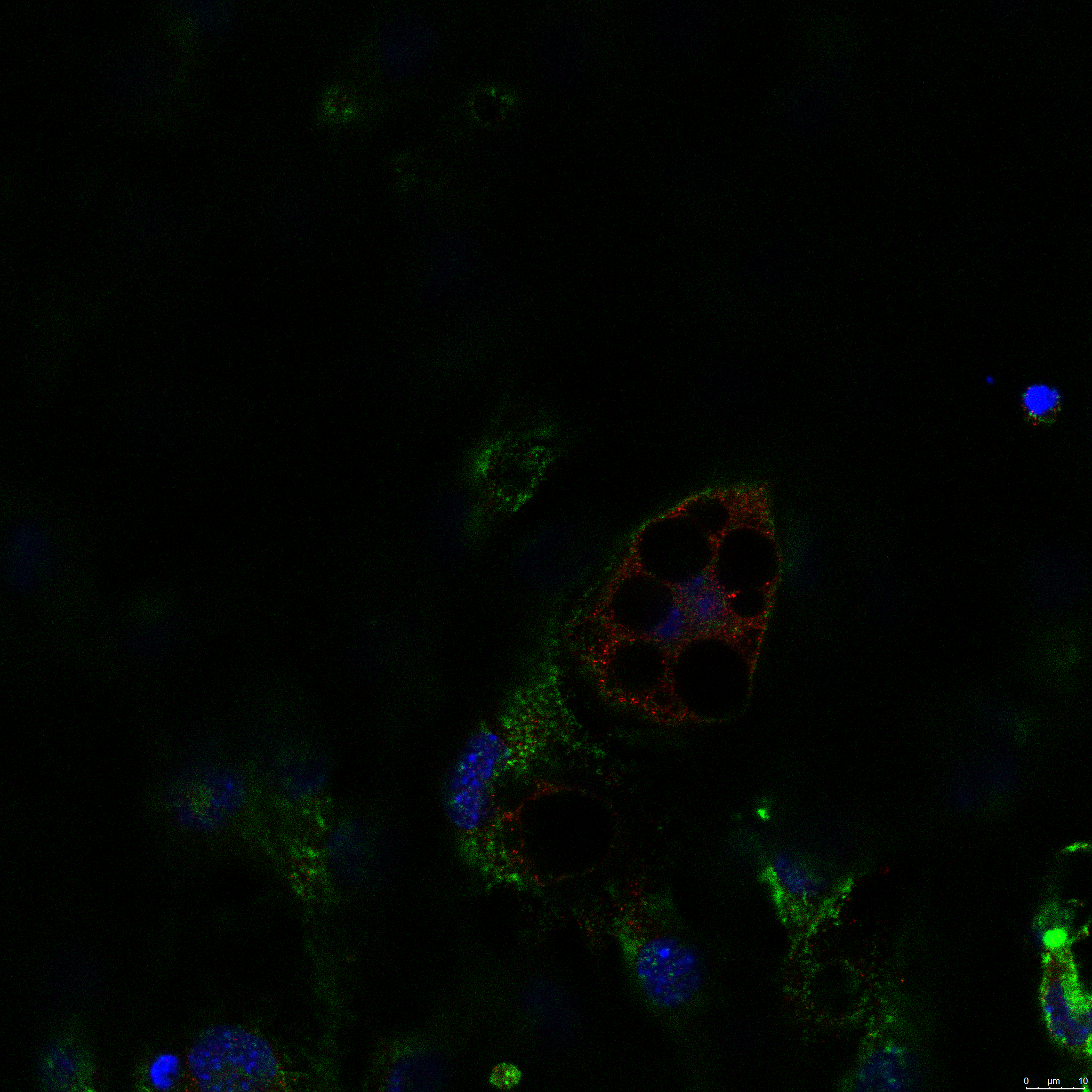

Supplement: Supplementary file 13 — Figure EV6 Source Data [file 44318_2025_520_MOESM13_ESM.zip › EV6/6A/Oleate_1h.tif]

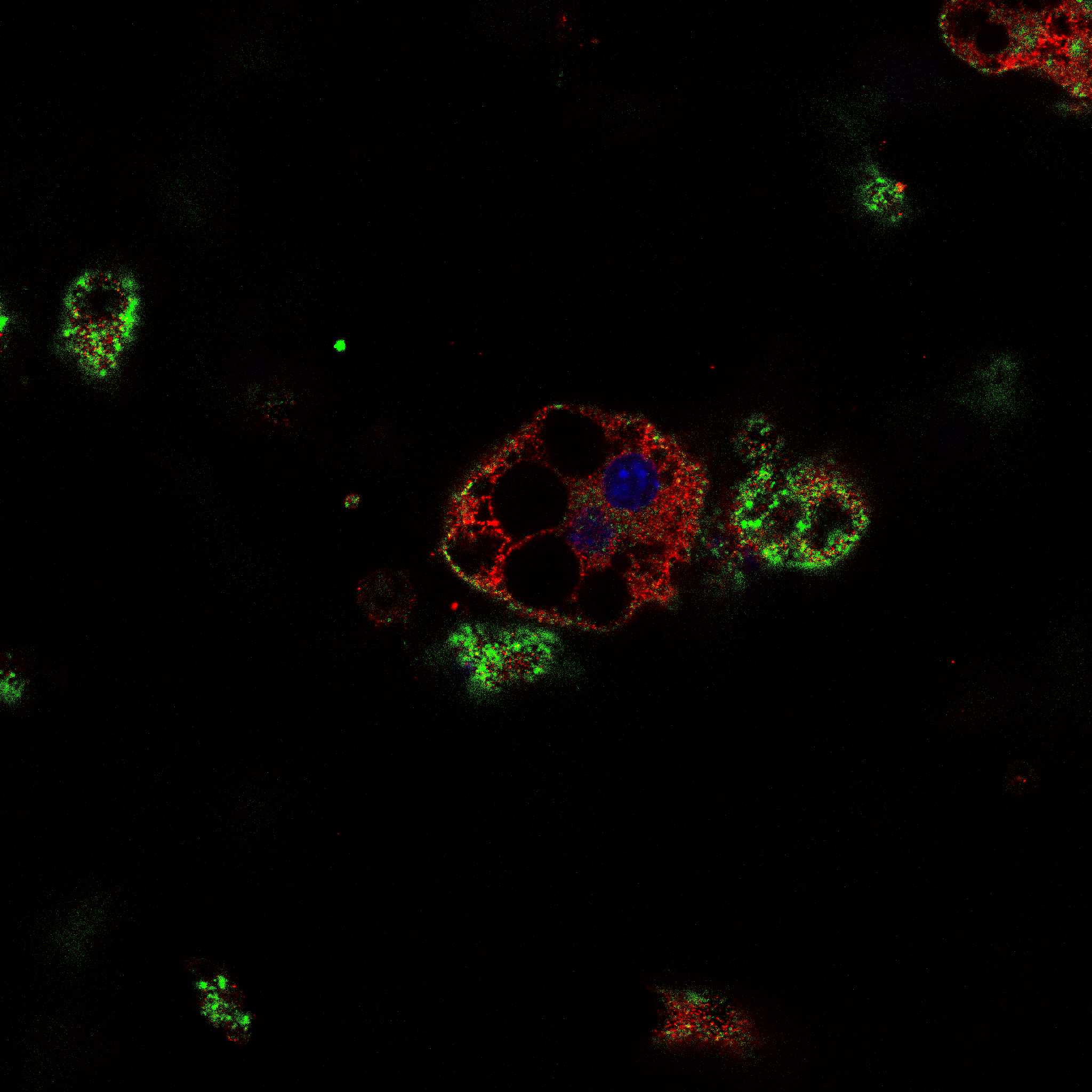

Supplement: Supplementary file 13 — Figure EV6 Source Data [file 44318_2025_520_MOESM13_ESM.zip › EV6/6A/Oleate_30min.tif]

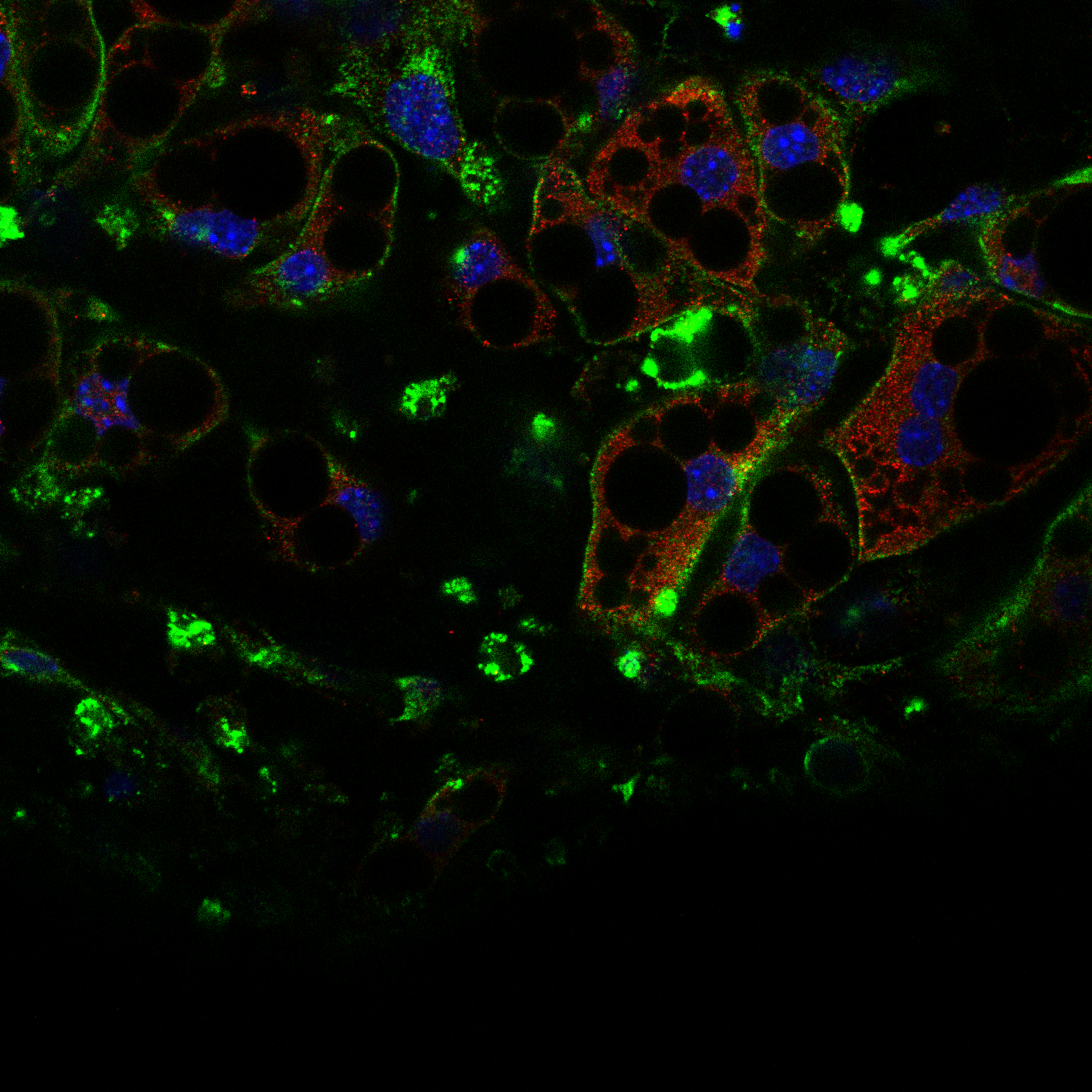

Supplement: Supplementary file 13 — Figure EV6 Source Data [file 44318_2025_520_MOESM13_ESM.zip › EV6/6A/Palmitate_30min.tif]

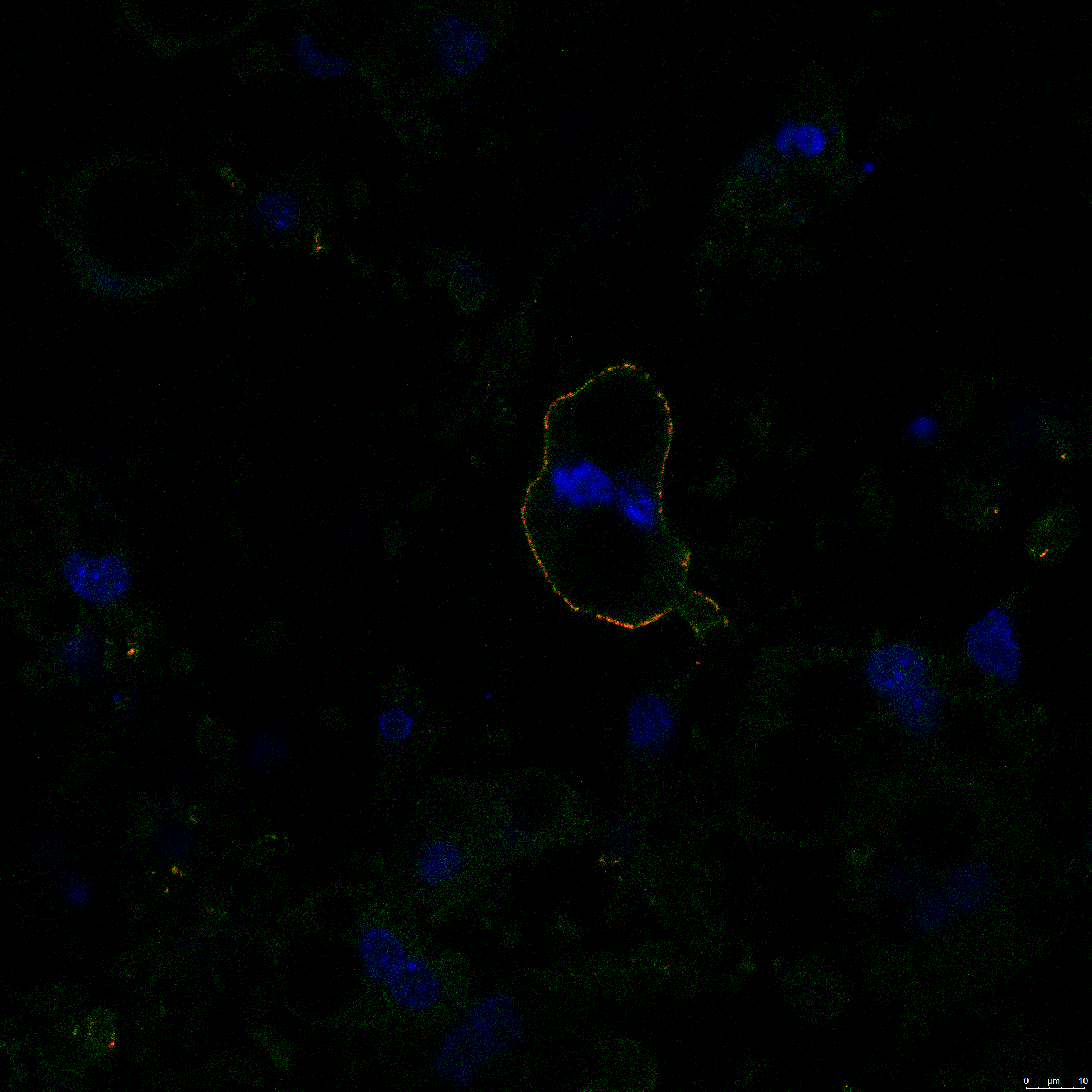

Supplement: Supplementary file 13 — Figure EV6 Source Data [file 44318_2025_520_MOESM13_ESM.zip › EV6/6A/BSA_1h.tif]
