# Supplementary figures and images for: NRAC controls CD36-mediated fatty acid uptake in adipocytes and lipid clearance in vivo (part 2 of 2)
Source: EMBO J. 2025 Aug 1;44(18):5037–65. doi: 10.1038/s44318-025-00520-2 (PMC12436663; doi:10.1038/s44318-025-00520-2)

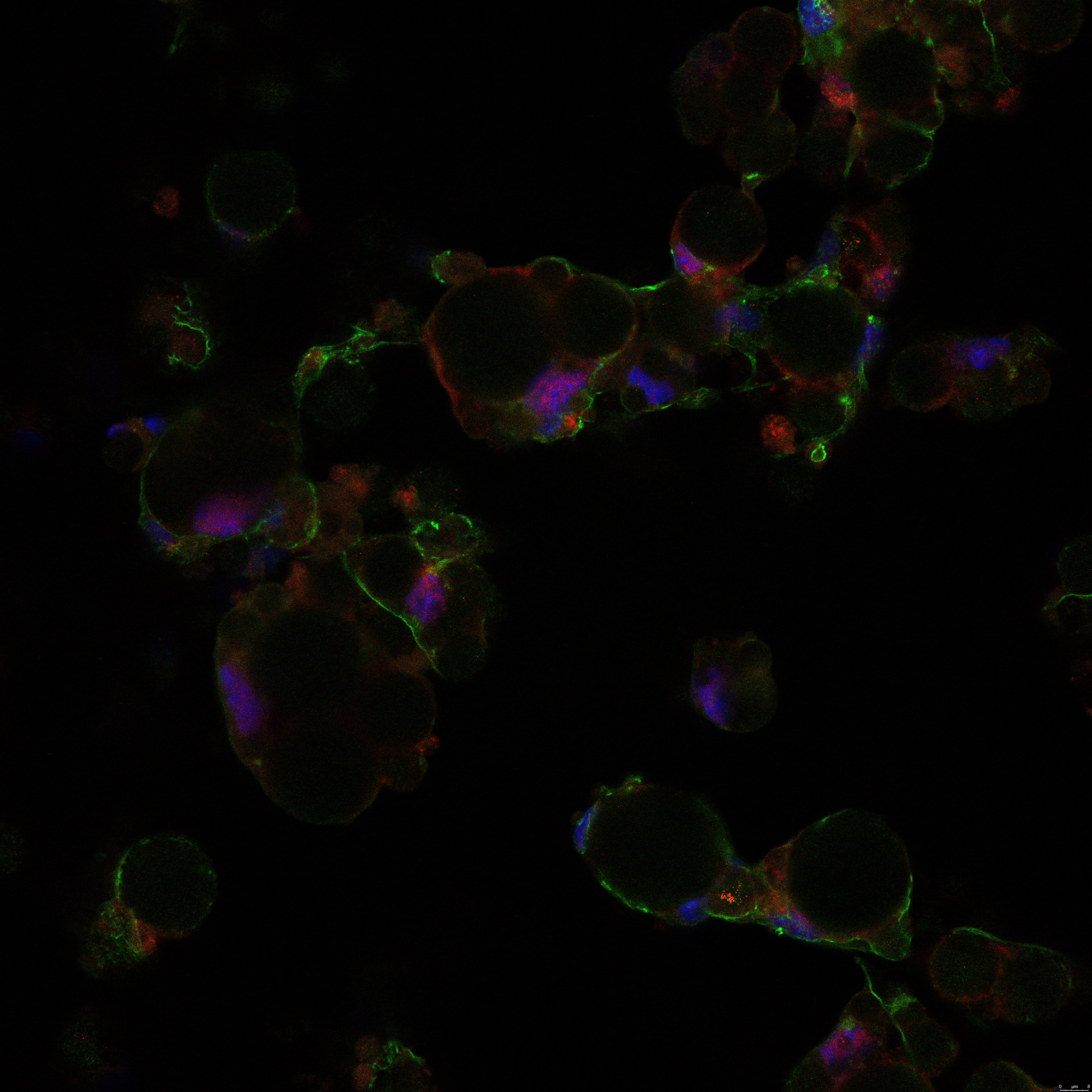

Supplement: Supplementary file 13 — Figure EV6 Source Data [file 44318_2025_520_MOESM13_ESM.zip › EV6/6B/Oleate_100uM.tif]

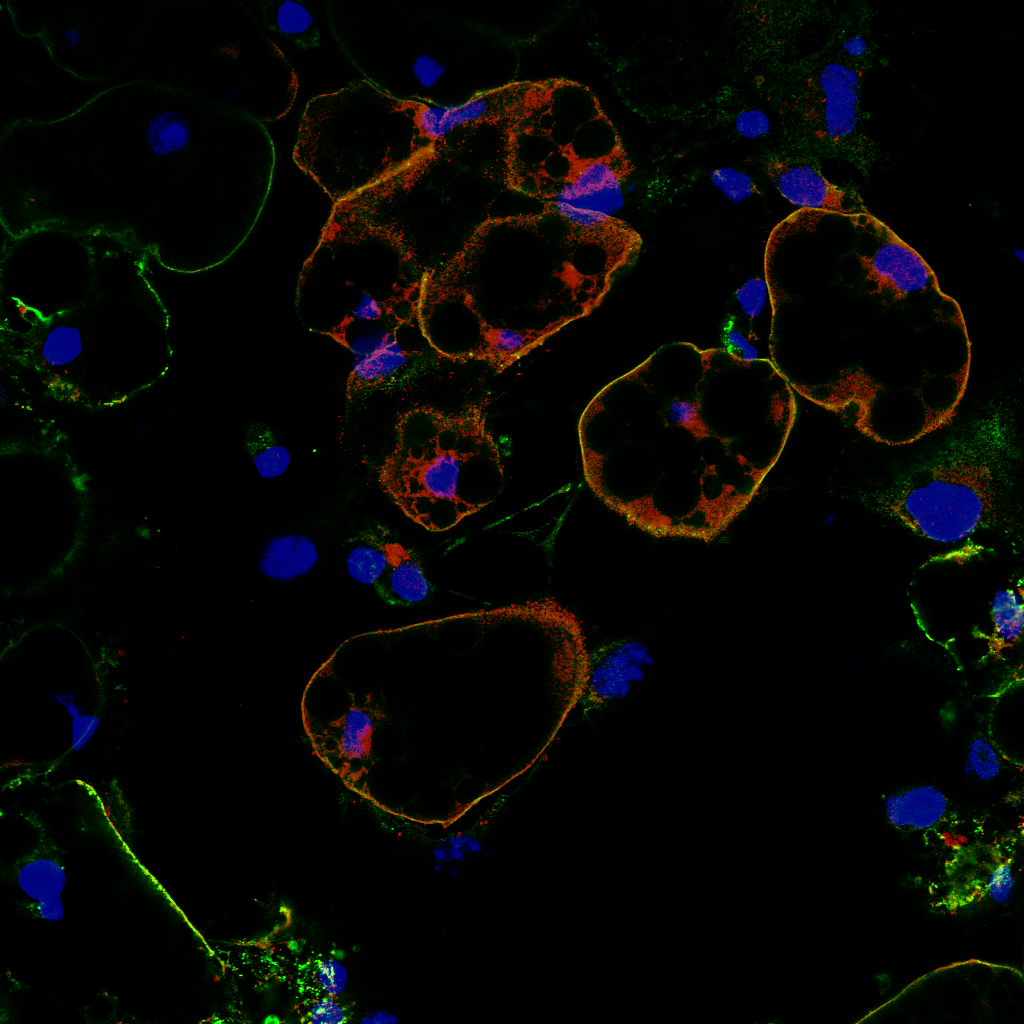

Supplement: Supplementary file 13 — Figure EV6 Source Data [file 44318_2025_520_MOESM13_ESM.zip › EV6/6B/Oleate_10uM.tif]

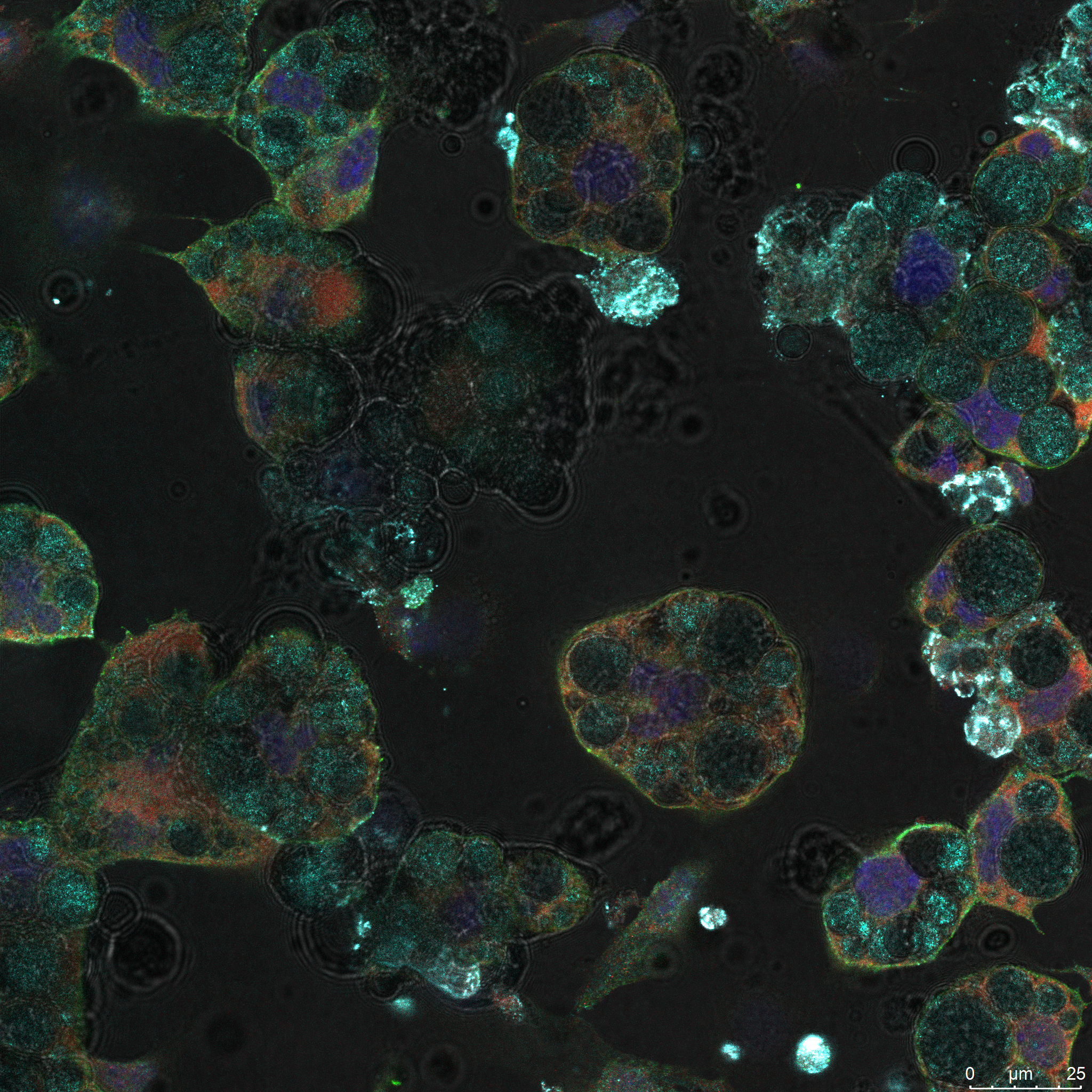

Supplement: Supplementary file 13 — Figure EV6 Source Data [file 44318_2025_520_MOESM13_ESM.zip › EV6/6C/Oleate_WT.tif]

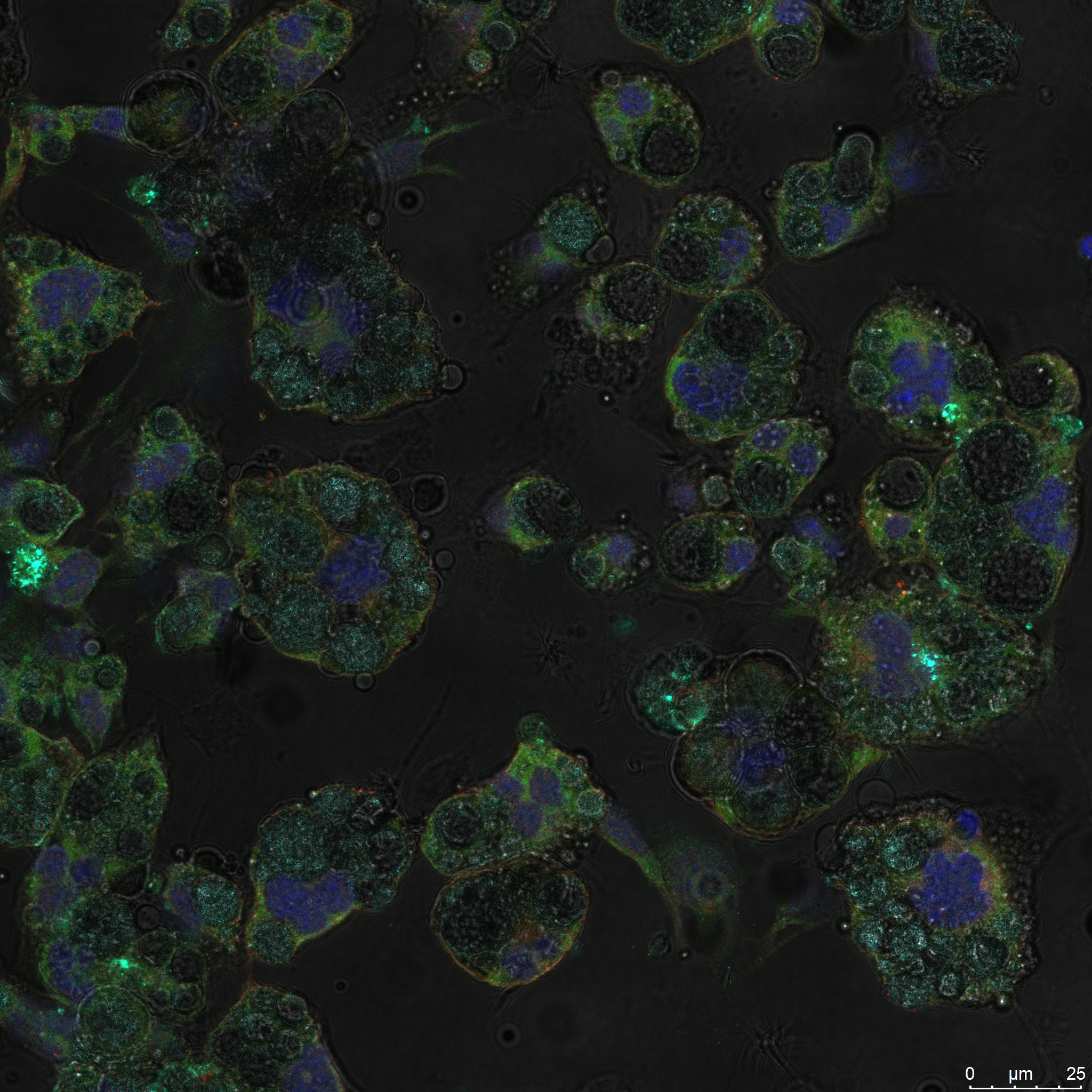

Supplement: Supplementary file 13 — Figure EV6 Source Data [file 44318_2025_520_MOESM13_ESM.zip › EV6/6C/WT_Oleate+CPZ.tif]

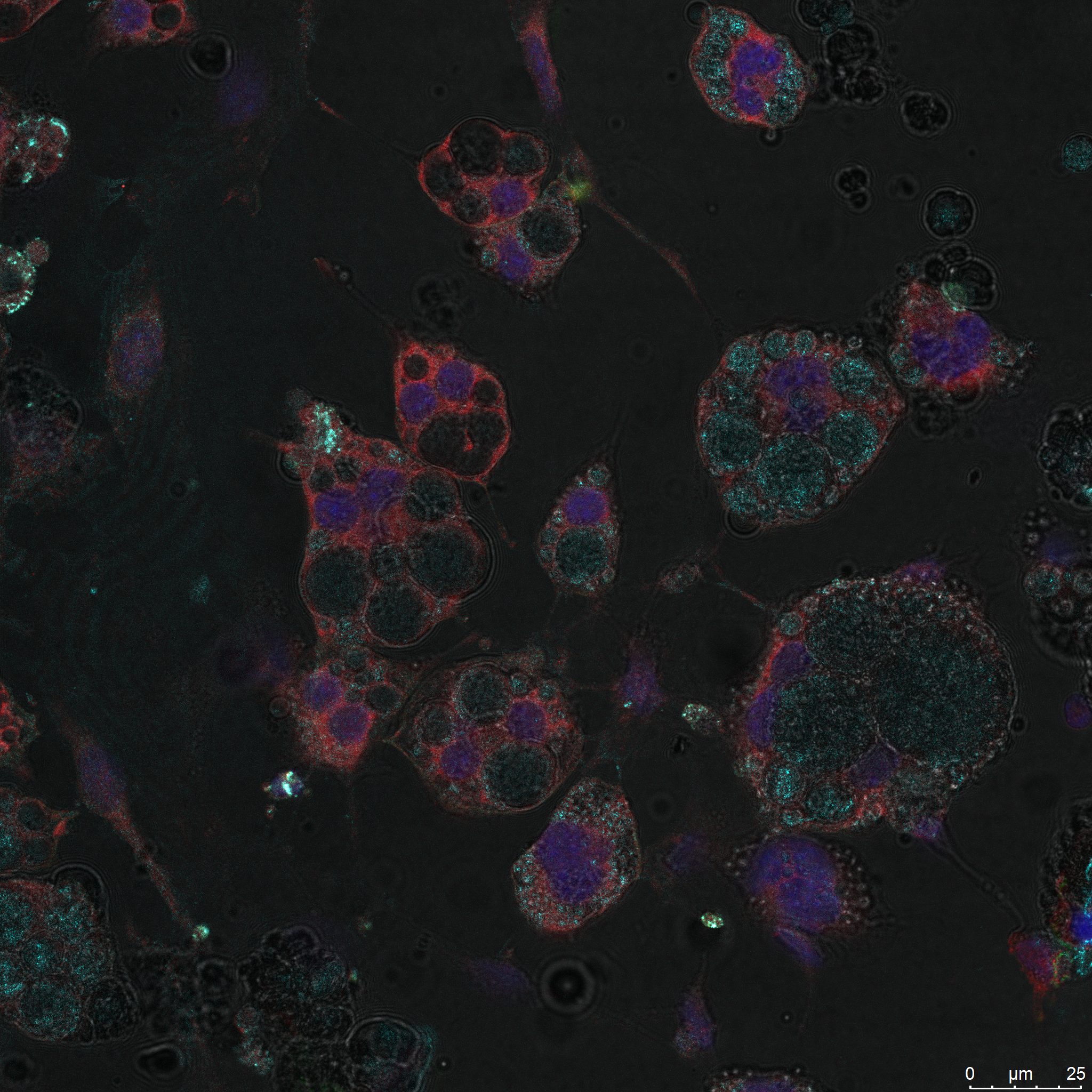

Supplement: Supplementary file 13 — Figure EV6 Source Data [file 44318_2025_520_MOESM13_ESM.zip › EV6/6C/Oleate_KO.tif]

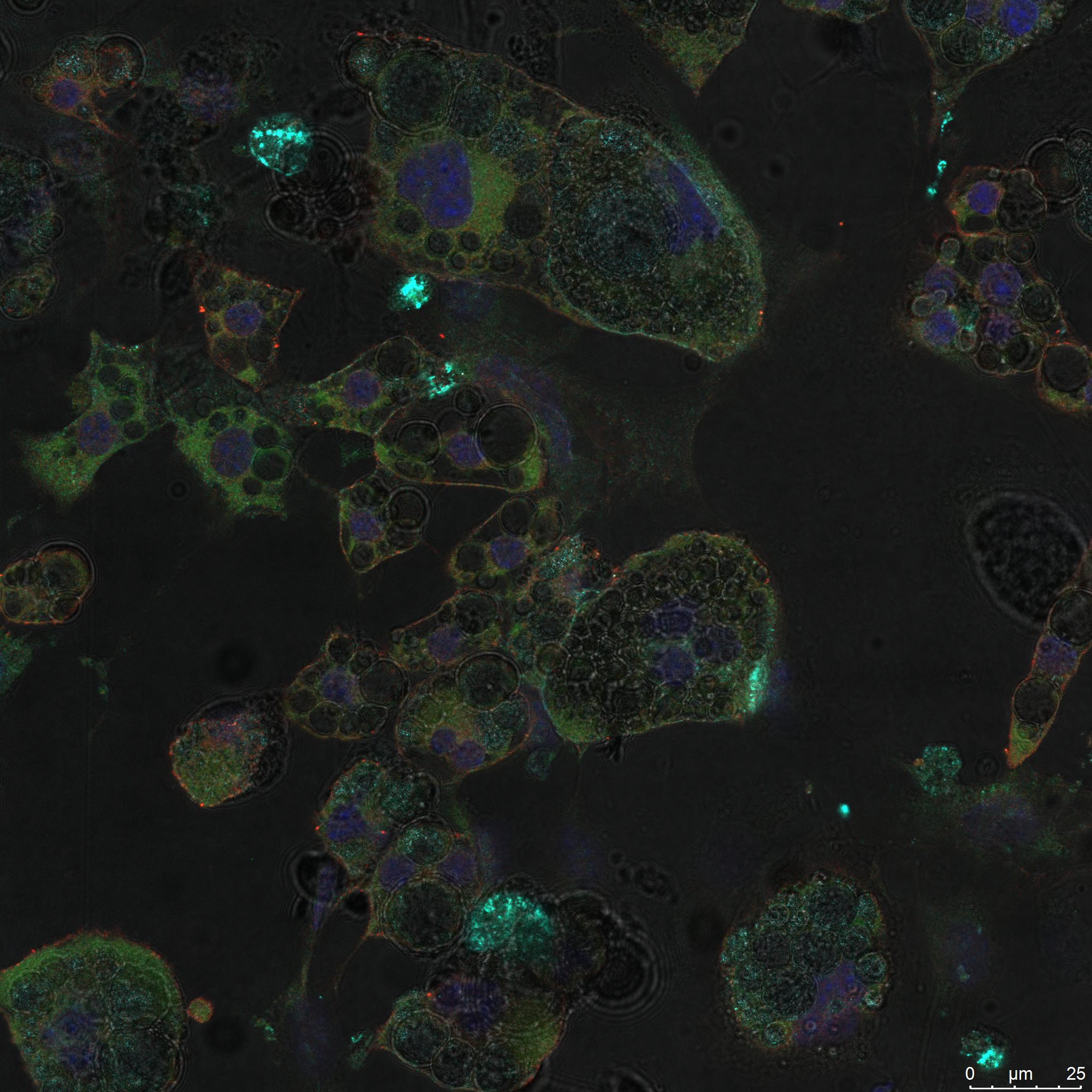

Supplement: Supplementary file 13 — Figure EV6 Source Data [file 44318_2025_520_MOESM13_ESM.zip › EV6/6C/WT_BSA.tif]

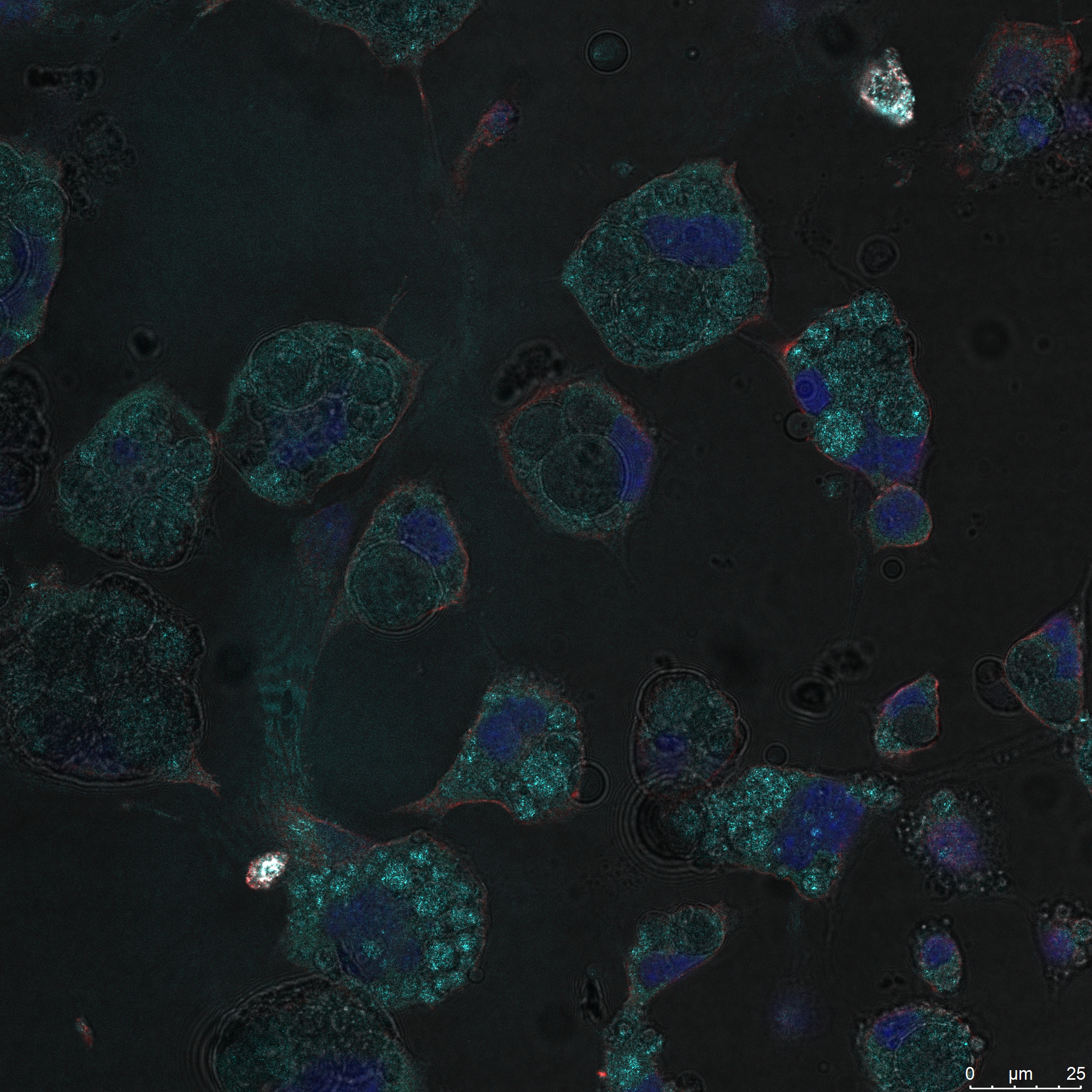

Supplement: Supplementary file 13 — Figure EV6 Source Data [file 44318_2025_520_MOESM13_ESM.zip › EV6/6C/KO_Oleate+CPZ.tif]
