# Supplementary material for: NRAC controls CD36-mediated fatty acid uptake in adipocytes and lipid clearance in vivo
Source: EMBO J. 2025 Aug 1;44(18):5037–65. doi: 10.1038/s44318-025-00520-2 (PMC12436663; doi:10.1038/s44318-025-00520-2)
Supplement: Supplementary file 14 — Expanded View Figures [file 44318_2025_520_MOESM14_ESM.pdf]

Expanded View Figures

Figure EV1. NRAC deletion does not affect glucose metabolism or insulin sensitivity in chow diet fed mice.

Validation of NRAC antibody by (**A**) western blotting of HEK293T cells transfected with either NRAC-GFP or GFP plasmids. Untransfected cells were used as control. (**B**) Western blotting to detect endogenous NRAC in BAT, SCF and PGF and (**C**) immunostaining of subcutaneous differentiated mature adipocytes to detect endogenous NRAC (scale bar 10 μm), and (**D**) Immunoprecipitation of NRAC from perigonadal fat (PGF). (**E–H**) mRNA expression of *Tnfa* and *Il6* in PGF and SCF of male mice ($n = 6$ wt, 5ko), (**I**) weights of liver, heart and pancreas ($n = 9$ wt, 5ko). (**J**) Glucose tolerance test and area under the curve (age 8 weeks). (**K**) Insulin tolerance test (ITT) and area under the curve (AUC) in 9 weeks old male mice. (**L**) Percentage of HbA1c in 12-week-old male mice. (**M**) Body weight development of chow diet fed female wt and Nrac^{ko} mice. (**N**) Fat and (**O**) lean mass of 15-week-old chow diet fed female mice. (**P**) Glucose tolerance test and area under the curve of 8 weeks old female mice. (**Q**) %HbA1c in 12-week-old female mice. (**R**) Insulin tolerance test (ITT) and area under the curve (AUC) in 9-week-old female mice. (**S**) H&E staining of SCF and PGF tissues of female mice (Age 23 weeks), and (**T**) quantification of lipid droplets. $n = 9$wt and 7ko for males and 5wt and 7ko for females. (**U**) mRNA expression of *Nrac* from BAT, SCF and PGF of male and female mice ($n = 4$). Data are shown as mean ± SEM. *$P < 0.05$, **$P < 0.01$, ***$P < 0.001$ by Student's t test or one or two-way ANOVA. Source data are available online for this figure.

© The Author(s)

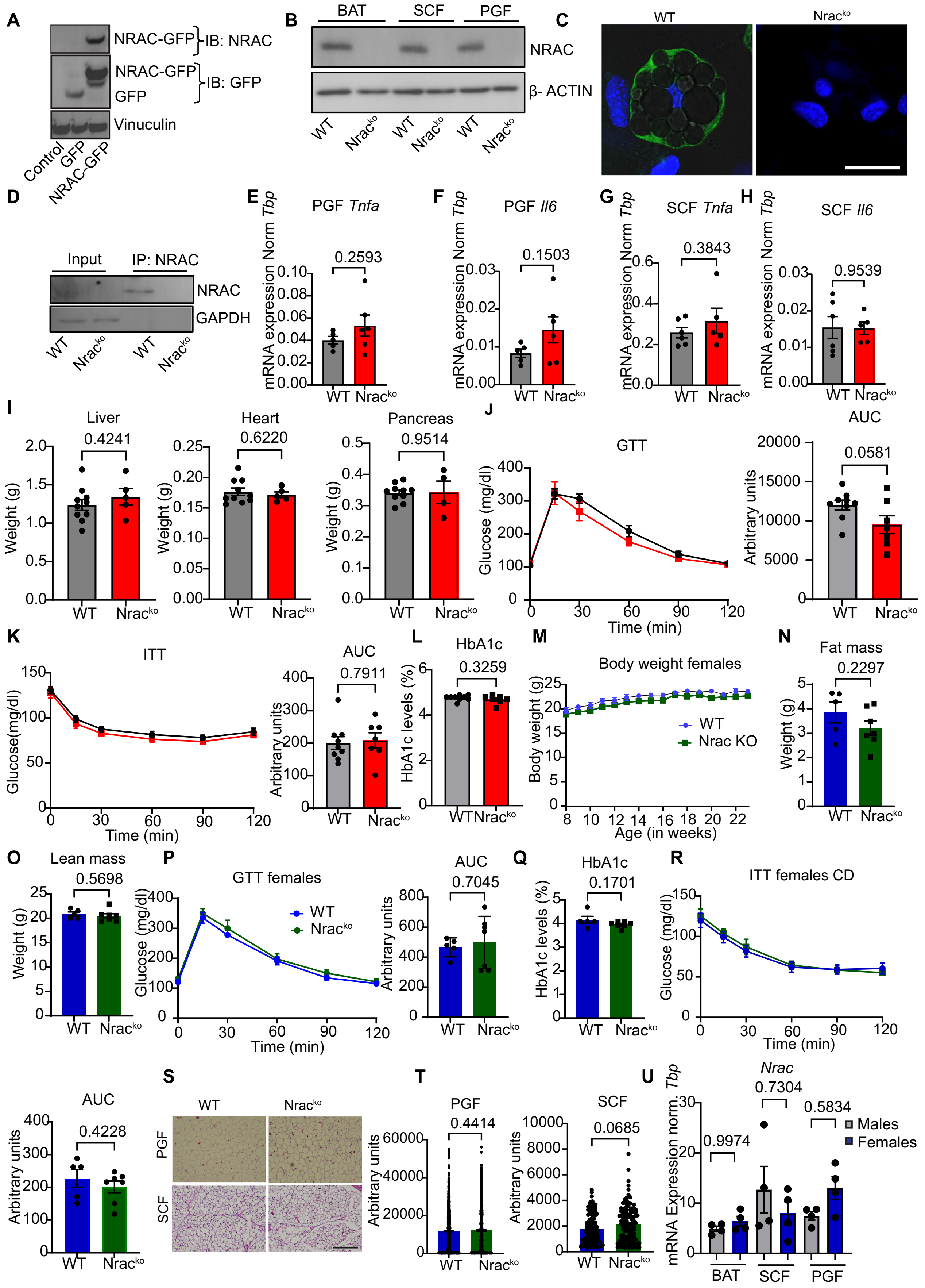
A
NRAC-GFP
IB: NRAC
NRAC-GFP
GFP
IB: GFP
Vinuculin
Control
GFP
NRAC-GFP
B
BAT
SCF
PGF
NRAC
β- ACTIN
WT
Nrac^ko
C
WT
Nrac^ko
D
Input
IP: NRAC
NRAC
GAPDH
E
PGF Tnfa
0.2593
mRNA expression Norm Tbp
F
PGF Il6
0.1503
G
SCF Tnfa
0.3843
H
SCF Il6
0.9539
I
Liver
0.4241
Heart
0.6220
Pancreas
0.9514
Weight (g)
J
GTT
Glucose (mg/dl)
Time (min)
AUC
0.0581
Arbitrary units
K
ITT
Glucose(mg/dl)
AUC
0.7911
L
HbA1c
0.3259
HbA1c levels (%)
M
Body weight females
Body weight (g)
WT
Nrac KO
Age (in weeks)
N
Fat mass
0.2297
O
Lean mass
0.5698
P
GTT females
AUC
0.7045
Q
HbA1c
0.1701
R
ITT females CD
AUC
0.4228
S
PGF
SCF
T
PGF
0.4414
SCF
0.0685
U
Nrac
0.9974
0.7304
0.5834
Males
Females
mRNA Expression norm. Tbp
BAT
SCF
PGF

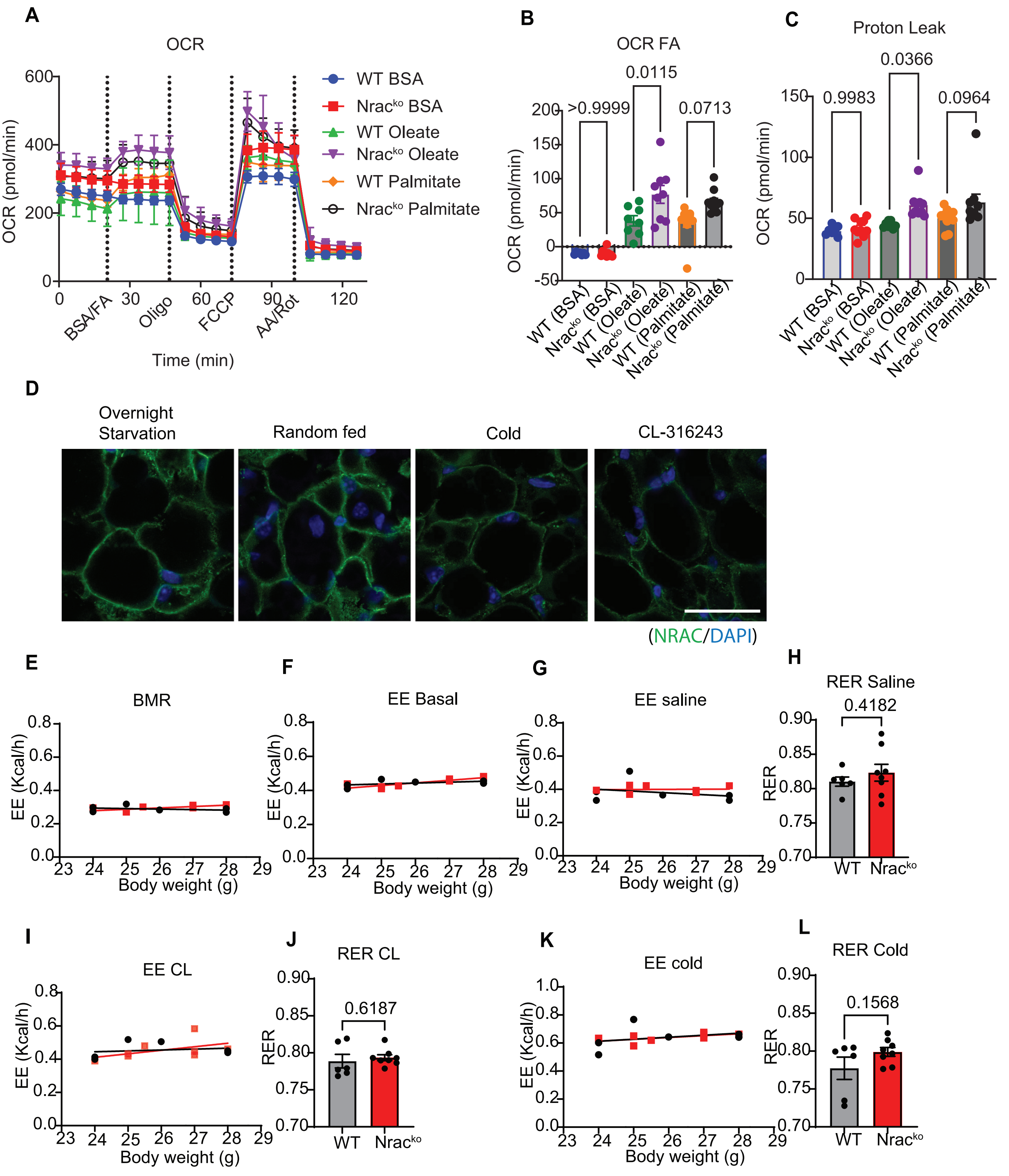
A
OCR
600
400
200
0
OCR (pmol/min)
0
30
60
90
120
BSA/FA
Oligo
FCCP
AA/Rot
Time (min)
WT BSA
Nrac^ko BSA
WT Oleate
Nrac^ko Oleate
WT Palmitate
Nrac^ko Palmitate
B
OCR FA
>0.9999
0.0115
0.0713
200
150
100
50
0
-50
OCR (pmol/min)
WT (BSA)
Nrac^ko (BSA)
WT (Oleate)
Nrac^ko (Oleate)
WT (Palmitate)
Nrac^ko (Palmitate)
C
Proton Leak
0.0366
0.9983
0.0964
150
100
50
0
OCR (pmol/min)
WT (BSA)
Nrac^ko (BSA)
WT (Oleate)
Nrac^ko (Oleate)
WT (Palmitate)
Nrac^ko (Palmitate)
D
Overnight Starvation
Random fed
Cold
CL-316243
(NRAC/DAPI)
E
BMR
EE (Kcal/h)
Body weight (g)
F
EE Basal
EE (Kcal/h)
Body weight (g)
G
EE saline
EE (Kcal/h)
Body weight (g)
H
RER Saline
0.4182
RER
WT
Nrac^ko
I
EE CL
EE (Kcal/h)
Body weight (g)
J
RER CL
0.6187
RER
WT
Nrac^ko
K
EE cold
EE (Kcal/h)
Body weight (g)
L
RER Cold
0.1568
RER
WT
Nrac^ko

 © The Author(s)

◀ **Figure EV2. NRAC deletion does not affect energy homeostasis, food intake and substrate utilization in mice under chow diet.**

(**A**) Oxygen consumption rates (OCR) in wt and Nrac^{ko} differentiated adipocytes. Injection strategy included BSA or BSA conjugated oleate and palmitate (100 μM each) (port 1), oligomycin (port 2), FCCP (port 3) and Antimycin A and rotenone in port 4. (**B**) OCR calculated by subtracting OCR after fatty acid (FA) stimulation from basal respiration. (**C**) Proton leak calculated by subtracting oligomycin OCR from non-mitochondrial OCR ($n = 9$). (**D**) Immunostaining of BAT from wt mice either overnight starved (16 h), random fed, acute cold, (4 h) and CL-316243 injected mice ($n = 3$), age 12 weeks (scale 100 μm). (**E**) Basal metabolic rate (BMR). (**F**) Energy expenditure and respiratory exchange ratio (RER), (**G**, **H**) in saline treated mice, (**I**, **J**) in CL316243 treated mice and (**K**, **L**) upon acute cold exposure ($n = 6$ WT and 8KO, Age 16 weeks). Data are shown as mean ± SEM. *$P < 0.05$, **$P < 0.01$, ***$P < 0.001$ by Student's t test or one or two way ANOVA. In figures (**E–G**, **I**, **K**) the data are plotted as linear regression. Source data are available online for this figure.

© The Author(s)

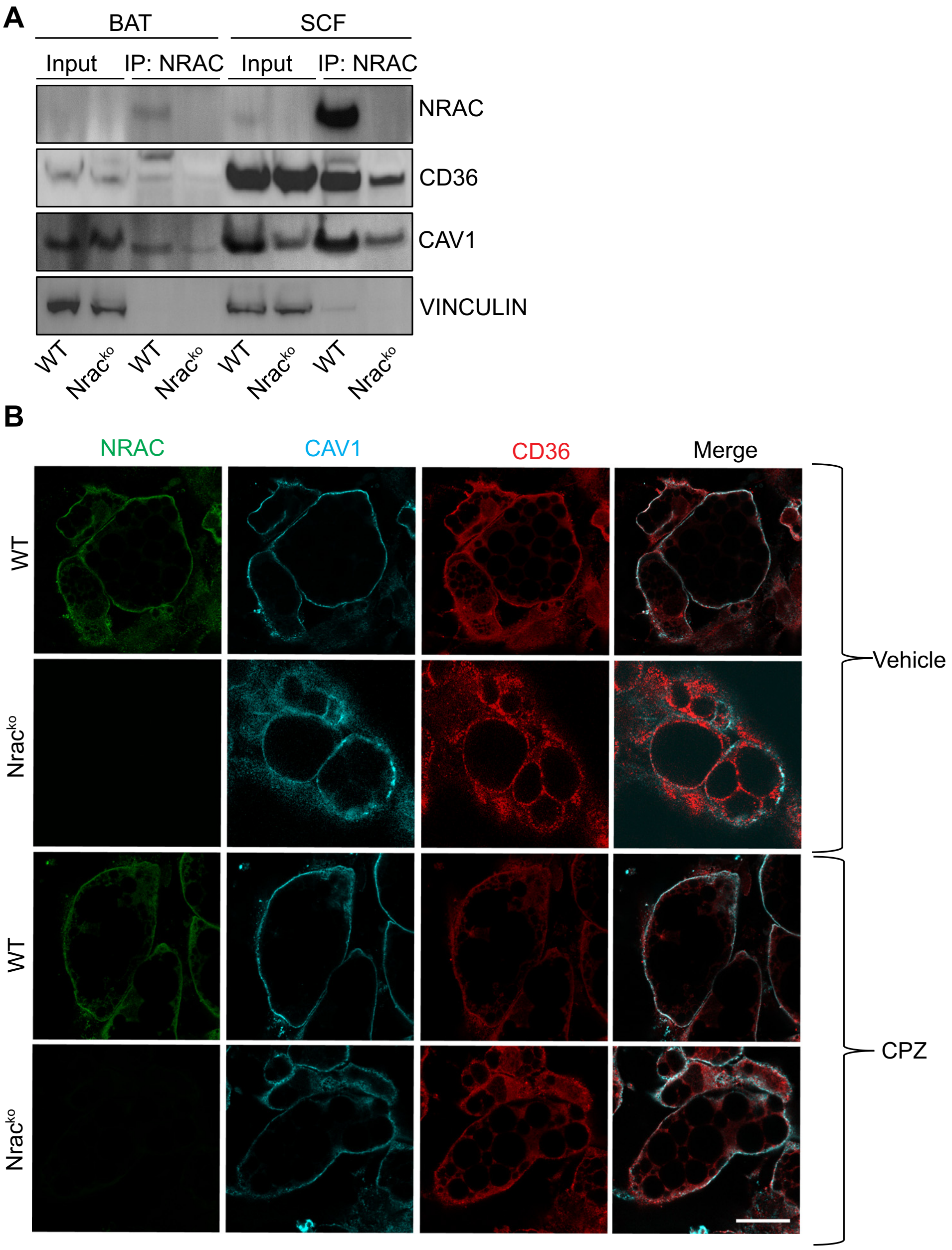

Figure EV3. NRAC regulates CD36/Cav1 interaction in adipocytes.

(**A**) Immunoprecipitation of NRAC from BAT and SCF. (**B**) Immunostaining of differentiated immortalized preadipocytes treated with vehicle (0.05% ethanol) or Chlorpromazine (CPZ) (10 μM) for 1 h (scale 25 μm). Source data are available online for this figure.

 © The Author(s)

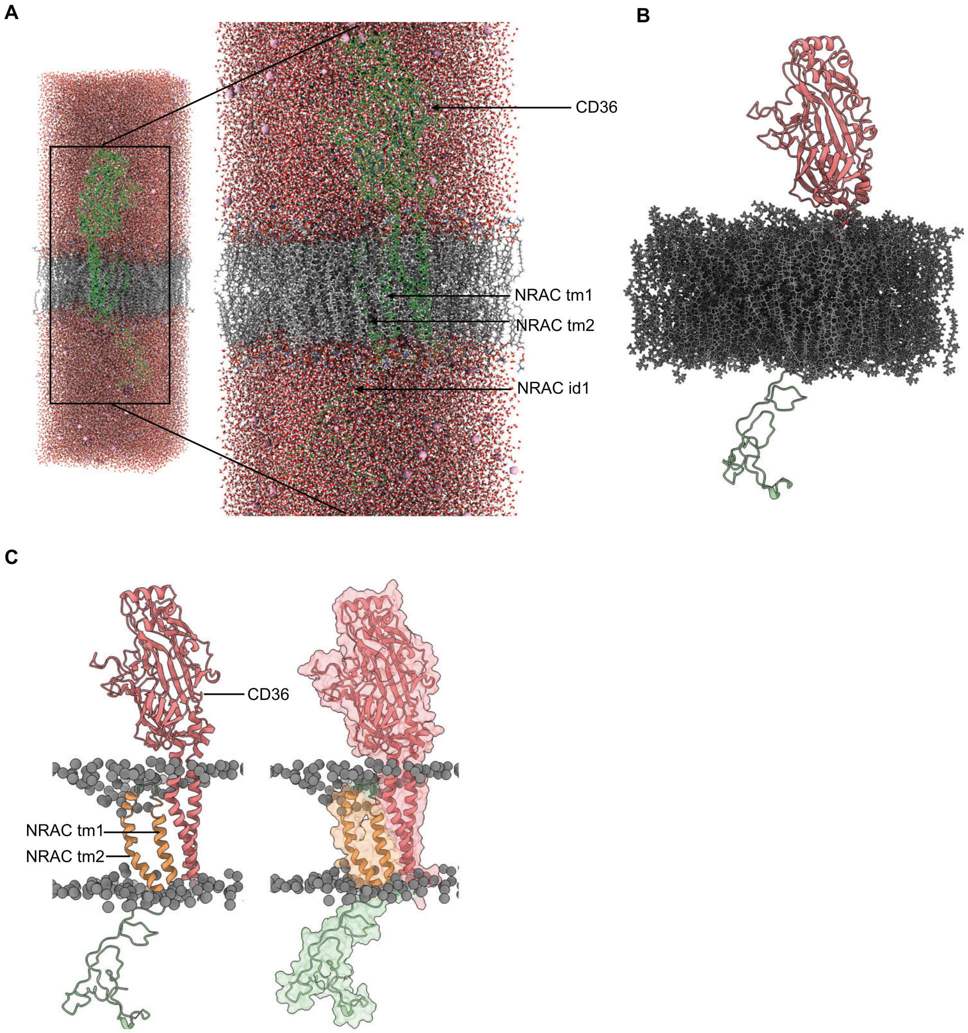

Figure EV4. First transmembrane domain of NRAC is required for NRAC-CD36 interaction.

(**A**) MD-simulations of human NRAC and CD36. The full simulation block is shown, with the central grey stripe representing the lipid bilayer membrane. Water molecules are depicted as red and white particles, while Na^+ and Cl^- ions appear as pink spheres at a physiological concentration of 0.15 M. The bright green structures correspond to the proteins CD36 and NRAC, with CD36 embedded in the upper membrane leaflet and NRAC in the lower leaflet, and the zoomed in area of the same region. (**B**) CD36–NRAC complex is embedded in the membrane, with CD36 located on top and NRAC below. The membrane is shown in grey. (**C**) The CD36–NRAC complex is embedded in the membrane, with CD36 on top and NRAC below. Phospholipids in the membrane are represented as grey circles. Source data are available online for this figure.

© The Author(s)

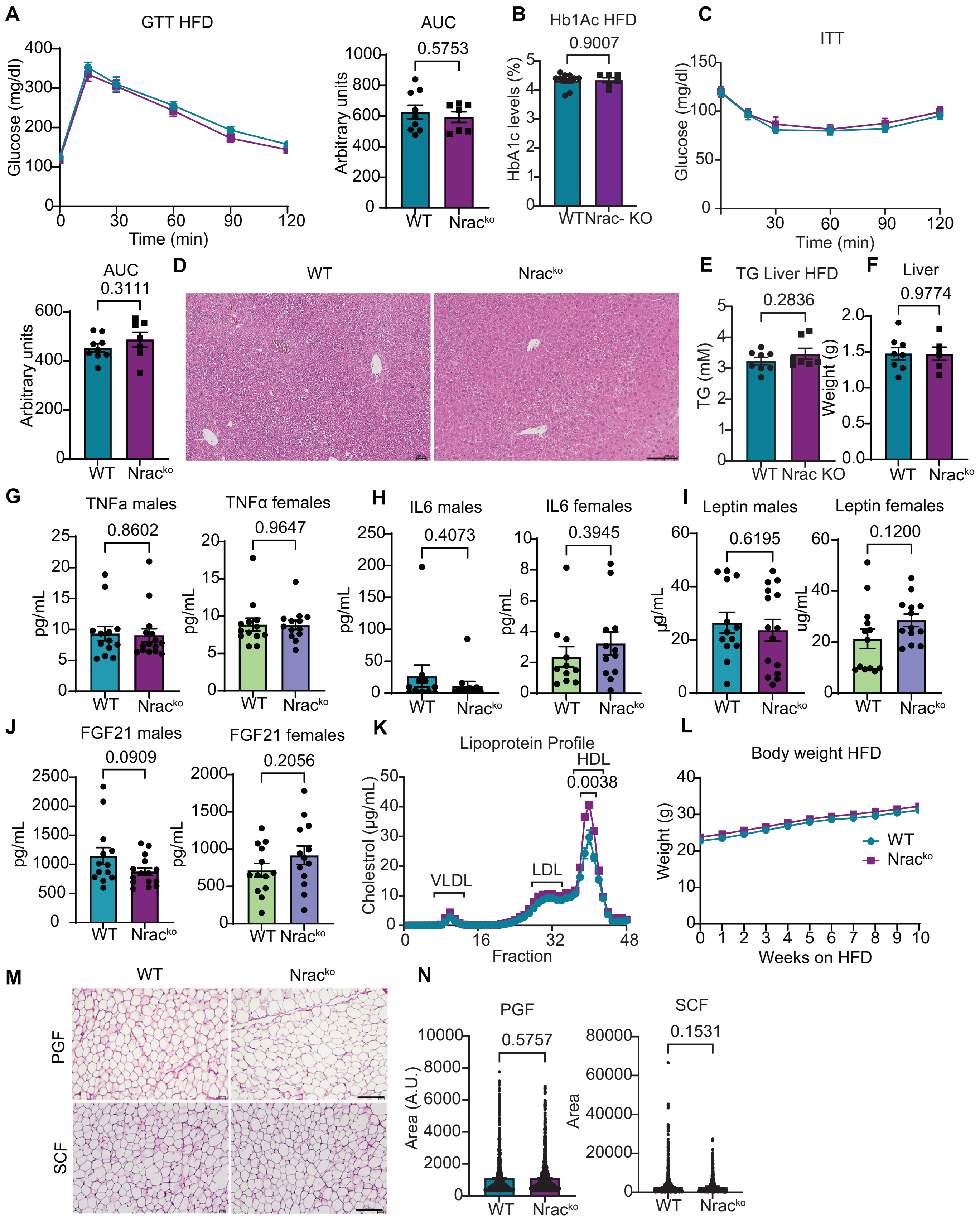
A
GTT HFD
Glucose (mg/dl)
Time (min)
AUC
0.5753
Arbitrary units
WT
Nracko
B
Hb1Ac HFD
0.9007
HbA1c levels (%)
WT
Nrac- KO
C
ITT
Glucose (mg/dl)
Time (min)
AUC
0.3111
Arbitrary units
WT
Nracko
D
WT
Nracko
E
TG Liver HFD
0.2836
TG (mM)
WT
Nrac KO
F
Liver
0.9774
Weight (g)
WT
Nracko
G
TNFa males
0.8602
pg/mL
WT
Nracko
TNFα females
0.9647
pg/mL
WT
Nracko
H
IL6 males
0.4073
pg/mL
WT
Nracko
IL6 females
0.3945
pg/mL
WT
Nracko
I
Leptin males
0.6195
µg/mL
WT
Nracko
Leptin females
0.1200
ug/mL
WT
Nracko
J
FGF21 males
0.0909
pg/mL
WT
Nracko
FGF21 females
0.2056
pg/mL
WT
Nracko
K
Lipoprotein Profile
HDL
0.0038
LDL
VLDL
Cholestrol (µg/mL)
Fraction
L
Body weight HFD
Weight (g)
WT
Nracko
Weeks on HFD
M
WT
Nracko
PGF
SCF
N
PGF
0.5757
Area (A.U.)
WT
Nracko
SCF
0.1531
Area
WT
Nracko

© The Author(s)

Figure EV5. HFD feeding does not affect glucose metabolism, insulin sensitivity and plasma profile of inflammatory cytokines in Nrac^{ko} mice.

(**A**) Glucose tolerance test and baseline corrected area under the curve ($n = 9$ wt, 7 ko, 10 weeks on HFD). (**B**) Percentage of HbA1c in mice13 weeks on HFD ($n = 9$ wt, 7 ko). (**C**) Insulin tolerance test and baseline corrected area under the curve, (12 weeks on HFD, $n = 9$ wt, 7 ko). (**D**) H&E staining of livers of HFD fed mice (17 weeks on HFD), (**E**) Liver triglyceride levels and (**F**) liver weight ($n = 9$ wt, 7 ko, 17 weeks on HFD), Plasma levels of (**G**) TNFα, (**H**) IL6, (**I**) Leptin, and (**J**) FGF21 in male and female non-fasted mice (male: 14 wt, 13 ko; female: 13 wt, 15 ko, 20 weeks on HFD). (**K**) Plasma lipoprotein profile ($n = 5$ wt, 5ko, 10 weeks on HFD), (**L**) Body weight development in HFD fed animals ($n = 8$ wt, 7 ko). Week 0 was the day when the diet was switched from chow to HFD (age 6 weeks). (**M**) H&E staining of PGF and SCF from HFD fed mice (17 weeks on HFD), and (**N**) the quantification of adipocyte cell size. Significance levels are indicated as follows: *$P < 0.05$, **$P < 0.01$, and ***$P < 0.001$. Data are shown as mean ± SEM. *$P < 0.05$, **$P < 0.01$, ***$P < 0.001$ by Student's *t* test in all figures except (**A**, **C**, **K**, **L**) where two-way ANOVA was used. Source data are available online for this figure.

© The Author(s)

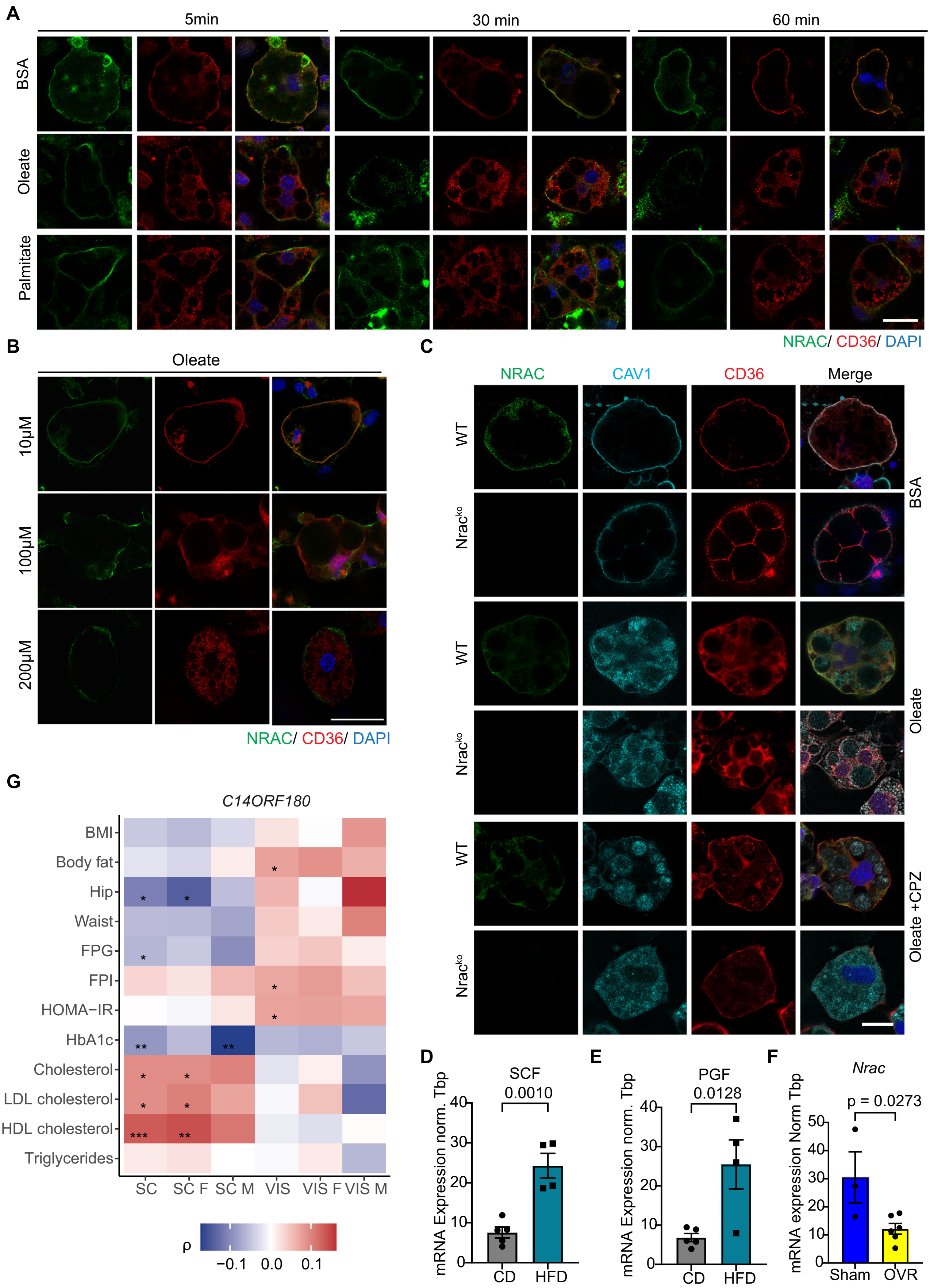
A
5min
30 min
60 min
BSA
Oleate
Palmitate
NRAC/ CD36/ DAPI
B
Oleate
10µM
100µM
200µM
NRAC/ CD36/ DAPI
C
NRAC
CAV1
CD36
Merge
WT
Nracko
BSA
Oleate
Oleate +CPZ
G
C14ORF180
BMI
Body fat
Hip
Waist
FPG
FPI
HOMA−IR
HbA1c
Cholesterol
LDL cholesterol
HDL cholesterol
Triglycerides
SC
SC F
SC M
VIS
VIS F
VIS M
ρ
−0.1 0.0 0.1
D
SCF
0.0010
mRNA Expression norm. Tbp
CD
HFD
E
PGF
0.0128
F
Nrac
p = 0.0273
mRNA expression Norm Tbp
Sham
OVR

Figure EV6. Fatty acid stimulation reduces NRAC surface localization in adipocytes.

(**A**) Immunostaining of differentiated subcutaneous adipocytes, treated with BSA, Oleate (100 µM) and palmitate (100 µM) for 5, 30 and 60 min. On day 8 of differentiation, cells were starved with serum free medium for 1 h and treated with fatty acids for the indicated times. (**B**) Immunostaining of differentiated subcutaneous adipocytes on day 8 of differentiation. Cells were starved with serum free medium 1 h and treated with 10, 100 and 200 µM BSA conjugated oleate. (**C**) Immunostaining of differentiated adipocytes treated either with oleate (100 µM) or with oleate (100 µM) and CPZ (10 µM) for one hour after 1 h serum starvation. *Nrac* mRNA expression in (**D**) subcutaneous (SCF) and (**E**) perigonadal (PGF) adipose tissue from CD and HFD fed mice ($n = 5$ CD and 4 HFD), (**F**) *Nrac* mRNA expression of sham and ovariectomized (OVR) HFD fed mice ($n = 3$ sham, 6 ovariectomized). (**G**) The correlation of C14orf180 (human *NRAC*) gene expression with clinical parameters was analyzed in both subcutaneous (SC) and visceral (VIS) adipose tissue across all individuals, as well as separately for male (M) and female (F) participants. Spearman correlations were calculated, and *P* values were adjusted for false discovery rate. Significance levels are indicated as follows: *$P < 0.05$, **$P < 0.01$, and ***$P < 0.001$. Data are shown as mean ± SEM. *$P < 0.05$, **$P < 0.01$, ***$P < 0.001$ by Student's *t* test in all figures except (**A**, **C**, **K**, **L**) where two-way ANOVA was used. Source data are available online for this figure.

© The Author(s)
